# **Iron-Catalyzed H/D Exchange of Primary Silanes, Secondary Silanes, and Tertiary Siloxanes**

**DOI:** 10.1021/acscatal.2c00224

**Published:** 2022-02-18

**Authors:** Thomas G. Linford-Wood, Mary F. Mahon, Matthew N. Grayson, Ruth L. Webster

**Affiliations:** Department of Chemistry, University of Bath, Claverton Down, Bath BA2 7AY, U.K.

**Keywords:** iron catalysis, silanes, siloxanes, deuterium labeling, density functional theory, mechanistic investigations

## Abstract

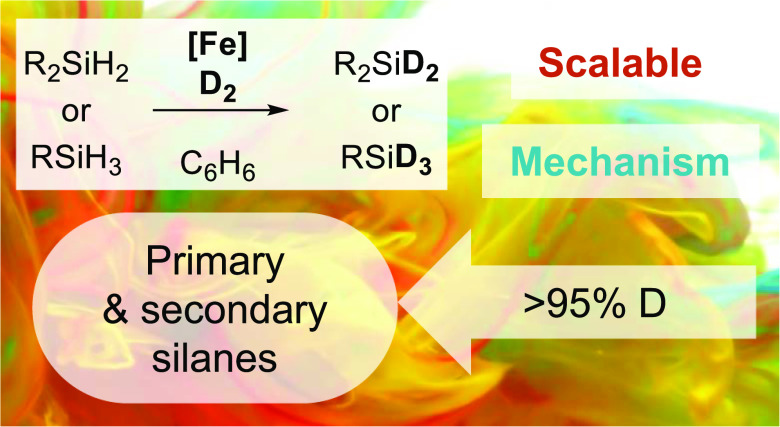

A synthetic study into the catalytic hydrogen/deuterium (H/D) exchange of 1° silanes, 2° silanes, and 3° siloxanes is presented, facilitated by iron-β-diketiminato complexes (**1a** and **1b**). Near-complete H/D exchange is observed for a variety of aryl- and alkyl-containing hydrosilanes and hydrosiloxanes. The reaction tolerates alternative hydride source pinacolborane (HBpin), with quantitative H/D exchange. A synthetic and density functional theory (DFT) investigation suggests that a monomeric iron-deuteride is responsible for the H/D exchange.

Deuterium labeling is of fundamental importance in medicinal^1−5^ and synthetic chemistry.^6−10^ Deuterosilanes are frequently deployed as reagents for isotopic labeling, including but not limited to, hydrosilylation reactions,^11−16^*ortho*-silylations,^17^ reductions of carbon-halogen^18,19^ and carbon–carbon multiple bonds,^20−22^ and are a key mechanistic probe when investigating the deuterium kinetic isotope effect of a reaction. Deuterosilanes are traditionally generated by the reaction of hazardous NaBD_4_ or LiAlD_4_ with corresponding chlorosilane, generating stoichiometric quantities of waste metal salts.^23^ With increasing demand for deuterium-labeled products, catalysis provides a promising solution because of its inherently improved selectivity, functional group tolerance, and reduced waste.^24−26^ Therefore, finding catalysts that facilitate the hydrogen/deuterium (H/D) exchange of hydrosilanes is desirable.

Thus far, catalytic deuteration of silanes has been limited to precious metals^27−40^ and photocatalysis.^26,41^ Furthermore, most of these reports are limited to the H/D exchange of 3° silanes. Three examples are known where activity is retained for 1° and 2° silanes, and with the exception of Carmona and co-workers’ study, these are limited to aromatic hydrosilanes.^32,33,38^ In 2010, Carmona and co-workers reported the H/D exchange of silanes catalyzed by a cationic rhodium complex and D_2_ (Scheme 1a, left). This study included 1° and 2° silanes PhSiD_3_, Ph_2_SiD_2_, and Et_2_SiD_2_, all reaching ≥99% H/D exchange. Three loadings of D_2_ were required to achieve full deuterium incorporation. In 2011, Nolan and co-workers described an iridium-catalyzed H/D isotopic exchange of hydrosilanes with D_2_ (Scheme 1a, middle). The reactivity of one 2° silane was reported, Ph_2_SiD_2_, achieving 95% deuterium exchange. In 2017, Apeloig and co-workers reported silane deuteration catalyzed by a simple platinum complex and D_2_ (Scheme 1a, right). 1° and 2° silanes were limited to PhSiD_3_ and MePhSiD_2_, both achieving 90% H/D exchange. Finding a consistent method for the catalytic H/D exchange of 1° and 2° silanes remains a challenge. Furthermore, to the best of our knowledge, a method utilizing earth-abundant metals has not been reported.

**Scheme 1:**
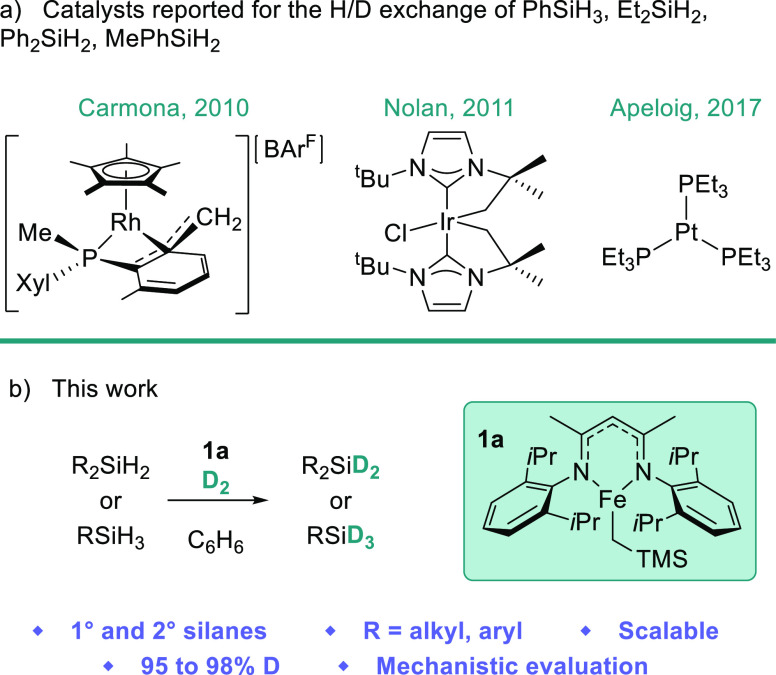
**(**a) Catalysts That Facilitate H/D Exchange of 1° and 2° Silanes Are Based around Precious Metals (Rh, Ir, Pt). (b) This Work Focuses on an Fe(II) Precatalyst (**1a**) for the Deuteration of a Range of 1° and 2° Hydrosilanes

The vast natural abundance of iron makes it a cheaper and more sustainable alternative to precious metals.^42−44^ Its reduced toxicity makes its use in the pharmaceutical industry appealing.

The potential for the reactive, three-coordinate, iron-β-diketiminato complex (^dipp^BDKFeCH_2_TMS, **1a**) to facilitate silane H/D exchange was evidenced during our previous study of dehydrocoupling.^45^ When **1a**, aniline, and methylphenylsilane were exposed to 1 atm of D_2_, H/D exchange was observed at silicon in the generated silazane. Herein, we report the first example of iron-catalyzed H/D exchange of silanes (Scheme 1b).

Based on our investigation into dehydrocoupling of silanes with amines, we began by reacting MePhSiH_2_ (**2a-H**) with D_2_ in the presence of 5 mol % **1a** under 1 atm of D_2_ (Table 1, Entry 1).^45^ After 16 h, almost complete consumption of the Si–H peak is observed in the ^1^H NMR spectrum. Deuteration was confirmed by the introduction of the corresponding peak in the ^2^H NMR spectrum and the appearance of a quintet in the ^29^Si NMR spectrum, arising from ^1^J_Si-D_ coupling to two quadrupolar deuterium atoms. Nuclear magnetic resonance (NMR) analysis reveals a deuterium incorporation of 92%. **1a** is required to catalyze the H/D exchange (Entry 2). Lewis-acidic Fe(acac)_2_ is unable to affect deuteration (Entry 3) while FeCl_2_·(THF)_1.5_, ^dipp^BDKFe(μ-Cl)_2_Li(THF)_2_, and ^dipp^BDK (precursors to **1a**) are unable to impart any H/D exchange (Entries 4–6). Replenishing deuterium in the vessel after 16 h gives no improvement in deuterium incorporation (Entry 7). Finally, optimal deuterium incorporation is achieved when filling the ampoule over liquid N_2_, achieving 97% after 16 h (Entry 8). We estimate that this is equivalent to 4 atm. D_2_ in the vessel.

**Table 1:**
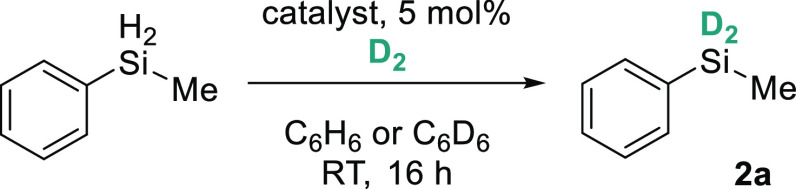
Optimization of Silane Deuteration^a^

entry	catalyst	D incorporation (%)^b^	spec. yield (%)^c^
1	**1a**	92	93
2	none	0	0
3	Fe(acac)_2_	0	0
4	FeCl_2_·THF_1.5_	0	0
5	^dipp^BDKFe(μ-Cl)_2_ Li(THF)_2_	0	0
6	^dipp^BDK	0	0
7^d^	**1a**	87	96
8^e^	**1a**	97	95

a Conditions: 60 mL ampoule containing methylphenylsilane (0.25 mmol), catalyst (5 mol %), D_2_ (after free-pump-thaw cycle), C_6_H_6_ or C_6_D_6_ (0.5 mL), RT, 16 h.

b Determined by ^1^H NMR spectra comparing residual Si–H to Si–CH_3_ or ^m^Ar–H after vacuum distillation and ^2^H NMR spectroscopy.

c Spectroscopic yield determined by ^1^H NMR spectroscopy by comparing Si–CH_3_ or ^m^Ar–H to 1,3,5-trimethoxybenzene (TMB, 0.25 mmol) as the internal standard after vacuum distillation.

d Freeze-pump-thawed after 16 h, refilled with D_2_ (1 atm), and stirred for a further 4 h.

e D_2_ filled over liquid nitrogen (4 atm).

With the optimal conditions in hand (Table 1, Entry 8), we began exploring the substrate scope (Table 2). 1° and 2° aromatic silanes react well under the optimized conditions, with deuterium incorporation of 95 and 97%, respectively (Entries 2 and 3a). After a 10-fold scale-up, **2c** is isolated in 92% yield with corresponding deuterium incorporation of 96% (Entry 3b). The reaction also tolerates the aliphatic hydrocarbon solvent (pentane) with deuterium incorporation of 97% (Entry 3c). 1° and 2° aliphatic deuterosilanes **2d**, **2e**, and **2f** are generated with 98, 96, and 96% deuterium incorporation, respectively (Entries 4 to 7). 3° siloxane undergoes H/D exchange with 98% deuterium incorporation, while poly(methylhydrosiloxane), PMHS, undergoes quantitative deuteration to generate **2g** and **2h**, respectively. Notably, the selectivity toward 1° and 2° hydrosilanes compliments previous investigations into metal-catalyzed H/D exchange; sterically demanding di-*tert*-butylsilane and 3° silanes are not tolerated under our optimized conditions (see the Supporting Information).

**Table 2:**
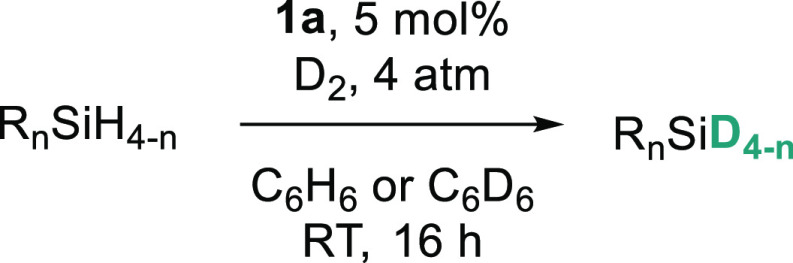

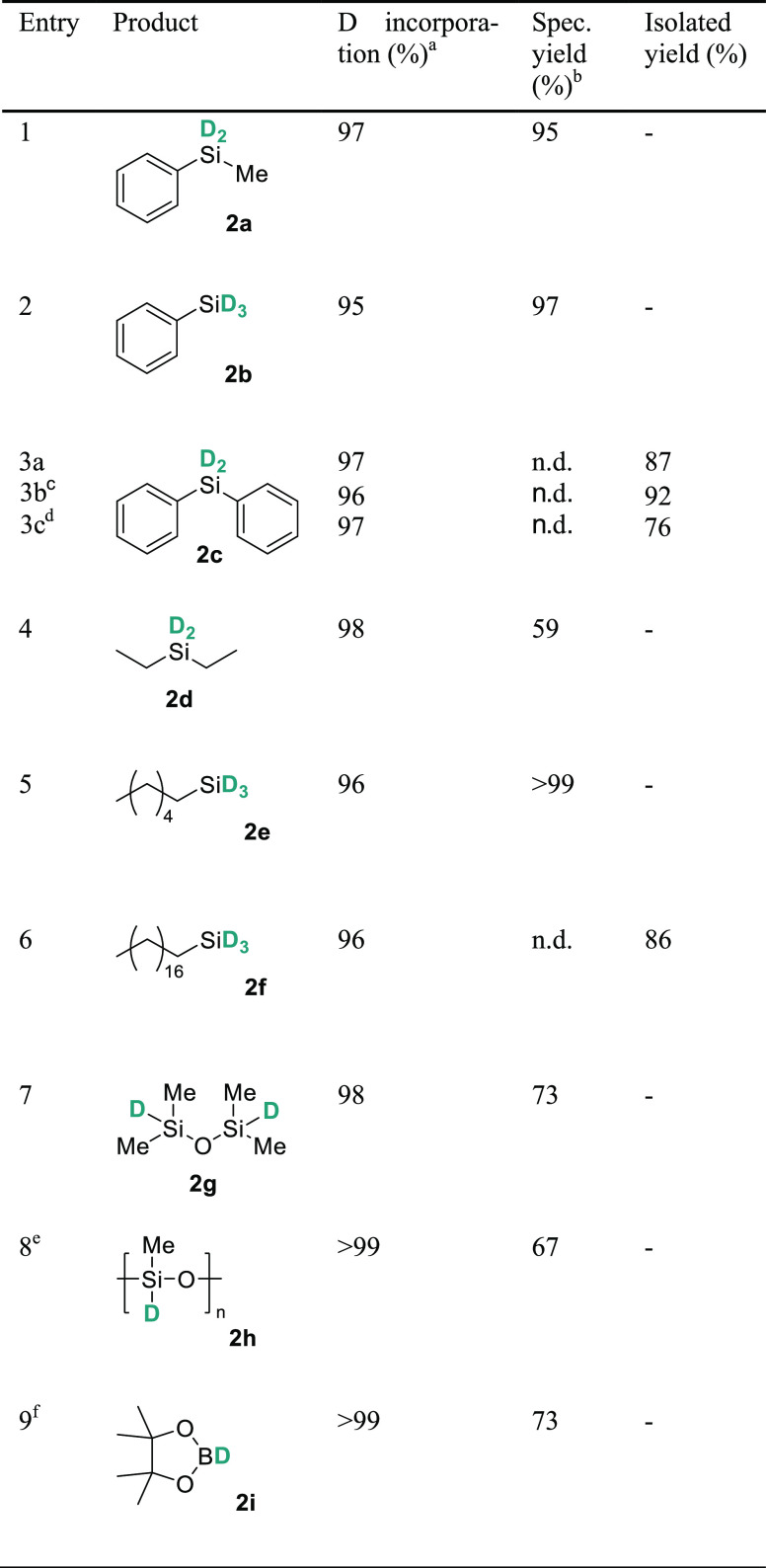
Substrate Scope^a^

a Conditions: 60 mL ampoule containing silane (0.25 mmol), catalyst (5 mol %), D_2_ (filled over liquid N_2_, 4 atm), C_6_H_6_ or C_6_D_6_ (0.5 mL), RT, 16 h.

b Determined by ^1^H NMR spectroscopy comparing residual Si–H to C–H.

c Determined by ^1^H NMR spectroscopy using 1,3,5-trimethoxybenzene (0.25 mmol) as the internal standard.

d 2.5 mmol scale, 300 mL ampoule.

e Pentane (0.5 mL) instead of C_6_H_6_.

f Complete loss of the Si–H signal observed by ^1^H NMR spectroscopy, spectroscopic yield determined by ^2^H NMR spectroscopy relative to toluene-d_8_.

g Determined by ^1^H, ^11^B, and^11^B{^1^H} NMR spectroscopy.

In an attempt to expand the scope to include bulky 2° and 3° silanes in our substrate scope, sterically less encumbered **1b** was synthesized (^dmp^BDKFeCH_2_TMS, Figure 1). Screening of multiple crystals reveals a mixture of mono- and dinuclear **1b**. Previously, **1b** had been prepared and isolated as the four-coordinate tetrahedral THF adduct, which had showed decreased reactivity compared to **1a**.^46^

**Figure 1:**
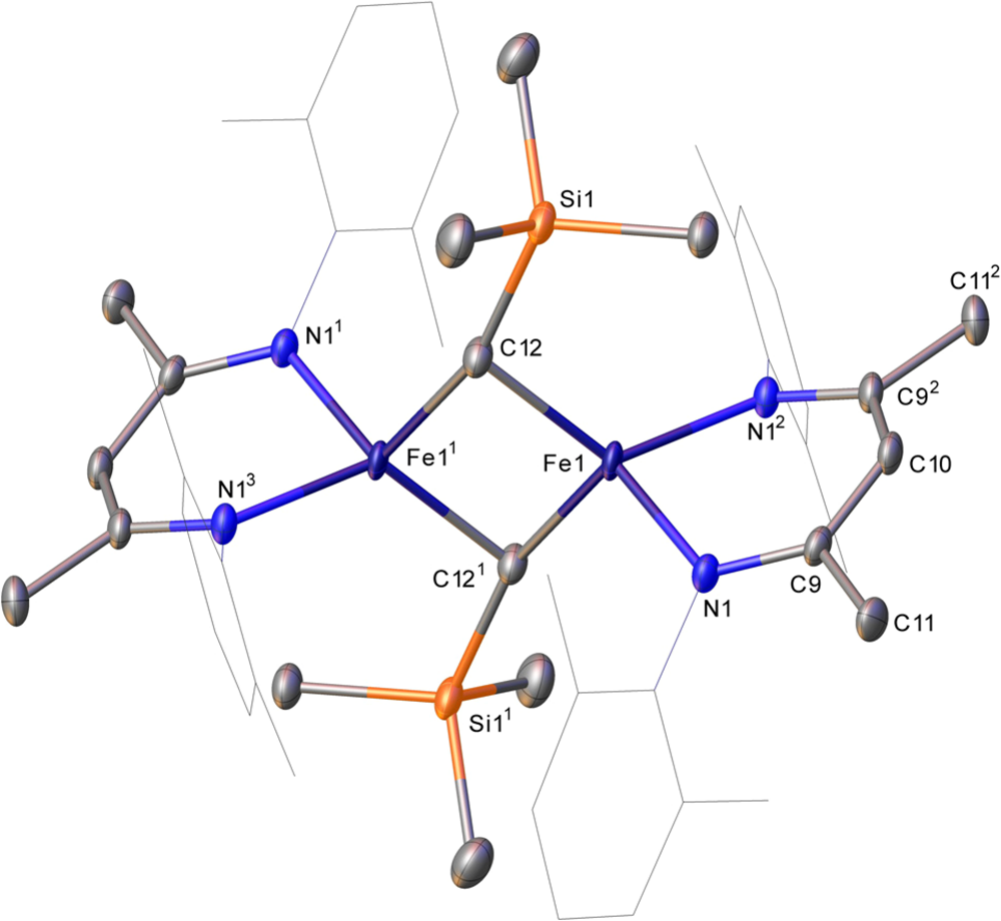
Single-crystal X-ray structure of the dimeric form of **1b**. Hydrogen atoms have been omitted and dmp groups represented in wireframe mode, for clarity. Ellipsoids are represented at 30% probability. Symmetry operations: ^1^ 1 – *x*, 1 – *y*, 1 – *z*; ^2^ 1 – *x*, *y*, *z*; ^3^*x*, 1 – *y*, 1 – *z*.

Employing **1b** as a precatalyst in the H/D exchange of **2b** shows that the level of deuteration is maintained (96% D, 78% spectroscopic yield, compared to 95% D, 97% spectroscopic yield using **1a**). Despite this, **1b** is unable to affect the deuteration of di-*tert*-butylsilane and 3° silanes. Finally, the protocol can be extended to HBpin, with **2i** deuterated quantitively as determined by ^1^H and ^11^B NMR spectroscopy (Table 2, Entry 9). Synthesis of **2i** is notoriously challenging, requiring complex reaction setup^47^ or precious metal catalysts.^40,48−50^ Only two reports of catalytic deuteration of HBpin are known utilizing earth-abundant metal complexes.^51,52^ The H/D exchange of alternative commercially available boranes, catecholborane (HBcat), and 9-borabicyclo(3.3.1)nonane (9-BBN) was also investigated. The reaction of **1a** and **1b** with HBcat yields only partial H/D exchange. However, the reaction of **1a** and **1b** with 9-BBN leads to the formation of a pink solution and no H/D exchange. Performing this reaction stoichiometrically with two equivalents of 9-BBN yields complexes **3a** and **3b**, respectively. Single crystal X-ray diffraction reveals the formation of bridging hydride complexes in the solid state (Scheme 2). It is plausible that complexes **3a** and **3b** arise from the sequestration of hydridic iron species.

**Scheme 2:**
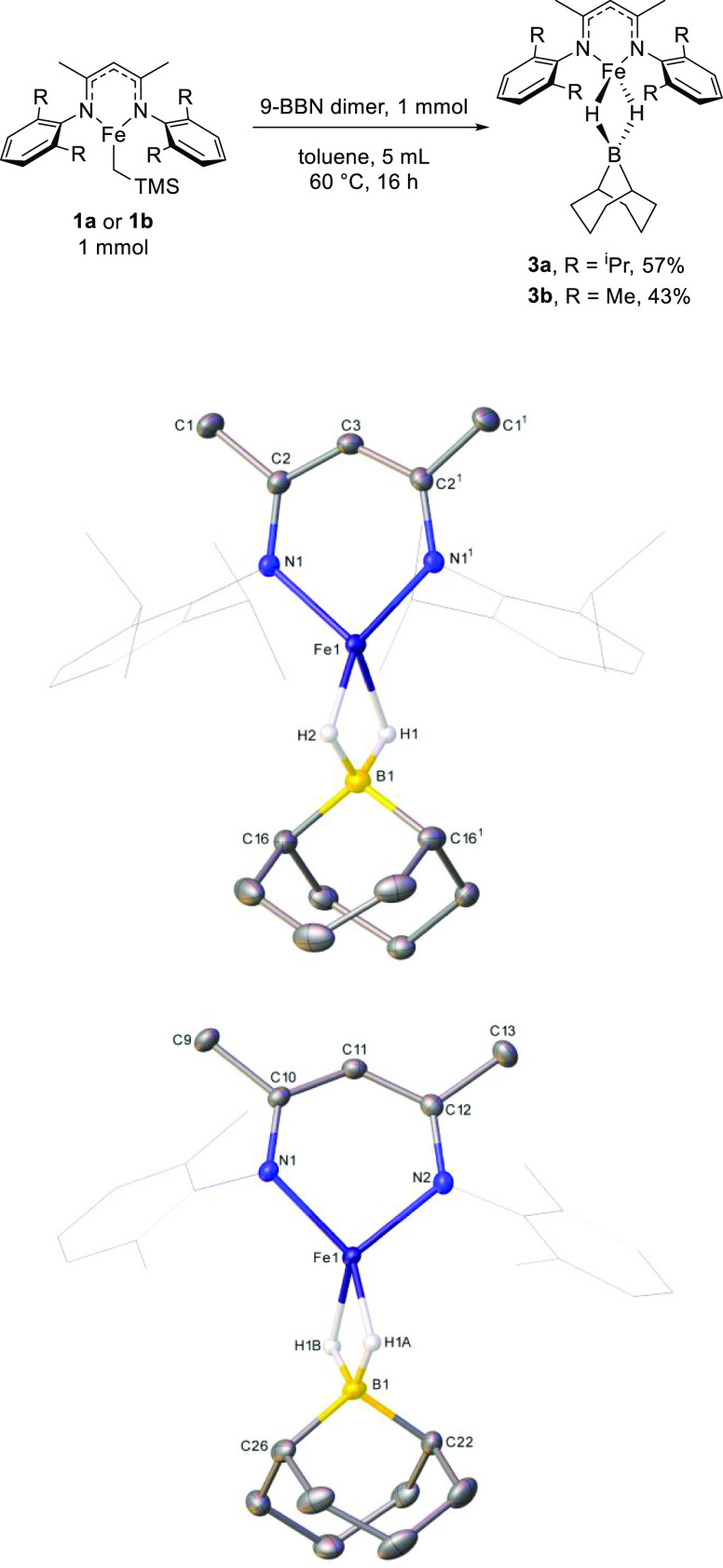
Reaction of Complexes **1a** and **1b** with 9-BBN Result in the Formation of Bridged Hydride Complexes **3a** and **3b**, Respectively Hydrogen atoms (with the exception of those bonded to boron centers) are omitted while dmp and dipp groups are shown in wireframe mode, for clarity. Ellipsoids are represented at 30% probability. Symmetry operation (**3a**): 1 *x*, 1/2 – *y*, *z*

Our efforts then turned to elucidating the mechanism for the iron-catalyzed silane H/D exchange. First, the reversibility of the reaction was examined (Scheme 3a). Under 4 atm of H_2_, **2a** is converted to **2a-H**, indicating that the reaction is highly reversible and dependent on excess D_2_ in the system. The stoichiometric reaction between phenylsilane and **1a** or **1b** reveals no change in the ^1^H NMR spectrum. However, under catalytic conditions with a large excess of silane, reactivity is observed. In situ ^1^H NMR reaction monitoring experiments using **1a** or **1b** both show rapid consumption of PhSiH_3_ between 0.0 and 0.5 h (P1). The rate of PhSiH_3_ consumption then reduces (P2), before accelerating again until the reaction reaches completion (P3). These rate trends are reproducible, and the process is accompanied by the introduction of a peak at 0.04 ppm, assigned to PhSiH_2_(CH_2_TMS), **A**.^53,54^ This suggests a reaction between **1a** or **1b**, and PhSiH_3_ occurs during P1 to generate an iron-hydride intermediate. Previous work has shown that **1a** does not react with H_2_ at 20 atm, indicating that D_2_ is not involved in the initial catalyst activation and further supporting the assertion that precatalyst activation involves reaction with silane.^55^ Furthermore, isolable complexes **3a** and **3b** provide evidence for the cleavage of the precatalyst Fe–C bond with a hydride source and the generation of an iron-hydride intermediate. The production of PhSiH_2_(CH_2_TMS) gives an approximation of the percentage catalyst activation (Scheme 3b, bottom). Interestingly, only partial activation of complex **1a** occurs, reaching 20% after 16 h. Conversely, complex **1b** activates to a greater extent, reaching 80% after 16 h. We postulate that the reduced steric bulk of the 2,6-dimethylphenyl flanking groups within complex **1b** facilitates the reaction with PhSiH_3_. This improved activation is mirrored by an increase in the rate of H/D exchange for **1b** over **1a**, reaching 25 and 9% H/D exchange after 14 h, respectively. A lag phase follows (P2). Holland and co-workers have shown previously that [(BDK)FeH]_2_ complexes readily exchange with D_2_ to form [(BDK)Fe(D/H)]_2_.^56^ We suggest that P2 arises from dimerization and H/D exchange at the iron center. The synthesis of complex **4a** and use in a standard deuteration reaction (Scheme 3c)^45,56^ show 97% deuterium incorporation. This indicates that complex **4a** is an active species in catalysis. Reaction monitoring experiments were undertaken using **4a** as the catalyst. Importantly, no peak at 0.04 ppm is observed, and the reaction proceeds rapidly with no activation or lag phase (P1 and P2 are not observed). Furthermore, the rate of reaction is significantly improved when catalyzed by **4a** instead of **1a**, reaching 20 and 10% H/D exchange after 16 h, respectively [Scheme 3b, top (for full reaction profile, see the Supporting Information, Figure S1)]. The concentration of **4a** was varied to determine the order in catalyst (see the Supporting Information, Figures S10 and S12). The reaction is predicted to be 0.5th order with respect to **4a**.^57^ This indicates that the monomeric form of **4a** participates in the rate-determining step, requiring the dissociation of the [(BDK)Fe(D/H)]_2_ dimer. Monitoring the reaction between **4a** and PhSiH_3_ at various temperatures generates an Eyring plot with Δ*S^‡^* = −164 ± 28.2 J mol^–1^ K^–1^ (see the Supporting Information, Figure S7). We therefore hypothesize that the reaction proceeds via a highly ordered four-membered transition state, in a σ-bond metathesis-type process.

**Scheme 3:**
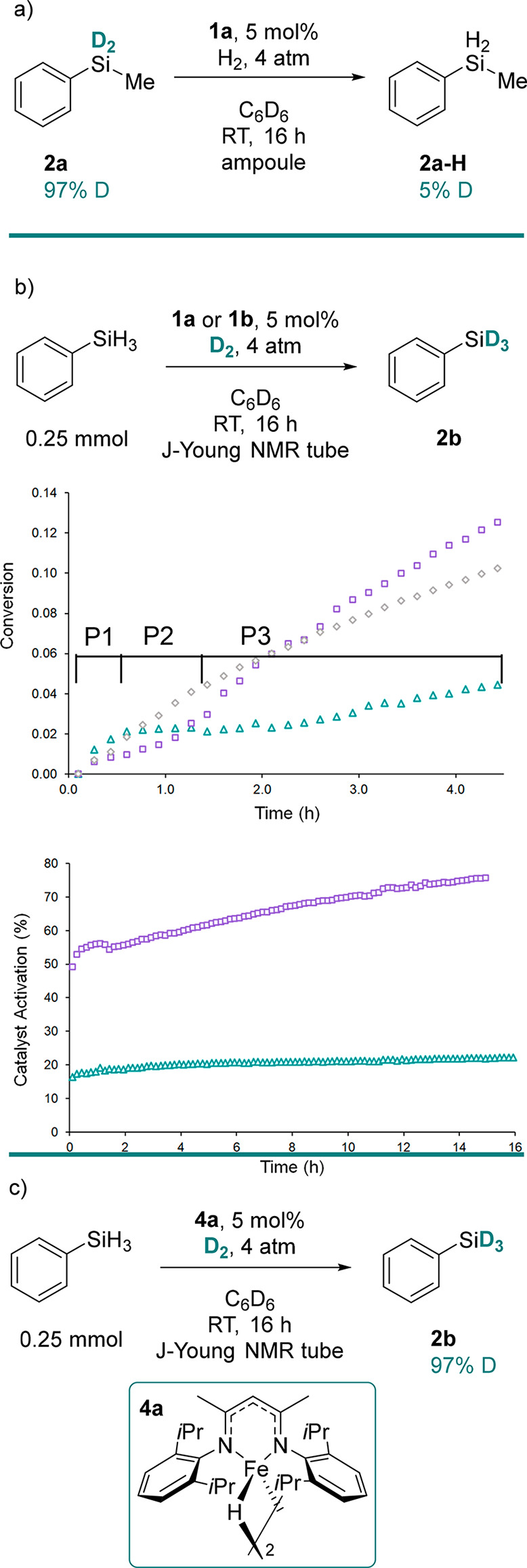
**(**a) Deuterium Incorporation Is Reversible with a Hydrosilane Formed from a Deuterosilane. (b) Reaction Monitoring Shows That There Are Three Distinct Steps When **1a** (triangle) and **1b** (square) Are Employed As Precatalysts, Whereas the Use of Iron Hydride Dimer **4a** (diamond) Shows a Steady Increase in the Product (Top Chart, Measured As a Fraction of 0.25 mmol), **1b** Undergoes Higher Levels of Activation To Form the Corresponding Dimer (**4b**, See the Supporting Information) Compared to **1a** (Bottom Chart). (c) **4a** Gives Near-Quantitative H/D Exchange

Next, we studied the ease of catalyst activation and H/D exchange using density functional theory (DFT) (Figure 2, see the Supporting Information for full computational details). **1b** was chosen as the model precatalyst. The transformations were modeled on the quintet and triplet energy surface to investigate any possible spin-crossover pathway. The triplet surface was calculated to be much higher in energy and is therefore not discussed further here (see the Supporting Information for details).^58^ The activation of **^5^C1** with PhSiH_3_ proceeds with an appreciable barrier of +18.5 kcal mol^–1^ (**^5^TS1**) via an associative σ-bond metathesis reaction. **^5^C2** is generated with a small thermodynamic gain of −0.2 kcal mol^–1^. The large barrier to catalyst activation, accompanied by a small thermodynamic driving force, agrees with the slow activation and lack of stoichiometric reactivity observed experimentally for **1a** and **1b**. Therefore, it is unsurprising that excess PhSiH_3_ is required for reactivity to be observed. Next, using the experimentally obtained Eyring data as a foundation, the H/D exchange of PhSiH_3_ was investigated based on the highly ordered transition state, **^5^TS2**. H/D exchange occurs with a barrier of +10.3 kcal mol^–1^. The reduced barrier of **^5^TS2** compared to **^5^TS1** agrees with the experimentally observed increased rate of reaction for **4a**, compared to **1a** (Scheme 3b). Sequential deuteration steps to access phenyl(silane-d_2_) and phenyl(silane-d_3_) proceed with negligible thermodynamic gain. This indicates that any secondary isotope effect is insignificant (for the full energy-level diagram, see the Supporting Information, Figure S74). These findings suggest that under catalytic conditions, reversible precatalyst activation competes with a fast H/D exchange process.

**Figure 2:**
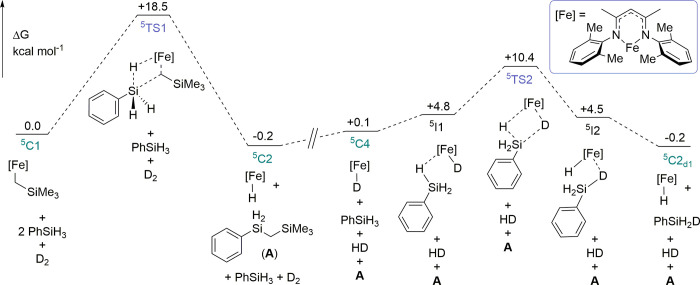
DFT-derived free energy profile for catalyst activation and H/D exchange. Energies are calculated at the B3PW91-D3BJ/Def2-TZVP/IEF-PCM(C_6_H_6_)//BP86/**BS1-**level of theory. All energies are reported in kcal mol^–1^ and referenced to **^5^C1** and reactants.

Based on our mechanistic findings, we propose the following mechanism (Scheme 4). Slow reaction of **C1** with silane generates **C2** via sequential σ-bond metathesis and dimerization. **C2** can activate D_2_, generating **C3**.^56^ Dissociation of **C3** to monomeric **C4** facilitates the silane H/D exchange, regenerating **C2** after dimerization. The partially deuterated silane may re-enter the catalytic cycle until complete deuteration is achieved.

**Scheme 4:**
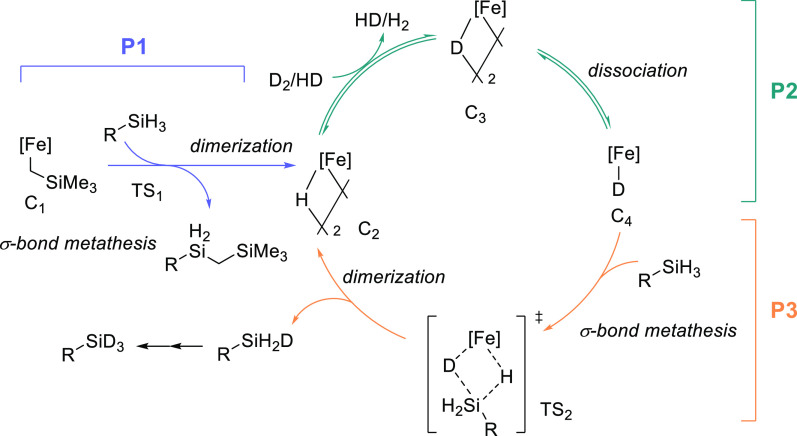
Postulated Catalytic Cycle Based on Experimental and Computational Results

We have reported the first iron-catalyzed H/D exchange of hydrosilanes and hydrosiloxanes. Excellent deuterium incorporation was observed for a variety of 1° and 2° silanes and 3° siloxanes. The conditions also tolerated HBpin, reaching quantitative deuterium incorporation. Mechanistic studies revealed that activity could be enhanced with the replacement of 2,6-diisopropyl (**1a**) flanking groups with 2,6-dimethyl (**1b**). Iron-hydride complex **4a** is suggested to be the active catalyst in the system. The order in the catalyst and Eyring analysis suggest that the dissociation of hydride dimer **4a** is required to facilitate silane H/D exchange. DFT studies reveal a slow and reversible catalyst activation competes with silane deuteration.

## Supporting Information Available

Synthetic methods, analysis, and computational data (PDF)
Crystallographic information (CIF)
Crystallographic information (CIF)

## Supplementary Material

cs2c00224_si_001.pdf

cs2c00224_si_002.cif

cs2c00224_si_003.cif
