# Supplementary material for: Iron-Catalyzed H/D Exchange of Primary Silanes, Secondary Silanes, and Tertiary Siloxanes
Source: ACS Catal. 2022 Feb 18;12(5):2979–85. doi: 10.1021/acscatal.2c00224 (PMC9007460; doi:10.1021/acscatal.2c00224)
Supplement: Supplementary file 1 — cs2c00224_si_001.pdf [file cs2c00224_si_001.pdf]

<u>Supporting Information</u>

# Iron-Catalyzed H/D Exchange of Primary Silanes, Secondary Silanes and Tertiary Siloxanes

**T. G. Linford-Wood, M. F. Mahon, M. N. Grayson* and R. L. Webster***

rw498@bath.ac.uk mng25@bath.ac.uk

Department of Chemistry, University of Bath, Claverton Down, Bath, United Kingdom, BA2 7AY

## Contents

**1 General Considerations 1**

**2 General Method for Deuteration of Silanes 1**

**3 Substrate Scope Spectroscopic Data 2**

**4 Catalyst and Reagent Synthesis 6**

**5 Crystallographic Data 9**

**6 Method for Hydrogenation of Methylphenyl(silane-d$_2$) 14**

**7 Method for Deuterium Labelling of Propylbenzene 14**

**8 Reaction Monitoring Experiments 15**

8.1 General Method . . . . . 15

8.2 Reaction Monitoring Plots . . . . . 15

**9 Substrate Synthesis 22**

**10 Substrate Scope Spectra 24**

**11 Complex and Reagent Spectra 45**

**12 Synthesised Substrate Spectra 47**

**13 Computational Detail 53**

13.1 Computational Method . . . . . 53

13.2 DFT-Derived Mechanism . . . . . 54

13.3 Atomic Coordinates . . . . . 55

13.3.1 Catalyst Activation . . . . . 55

13.3.2 H/D Exchange . . . . . 77

**14 References** **86**

# 1 General Considerations

Reagents were purchased from Fisher Scientific or Merck and dried and distilled prior to use. Laboratory grade pentane was purchased from Fisher Scientific and used without further purification. THF, $C_6H_6$ and $C_6D_6$ were dried over Na/benzophenone and distilled prior to use. DCM was dried over $CaH_2$ and distilled prior to use. Pre-catalyst **1a** was synthesised following literature procedure.[1] $^{dipp}$BDKFe($\mu$-Cl)$_2$Li(THF)$_2$ was synthesised following literature procedure.[2] NMR data was collected at 300, 400 or 500 MHz on Agilent or Bruker instruments in $C_6D_6$, $C_6H_6$ or $CDCl_3$ at 298 K and referenced to the residual solvent peak. Reactions were undertaken using standard glovebox (Ar, 0.1 ppm $H_2O$ and 0.1 ppm $O_2$) and Schlenk line ($N_2$) techniques unless otherwise stated. All reactions were undertaken in 60 mL Teflon-sealed J-young ampoules unless otherwise stated. $D_2$ cylinder was purchased from BOC.

Crystallographic data for **1b**, **3a**, **3b**, **[$^{dmp}$BDKFeH]$_2$** and **Fe($^{dmp}$BDK)$_2$** were collected on an Agilent SuperNova diffractometer (using a Cu-K$\alpha$ radiation). All experiments were conducted at 150 K, solved using SHELXS and refined using SHELXL via the Olex2 interface.[3-5] Crystallographic data for all compounds have been deposited with the Cambridge Crystallographic Data Centre and supplementary publications CCDC 2128834-2128837 and 2141291 for **1b**, **3a**, **3b**, **[$^{dmp}$BDKFeH]$_2$** and **Fe($^{dmp}$BDK)$_2$**, respectively. Copies of these data can be obtained free of charge on application to CCDC, 12 Union Road, Cambridge, CB2 1EZ, UK [fax(+44) 1223 336033, e-mail: deposit@ccdc.cam.ac.uk].

# 2 General Method for Deuteration of Silanes

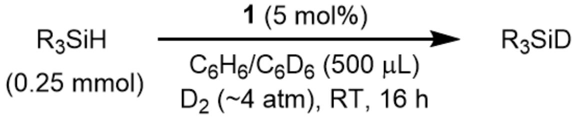


All reactions were performed in duplicate in protonated and deuterated solvent. To a flame-dried 60 mL Teflon-sealed J-young ampoule (unless otherwise stated) containing pre-catalyst **1a** (5 mol%), $C_6H_6$ or $C_6D_6$ (500 $\mu$L) and silane (0.25 mmol) were added. The vessel was sealed, removed from the glovebox and subjected to freeze-pump-thaw cycles until a continuous vacuum was achieved. The ampoule was cooled in liquid nitrogen and backfilled with $D_2$ gas. The mixture was warmed to room temperature and stirred for 16 hours, turning from yellow to dark yellow after 30 minutes. Reactions containing volatile silanes in deuterated solvent were transferred to a H-distillation tube and separated from **1a** by vacuum distillation. 1,3,5-Trimethoxybenzene (0.25 mmol) was added as a stock solution and spectroscopic yield and D-incorporation were determined by $^1$H NMR spectroscopy. Reactions containing non-volatile silanes in deuterated solvent were isolated by FCC ($SiO_2$, pentane). Reactions in protonated solvent were transferred to the glovebox, toluene-d$_8$ (26.6 $\mu$L, 0.25 mmol) was added and D-incorporation was determined by $^2$H NMR spectroscopy.

## 3 Substrate Scope Spectroscopic Data

**Methylphenyl(silane-d$_2$), 2a**

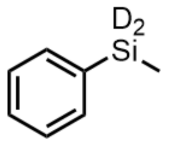


Deuterium incorporation: 97%

Spectroscopic yield: 95%

**$^1$H NMR** ($C_6D_6$, 400 MHz) $\delta$ 7.47-7.44 (m, 2H, $^m$ArH), 7.16-7.12 (m, 3H, $^{o,p}$ArH), 4.49-4.46 (m, 0.1H, SiH), 0.18 (s, 3H, $CH_3$); **$^{13}$C{$^1$H} NMR** ($C_6D_6$, 100 MHz) $\delta$ 135.2 ($^{Ar}$C), 133.4 ($^{Ar}$C), 129.8 ($^{Ar}$C), -7.81 ($CH_3$); **$^{29}$Si NMR** ($C_6D_6$, 125 MHz) $\delta$ -36.7 (p, $J$=29.6 Hz); **$^2$H NMR** ($C_6H_6$, 77 MHz) $\delta$ 4.58. NMR data consistent with a sample of the commercially available protonated starting material.

**Phenyl(silane-d$_3$), 2b**

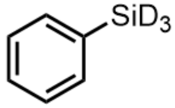


**Catalysed by 1a**

Deuterium incorporation: 95%

Spectroscopic yield: 97%

**$^1$H NMR** ($C_6D_6$, 400 MHz) $\delta$ 7.40-7.37 (m, 2H, $^m$ArH), 7.12-7.05 (m, 3H, $^{o,p}$ArH), 4.21 (s(br), 0.15H, SiH); **$^{13}$C{$^1$H} NMR** ($C_6D_6$, 100 MHz) $\delta$ 136.1 ($^{Ar}$C), 130.0 ($^{Ar}$C), 128.4 ($^{Ar}$C); **$^{29}$Si NMR** ($C_6D_6$, 125 MHz) $\delta$ -61.0 (hept, $J$=30.5 Hz); **$^2$H NMR** ($C_6H_6$, 77 MHz) $\delta$ 4.29. NMR data consistent with a sample of the commercially available protonated starting material.

**Catalysed by 1b**

Deuterium incorporation: 96%

Spectroscopic yield: 78%

**$^1$H NMR** ($C_6D_6$, 400 MHz) $\delta$ 7.40-7.38 (m, 2H, $^m$ArH), 7.14-7.05 (m, 3H, $^{o,p}$ArH), 4.21 (s(br), 0.11H, SiH); **$^2$H NMR** ($C_6H_6$, 77 MHz) $\delta$ 4.26. NMR data consistent with a sample of the commercially available protonated starting material.

**Diphenyl(silane-d$_2$), 2c**

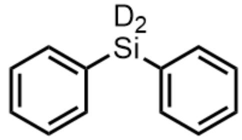


**0.25 mmol scale**

Deuterium incorporation: 97%

Isolated yield: 87%

Isolated as a colourless oil (40.3 mg, 0.217 mmol, 87%). **$^1$H NMR** ($C_6D_6$, 400 MHz) $\delta$ 7.55-7.53 (m, 4H, $^m$ArH), 7.19-7.13 (m, 6H, $^{o,p}$ArH), 5.10 (s(br), 0.06H, SiH); **$^{13}$C{$^1$H} NMR** ($C_6D_6$, 125 MHz) $\delta$ 136.1, 131.7, 130.1, 128.5; **$^{29}$Si NMR** ($C_6D_6$, 125 MHz) $\delta$ -34.3 (p, $J$=30.3 Hz); **$^2$H NMR** ($C_6H_6$, 77 MHz) $\delta$ 5.10. NMR data consistent with a sample of the commercially available protonated starting material.

**2.5 mmol scale**

Deuterium incorporation: 96%

Isolated yield: 92%

Reaction undertaken in a 375 mL ampoule. Isolated as a colourless oil (430 mg, 2.31 mmol, 92%). **$^1$H NMR** ($C_6D_6$, 400 MHz) $\delta$ 7.55-7.53 (m, 4H, $^m$ArH), 7.19-7.12 (m, 6H, $^{o,p}$ArH), 5.11 (s(br), 0.08H, SiH); **$^{13}$C{$^1$H} NMR** ($C_6D_6$, 125 MHz) $\delta$ 136.1, 131.7, 130.1, 128.5; **$^{29}$Si NMR** ($C_6D_6$, 125 MHz) $\delta$ -34.3 (p, $J$=30.3 Hz); **$^2$H NMR** ($C_6H_6$, 77 MHz) $\delta$ 5.09. NMR data consistent with a sample of the commercially available protonated starting material.

**0.25 mmol in Pentane**

Deuterium incorporation: 97%

Isolated yield: 76%

Isolated as a colourless oil (35.5 mg, 0.190 mmol, 76%). **$^1$H NMR** ($C_6D_6$, 400 MHz) $\delta$ 7.55-7.53 (m, 4H, $^m$ArH), 7.19-7.12 (m, 6H, $^{o,p}$ArH), 5.11 (s(br), 0.07H, SiH); NMR data consistent with a sample of the commercially available protonated starting material.

**Diethyl(silane-$d_2$), 2d**

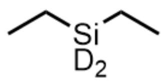


Deuterium incorporation: 98%

Spectroscopic yield: 59%

**$^1$H NMR** ($C_6D_6$, 400 MHz) $\delta$ 3.84 (s(br), 0.04H, SiH), 0.95 (t, $J$=7.9 Hz, 6H), 0.52 (q, $J$=8.0 Hz, 4H); **$^{13}$C{$^1$H} NMR** ($C_6D_6$, 125 MHz) $\delta$ 9.2, 1.1; **$^{29}$Si NMR** ($C_6D_6$, 125 MHz) $\delta$ -23.7 (p, $J$=28.0 Hz); **$^2$H NMR** ($C_6H_6$, 77 MHz) $\delta$ 3.89. NMR data consistent with a sample of the commercially available protonated starting material.

**Hexyl(silane-$d_3$), 2e**

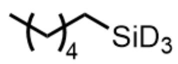


Deuterium incorporation: 96%

Spectroscopic yield: >99%

**$^1$H NMR** ($C_6D_6$, 400 MHz) $\delta$ 3.64-3.61 (m, 0.13H, SiH), 1.32-1.10(m, 8H), 0.87 (t, $J$=7.0 Hz, 3H, $CH_3$), 0.51 (t, $J$=7.7 Hz, 2H, $SiCH_2$); **$^{13}$C{$^1$H} NMR** ($C_6D_6$, 125 MHz) $\delta$ 32.5, 31.8, 26.6, 22.9, 14.3, 5.9; **$^{29}$Si NMR** ($C_6D_6$, 125 MHz) $\delta$ -60.6 (hept, $J$=29.3 Hz); **$^2$H NMR** ($C_6H_6$, 77 MHz) $\delta$ 3.67. NMR data consistent with a sample of the commercially available protonated starting material.

**Octadecyl(silane-d$_3$), 2f**

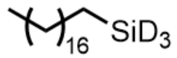

Deuterium incorporation: 96%

Isolated yield: 86%

Isolated as a colourless solid (61.8 mg, 0.215 mmol, 86%). **$^1$H NMR** ($C_6D_6$, 400 MHz) $\delta$ 3.64 (s(br), 0.12H, SiH), 1.34-1.25 (m, 32H), 0.93-0.88 (m, 3H, $CH_3$), 0.56 (t, $J$=7.0 Hz, 2H, $SiCH_2$); **$^{13}$C{$^1$H} NMR** ($C_6D_6$, 125 MHz) $\delta$ 33.0, 32.4, 30.3, 30.2, 30.2, 30.0, 29.9, 29.7, 26.7, 23.2, 14.4, 6.0, 1.4; **$^{29}$Si NMR** ($C_6D_6$, 125 MHz) $\delta$ -60.8 (hept, $J$=29.3 Hz); **$^2$H NMR** ($C_6H_6$, 77 MHz) $\delta$ 3.64. NMR data consistent with a sample of the commercially available protonated starting material.

**1,1,3,3-Tetramethyldi(siloxane-d), 2g**

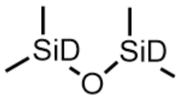

Deuterium incorporation: 98%

Spectroscopic yield: 73%

**$^1$H NMR** ($C_6D_6$, 400 MHz) $\delta$ 4.97-4.94 (m, 0.05H, SiH), 0.13 (s, 12H, $CH_3$); **$^{13}$C{$^1$H} NMR** ($C_6D_6$, 125 MHz) $\delta$ 0.55 (m); **$^{29}$Si NMR** ($C_6D_6$, 125 MHz) $\delta$ -4.94 (t, $J$=31.4 Hz); **$^2$H NMR** ($C_6H_6$, 77 MHz) $\delta$ 5.00. NMR data consistent with a sample of the commercially available protonated starting material.

**Poly(methyldeuterosiloxane), 2h**

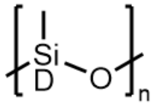

Deuterium incorporation: quant.

Spectroscopic yield: 67% (based on $^2$H NMR)

Analysed with no further purification. **$^1$H NMR** ($C_6D_6$, 400 MHz) $\delta$ 0.27 (s(br), 3H); **$^2$H NMR** ($C_6H_6$, 77 MHz) $\delta$ 5.14. NMR data consistent with a sample of the commercially available protonated starting material.

**4,4,5,5-Tetramethyl-1,3,2-dioxa(borolane-d), 2i**

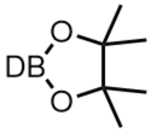

Deuterium incorporation: quant.

Spectroscopic yield: 73%

**$^1$H NMR** ($C_6D_6$, 400 MHz) $\delta$ 1.00 (s, 12H); **$^{13}$C{$^1$H} NMR** ($C_6D_6$, 125 MHz) $\delta$ 83.1, 24.9; **$^{11}$B NMR** ($C_6D_6$, 128 MHz) $\delta$ 28.3 (s). NMR data consistent with a sample of the commercially available protonated starting material.

## Failed Substrates

**Triphenylsilane**

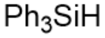


Deuterium incorporation: 0%

**Di-*tert*-butylsilane**

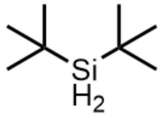


Deuterium incorporation: 0%

**Dimethylphenylsilane**

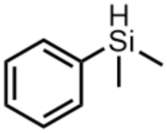


Deuterium incorporation: 18%

**(4-(Chloromethyl)phenyl)dimethylsilane**

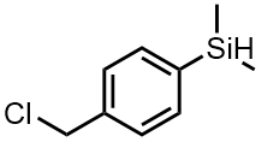


Deuterium incorporation: 0%

**Dimethyl(4-vinylphenyl)silane**

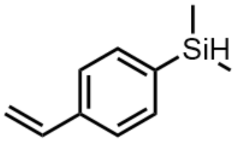


Deuterium incorporation: 0%

**Triethylsilane**

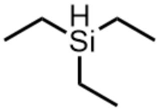

Deuterium incorporation: 12%

**(3-Mercaptopropyl)methyldimethoxysilane**

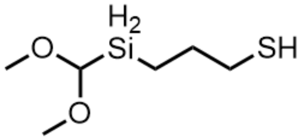


Deuterium incorporation: 0%

**Triethoxysilane**

$(EtO)_3SiH$

Deuterium incorporation: 0%

# 4 Catalyst and Reagent Synthesis

**$^{2,6\text{-dimethylphenyl}}\beta$-Diketiminate**

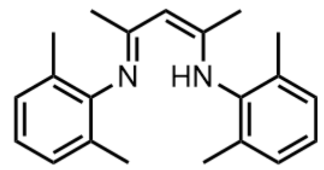


Title compound was synthesised by modified literature procedure.[6] To a 20 mL microwave reactor vial, 2,6-dimethylaniline (6.05 g, 50 mmol, 2.0 equiv.), acetylacetone (2.50 g, 25 mmol, 1.0 equiv.) and conc. HCl (2.5 mL) were added. The vial was sealed and heated to 85 °C under 150 W microwave irradiation with stirring for 30 minutes. The mixture was cooled to room temperature and a solution of $Na_2CO_3$ (4.00 g) in water (100 mL) was added. The mixture was extracted with DCM (3 × 100 mL). The organic extracts were combined, dried over $MgSO_4$, filtered and concentrated under reduced pressure. The crude product was dissolved in methanol (100 mL) and cooled to -20 °C, yielding white crystals of $^{2,6\text{-dimethylphenyl}}\beta$-diketiminate (3.59 g, 11.7 mmol, 47%). **$^1$H NMR** ($CDCl_3$, 400 MHz) $\delta$ 12.21 (s (br), 1H, NH), 7.05 (d, $J$=7.5 Hz, 4H, $^m$ArH), 6.96 (dd, $J$=8.1, 6.8 Hz, 2H, $^p$ArH), 4.90 (s, 1H, CH), 2.18 (s, 12H, $CH_3$), 1.71 (s, 6H, $CH_3$); **$^{13}$C{$^1$H} NMR** ($CDCl_3$, 125 MHz) $\delta$ 160.9, 143.9, 132.3, 127.9, 124.4, 93.5, 20.5, 18.5; **HRMS**(ESI) predicted: 307.2174, found: 307.2176 $[M+H]^+$.

**$^{2,6\text{-dmp}}$BDKFe($CH_2$TMS), 1b**

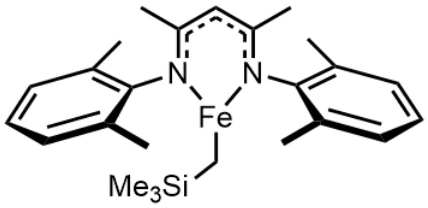

Title compound was synthesised by modified literature procedure.[1] To a flame dried schlenk containing a solution of $^{2,6\text{-dmp}}$BDK (2.03 g, 6.62 mmol, 1.0 equiv.) in THF (40 mL) at -78 °C, $^{n}$butyllithium (2.5M solution in hexanes, 1.6 mL, 6.62 mmol, 1.0 equiv.) was added dropwise. The solution was warmed to room temperature and stirred for 1 hour. $FeCl_2{\cdot}THF_{1.5}$ (1.56 g, 6.62 mmol, 1.0 equiv.) was added and the mixture was stirred for a further 45 minutes. $LiCH_2TMS$ (623 mg, 6.62 mmol, 1.0 equiv.) was added and the mixture was stirred for a further 45 minutes. Volatiles were removed under reduced pressure and the remaining yellow solid was left under vacuum ($2 \times 10^{-2}$ mbar) for 2 hours to remove residual coordinating THF. The mixture was suspended in pentane ($2 \times 5$ mL) and concentrated to remove residual coordinating THF. Pentane (20 mL) was added and the solution isolated by cannula filtration. The filtrate was concentrated to 5 mL and cooled to -20 °C, yielding red crystals of $^{2,6\text{-dimethylphenyl}}$BDKFe($CH_2$TMS) (2.50 g, 5.57 mmol, 84%). **$^{1}$H NMR** ($C_6D_6$, 400 MHz) $\delta$ 98.09 (s(br), 1H, $^{backbone}$CH), 78.86 (s(br), 6H, $^{backbone}CH_3$), 34.41 (s(br), 9H, $SiMe_3$), -4.58 (s(br), 4H, $^{Ar}$CH), -60.28 (s(br), 12H, $^{Ar}CH_3$), -68.73 (s(br), 2H, $^{Ar}$CH). **CHN** calculated for $C_{25}H_{36}FeN_2Si$: C, 66.95; H, 8.09; N, 6.25; found: C, 66.89; H, 8.22; N, 6.08. **MP** 79-81 °C. **UV/vis** (pentane) 321 nm. **XRD** relevant crystallographic data are reported in the X-ray crystallography section. Screening of multiple crystals revealed a mixture of monomeric and dimeric $^{2,6\text{-dmp}}$BDKFe($CH_2$TMS).

**$^{2,6\text{-dipp}}$BDKFe-($\mu$-H)$_2$-9-BBN, 3a**

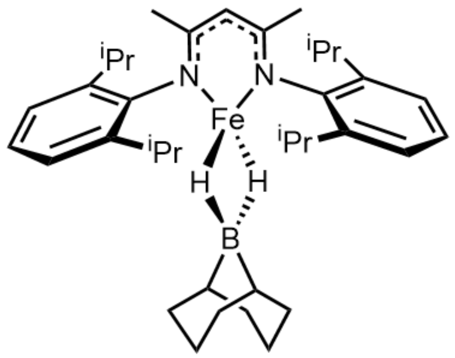


To an ampoule containing **1a** (561 mg, 1.0 mmol, 1.0 equiv.) dissolved in toluene (5 mL), 9-borabicyclo(3.3.1)nonane (244 mg, 1.0 mmol, 1.0 equiv.) was added. The vessel was heated to 60 °C and stirred for 16 hours. The solution was concentrated under reduced pressure. The solid was dissolved in a minimum amount of pentane and cooled to -20 °C, yielding pink crystals of $^{2,6\text{-dipp}}$BDKFe-($\mu$-H)$_2$-9-BBN (337 mg, 0.565 mmol, 57%). **$^{1}$H NMR** ($CDCl_3$, 400 MHz) $\delta$ 161.88, 83.25, 57.93, 41.76, 37.95, 29.76, 19.08, -2.60, -10.19, -32.75, -35.03, -50.31. **CHN** calculated for $C_{37}H_{57}BFeN_2$: C, 74.50; H, 9.63; N, 4.70; found: C, 74.51; H, 9.44; N, 4.63. **MP** 130-131 (decomposition), 188-190 (melt) °C. **UV/vis** (pentane) 397, 296 nm. **XRD** relevant crystallographic data are reported in the X-ray crystallography section.

**$^{2,6\text{-dipp}}$BDKFe-($\mu$-H)$_2$-9-BBN, 3b**

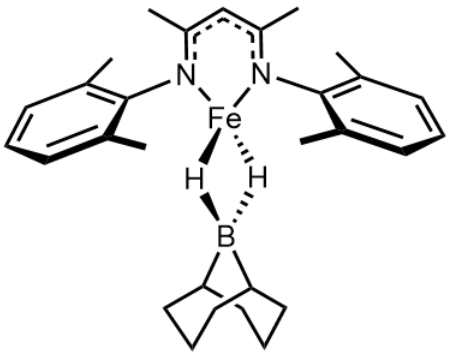


To an ampoule containing **1b** (449 mg, 1.0 mmol, 1.0 equiv.) dissolved in toluene (5 mL), 9-borabicyclo(3.3.1)nonane (244 mg, 1.0 mmol, 1.0 equiv.) was added. The vessel was heated to 60 °C and stirred for 16 hours. The solution was concentrated under reduced pressure. The solid was dissolved in a minimum amount of toluene and cooled to -20 °C, yielding pink crystals of $^{2,6\text{-dmp}}$BDKFe-($\mu$-H)$_2$-9-BBN (210 mg, 0.433 mmol, 43%). **$^1$H NMR** ($CDCl_3$, 400 MHz) $\delta$ 163.25, 83.55, 54.67, 36.53, 31.15, 23.33, 22.80, 7.62, -31.95, -48.75. **CHN** calculated for $C_{29}H_{41}BFeN_2$: C, 71.92; H, 8.53; N, 5.78; found: C, 71.98; H, 8.57; N, 5.74. **MP** 156-158 (decomposition), 208-212 (melt) °C. **UV/vis** (pentane) 393, 293 nm. **XRD** relevant crystallographic data are reported in the X-ray crystallography section.

**[$^{\text{dipp}}$BDKFeH]$_2$, 4a**

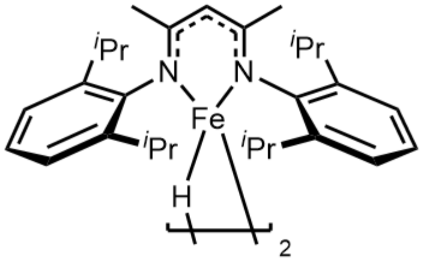


Title compound was synthesised according to literature procedure.[7] To a solution of **1a** (200 mg, 0.36 mmol, 1.0 equiv.) in toluene (1 mL), TMP.$BH_3$ (167 mg, 1.08 mmol, 3.0 equiv.) was added. The vessel was sealed and stirred at 80 °C for 16 hours. The mixture was concentrated under reduced pressure and pentane (5 mL) was added. The mixture was filtered and cooled to -20 °C yielding red crystals of [$^{\text{dipp}}$BDKFeH]$_2$ (63.5 mg, 0.134 mmol, 37%).**$^1$H NMR** ($C_6D_6$, 400 MHz) $\delta$ 13.0 (s(br), 12H, 8H, $CH_3$, $^{m}$ArH), 7.15 (s(br), 24H, $^{i}$Pr$CH_3$), -24.0 (s(br), 4H, $^{p}$ArH), -24.9 (s(br), 24H, $^{i}$Pr$CH_3$), -56.1 (s(br), 2H, $^{i}$PrCH). **XRD** confirmed by unit cell check. Data in agreement with the literature. [7,8]

The synthesis of [$^{\text{dmp}}$BDKFeH]$_2$ was also attempted by an analogous method. A mixture of complexes [$^{\text{dmp}}$BDKFeH]$_2$ and Fe($^{\text{dmp}}$BDK)$_2$ were isolated. The relevant crystallographic data are reported in the X-ray crystallography section. Thus far, a direct and selective synthesis of these complexes has not been achieved.

# 5 Crystallographic Data

## $^{2,6\text{-dmp}}$BDKFe($CH_2$TMS), 1b

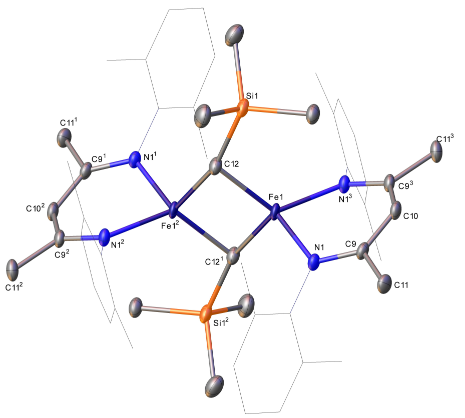

Table S1 Crystal data and structure refinement for **1b**.

| | |
|---|---|
| Identification code | **1b** |
| Empirical formula | $C_{50}H_{72}Fe_2N_4Si_2$ |
| Formula weight | 448.50 |
| Temperature/K | 150.00(10) |
| Crystal system | orthorhombic |
| Space group | Cmce |
| a/Å | 18.7305(7) |
| b/Å | 14.4274(7) |
| c/Å | 17.8945(7) |
| α/° | 90 |
| β/° | 90 |
| γ/° | 90 |
| Volume/Å$^3$ | 4835.7(4) |
| Z | 4 |
| $\rho_{calc}$g/cm$^3$ | 1.232 |
| μ/mm$^{-1}$ | 5.557 |
| F(000) | 1920.0 |
| Crystal size/mm$^3$ | 0.231 × 0.174 × 0.063 |
| Radiation | Cu Kα (λ = 1.54184) |
| 2Θ range for data collection/° | 9.18 to 179.056 |
| Index ranges | -20 ≤ h ≤ 22, -17 ≤ k ≤ 17, -23 ≤ l ≤ 22 |
| Reflections collected | 24539 |
| Independent reflections | 2517 [$R_{int}$ = 0.0368, $R_{sigma}$ = 0.0170] |
| Data/restraints/parameters | 2517/5/163 |
| Goodness-of-fit on F$^2$ | 1.107 |
| Final R indexes [I>=2σ (I)] | $R_1$ = 0.0419, w$R_2$ = 0.1149 |
| Final R indexes [all data] | $R_1$ = 0.0433, w$R_2$ = 0.1161 |
| Largest diff. peak/hole / e Å$^{-3}$ | 0.60/-1.00 |

The asymmetric unit in **1b** comprises one quarter of a molecule, in which atoms Fe1, Si1, C10 and C13 are coincident with crystallographic mirror plane. The proximity of the iron centre to a space-group inversion centre serves to complete the dimer. C12 was seen to be disordered either side of the aforementioned mirror plane. The hydrogen atoms attached to this carbon were located and refined with the inclusion of some distance restraints, to assist convergence.

### $^{2,6\text{-dipp}}$BDKFe-($\mu$-H)$_2$-9-BBN, 3a

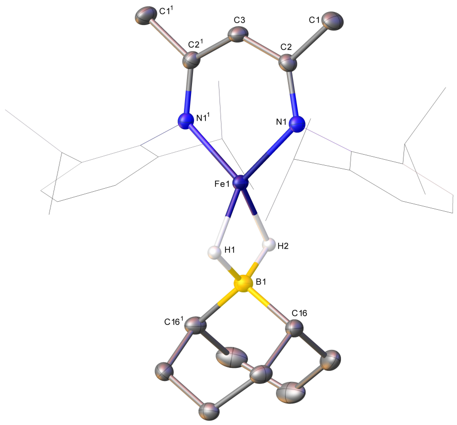


Table S2 Crystal data and structure refinement for **3a**.

| | |
|---|---|
| Identification code | **3a** |
| Empirical formula | $C_{37}H_{57}BFeN_2$ |
| Formula weight | 596.50 |
| Temperature/K | 150.00(10) |
| Crystal system | orthorhombic |
| Space group | Pnma |
| a/Å | 19.2143(4) |
| b/Å | 20.1989(4) |
| c/Å | 9.3011(2) |
| α/° | 90 |
| β/° | 90 |
| γ/° | 90 |
| Volume/Å$^3$ | 3609.83(13) |
| Z | 4 |
| $\rho_{calc}$g/cm$^3$ | 1.098 |
| μ/mm$^{-1}$ | 3.518 |
| F(000) | 1296.0 |
| Crystal size/mm$^3$ | 0.170 × 0.134 × 0.089 |
| Radiation | Cu Kα (λ = 1.54184) |
| 2Θ range for data collection/° | 8.756 to 147.32 |
| Index ranges | -23 ≤ h ≤ 23, -25 ≤ k ≤ 19, -11 ≤ l ≤ 11 |
| Reflections collected | 26996 |
| Independent reflections | 3739 [$R_{int}$ = 0.0594, $R_{sigma}$ = 0.0315] |
| Data/restraints/parameters | 3739/0/204 |
| Goodness-of-fit on F$^2$ | 1.042 |
| Final R indexes [I>=2σ (I)] | $R_1$ = 0.0425, w$R_2$ = 0.1052 |
| Final R indexes [all data] | $R_1$ = 0.0491, w$R_2$ = 0.1091 |
| Largest diff. peak/hole / e Å$^{-3}$ | 0.25/-0.25 |

The asymmetric unit in **3a** comprises half of one molecule. Atoms Fe1, C3, B1, C18 and C20 are coincident with a crystallographic mirror plane which serves to generate the remainder of the complex. H1 and H2 (attached to B1, and also seated on the mirror, were located and refined without restraints).

### $^{2,6\text{-dipp}}$BDKFe-($\mu$-H)$_2$-9-BBN, 3b

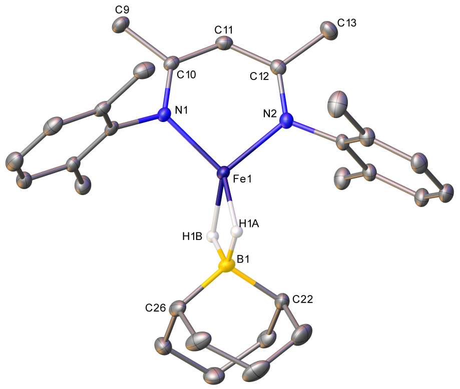


Table S3 Crystal data and structure refinement for **3b.**

| | |
|---|---|
| Identification code | **3b** |
| Empirical formula | $C_{29}H_{41}BFeN_2$ |
| Formula weight | 484.30 |
| Temperature/K | 150.00(10) |
| Crystal system | monoclinic |
| Space group | C2/c |
| a/Å | 18.1664(2) |
| b/Å | 12.4604(2) |
| c/Å | 23.9387(3) |
| α/° | 90 |
| β/° | 94.823(1) |
| γ/° | 90 |
| Volume/Å$^3$ | 5399.59(13) |
| Z | 8 |
| $\rho_{calc}$g/cm$^3$ | 1.191 |
| μ/mm$^{-1}$ | 4.596 |
| F(000) | 2080.0 |
| Crystal size/mm$^3$ | 0.289 × 0.214 × 0.166 |
| Radiation | Cu Kα (λ = 1.54184) |
| 2Θ range for data collection/° | 7.412 to 146.078 |
| Index ranges | -22 ≤ h ≤ 17, -15 ≤ k ≤ 15, -29 ≤ l ≤ 28 |
| Reflections collected | 26805 |
| Independent reflections | 5384 [$R_{int}$ = 0.0360, $R_{sigma}$ = 0.0237] |
| Data/restraints/parameters | 5384/0/312 |
| Goodness-of-fit on F$^2$ | 1.035 |
| Final R indexes [I>=2σ (I)] | $R_1$ = 0.0329, w$R_2$ = 0.0842 |
| Final R indexes [all data] | $R_1$ = 0.0363, w$R_2$ = 0.0865 |
| Largest diff. peak/hole / e Å$^{-3}$ | 0.24/-0.33 |

The boron bound hydrogens were located in the structure of compound **3b** and refined without restraints.

## $[^{dmp}BDKFeH]_2$

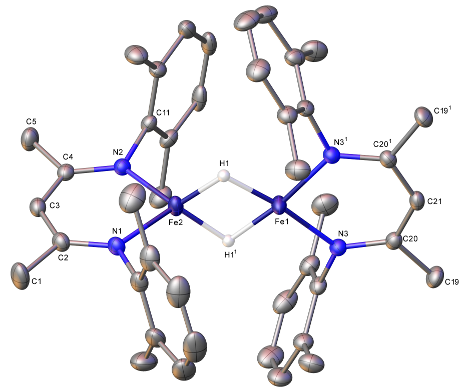


Table S4 Crystal data and structure refinement for **$[^{dmp}BDKFeH]_2$**.

| | |
|---|---|
| Identification code | **$[^{dmp}BDKFeH]_2$** |
| Empirical formula | $C_{42}H_{52}Fe_2N_4$ |
| Formula weight | 724.57 |
| Temperature/K | 150.00(10) |
| Crystal system | orthorhombic |
| Space group | Pnma |
| a/Å | 21.9805(5) |
| b/Å | 16.4818(4) |
| c/Å | 10.6196(2) |
| α/° | 90 |
| β/° | 90 |
| γ/° | 90 |
| Volume/Å$^3$ | 3847.25(15) |
| Z | 4 |
| $\rho_{calc}$g/cm$^3$ | 1.251 |
| μ/mm$^{-1}$ | 6.290 |
| F(000) | 1536.0 |
| Crystal size/mm$^3$ | 0.162 × 0.119 × 0.085 |
| Radiation | Cu Kα (λ = 1.54184) |
| 2Θ range for data collection/° | 9.248 to 145.172 |
| Index ranges | $-27 \leq h \leq 27$, $-18 \leq k \leq 20$, $-13 \leq l \leq 12$ |
| Reflections collected | 43158 |
| Independent reflections | 3952 [$R_{int}$ = 0.0642, $R_{sigma}$ = 0.0277] |
| Data/restraints/parameters | 3952/6/283 |
| Goodness-of-fit on F$^2$ | 1.027 |
| Final R indexes [I>=2σ (I)] | $R_1$ = 0.0390, $wR_2$ = 0.0969 |
| Final R indexes [all data] | $R_1$ = 0.0453, $wR_2$ = 0.1002 |
| Largest diff. peak/hole / e Å$^{-3}$ | 1.06/-0.32 |

In the structure of **$[^{dmp}BDKFeH]_2$**, the dipp group based on C11 was modelled to take account of 50:50 disorder about the crystallographic mirror plane on which atoms Fe1, Fe2, N1, N2, C1-C6, C9 and C21 are located. This mirror plane serves to generate the remaining half of the dimer from the motif which constitutes the asymmetric unit. H1 was located and refined without restraints.

## $Fe(^{dmp}BDK)_2$

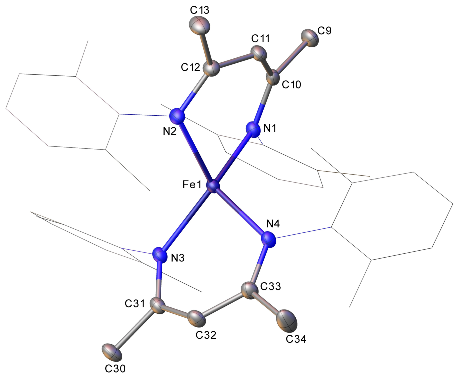


Table S5 Crystal data and structure refinement for **$Fe(^{dmp}BDK)_2$.**

| | |
|---|---|
| Identification code | **$Fe(^{dmp}BDK)_2$** |
| Empirical formula | $C_{42}H_{50}FeN_4$ |
| Formula weight | 666.71 |
| Temperature/K | 150.00(10) |
| Crystal system | monoclinic |
| Space group | $P2_1/n$ |
| a/Å | 10.9682(1) |
| b/Å | 17.6194(2) |
| c/Å | 18.8246(2) |
| α/° | 90 |
| β/° | 95.320(1) |
| γ/° | 90 |
| Volume/Å$^3$ | 3622.24(7) |
| Z | 4 |
| $\rho_{calc}$g/cm$^3$ | 1.223 |
| μ/mm$^{-1}$ | 3.589 |
| F(000) | 1424.0 |
| Crystal size/mm$^3$ | 0.286 × 0.21 × 0.123 |
| Radiation | Cu Kα (λ = 1.54184) |
| 2Θ range for data collection/° | 6.886 to 145.168 |
| Index ranges | $-9 \leq h \leq 13, -21 \leq k \leq 20, -23 \leq l \leq 23$ |
| Reflections collected | 38573 |
| Independent reflections | 7182 [$R_{int}$ = 0.0414, $R_{sigma}$ = 0.0290] |
| Data/restraints/parameters | 7182/0/436 |
| Goodness-of-fit on F$^2$ | 1.025 |
| Final R indexes [I>=2σ (I)] | $R_1$ = 0.0336, $wR_2$ = 0.0834 |
| Final R indexes [all data] | $R_1$ = 0.0383, $wR_2$ = 0.0864 |
| Largest diff. peak/hole / e Å$^{-3}$ | 0.23/-0.31 |

## 6 Method for Hydrogenation of Methylphenyl(silane-$d_2$)

Methylphenyl(silane-$d_2$(98%)) was prepared according to general procedure for deuteration of silanes. To a flame-dried 60 mL Teflon-sealed J-young ampoule containing pre-catalyst **1a** (5 mol%), a solution of methylphenyl (silane-$d_2$ (98%)) in $C_6D_6$ (500 $\mu$L) was added. The vessel was sealed, removed from the glovebox and subjected to freeze-pump-thaw cycles until a continuous vacuum was achieved. The ampoule was cooled in liquid nitrogen and backfilled with $H_2$ gas. The mixture was warmed to room temperature and stirred for 16 hours. The solution was transferred to a H-distillation tube and separated from **1a** by vacuum distillation. 1,3,5-Trimethoxybenzene (0.25 mmol) was added as a stock solution and spectroscopic yield and D-incorporation were determined by $^1$H NMR spectroscopy.

**Methylphenylsilane, 2a-H**

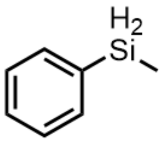


Hydrogen incorporation: 95%

Spectroscopic yield: 69%

**$^1$H NMR** ($C_6D_6$, 400 MHz) $\delta$ 7.47-7.44 (m, 2H, $^m$ArH), 7.19-7.12 (m, 3H, $^{o,p}$ArH), 4.48 (q, $J$=4.3 Hz, 1.9H, SiH), 0.18 (t, $J$=4.3 Hz, 3H, $CH_3$). NMR data consistent with a sample of the commercially available protonated starting material.

## 7 Method for Deuterium Labelling of Propylbenzene

Title compound was synthesised by modified literature procedure.[9] To a flame-dried 60 mL Teflon-sealed J-young ampoule containing pre-catalyst **1a** (5 mol%), $C_6D_6$ (500 $\mu$L) and diphenylsilane (46.3 $\mu$L, 0.25 mmol, 1.0 equiv.) were added. The vessel was sealed, removed from the glovebox and free-pump-thawed until a continuous vacuum was achieved. The ampoule was cooled in liquid nitrogen and backfilled with $D_2$ gas. The mixture was warmed to room temperature and stirred for 16 hours. The vessel was depressurised and returned to the glovebox. Allyl benzene (33.1 $\mu$L, 0.25 mmol, 1.0 equiv.) and aniline (22.8 $\mu$L, 0.25 mmol, 1.0 equiv.) were added. The tube was sealed and the reaction was allowed to proceed for 16 hours at room temperature. The solution was transferred to a H-distillation tube and separated from **1a** by vacuum distillation. 1,3,5-Trimethoxybenzene (0.25 mmol) was added as a stock solution and spectroscopic yield and D-incorporation were determined by $^1$H and $^2$H NMR spectroscopy.

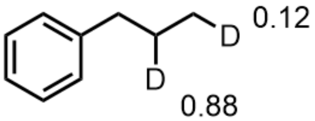


Spectroscopic yield: 95%

**$^1$H NMR** ($C_6D_6$, 400 MHz) $\delta$ 7.18-7.15 (m, 2H, $^m$ArH), 7.09-7.04 (m, 3H, $^{o,p}$ArH), 2.44-2.40 (m, 2H, $ArCH_2$), 1.53-1.43 (m, 1.1H, CHD), 0.84-0.80 (m, 2.9H, $CH_3$). **$^2$H NMR** ($C_6H_6$, 77 MHz) $\delta$ 1.50 (s, 0.92D), 0.82 (s, 0.08D). **$^{13}$C{$^1$H} NMR** ($CDCl_3$, 125 MHz) $\delta$ 142.4, 128.4, 128.2, 125.6, 37.9, 24.1 (t, $J$=19.5 Hz, CD), 13.4. NMR data consistent with a sample of the commercially available protonated starting material.

# 8 Reaction Monitoring Experiments

## 8.1 General Method

To a high-pressure J-young NMR tube containing [Fe] (5 mol%) and 1,3,5-trimethoxybenzene (0.25 mmol), $C_6D_6$ (500 $\mu$L) and silane (0.25 mmol) were added. The tube was sealed, removed from the glovebox and subjected to freeze-pump-thaw cycles until a continuous vacuum was achieved. The tube was cooled in liquid nitrogen and backfilled with $D_2$ gas. The mixture was warmed to room temperature and the reaction was monitored for 16 hours. D-incorporation was determined by $^1$H NMR spectroscopy.

## 8.2 Reaction Monitoring Plots

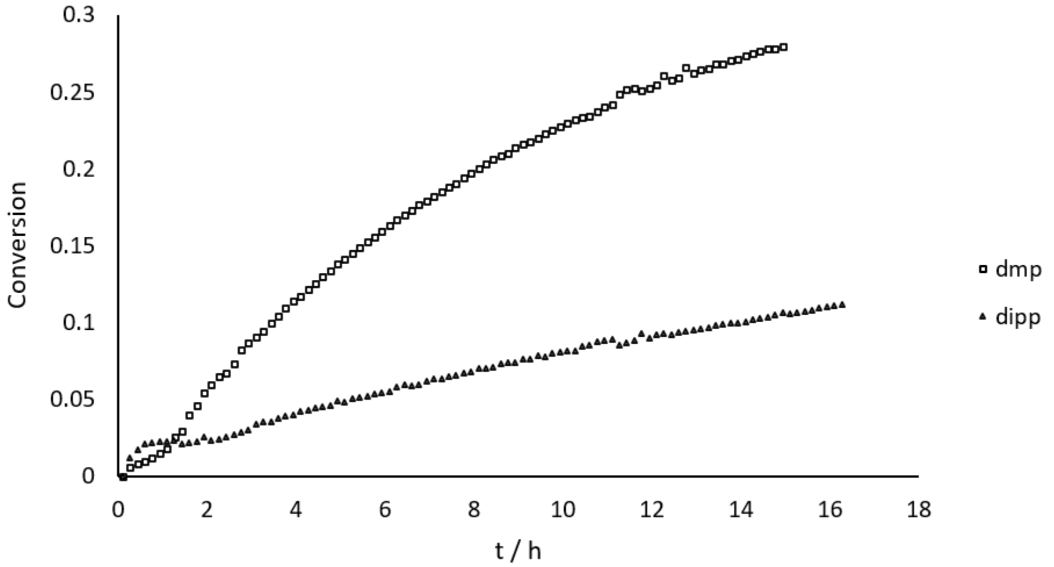


**Figure S1:** Plot of conversion to $PhSiD_3$ versus time, catalysed by **1a** and **1b**, reported as a fraction of conversion to $PhSiD_3$.

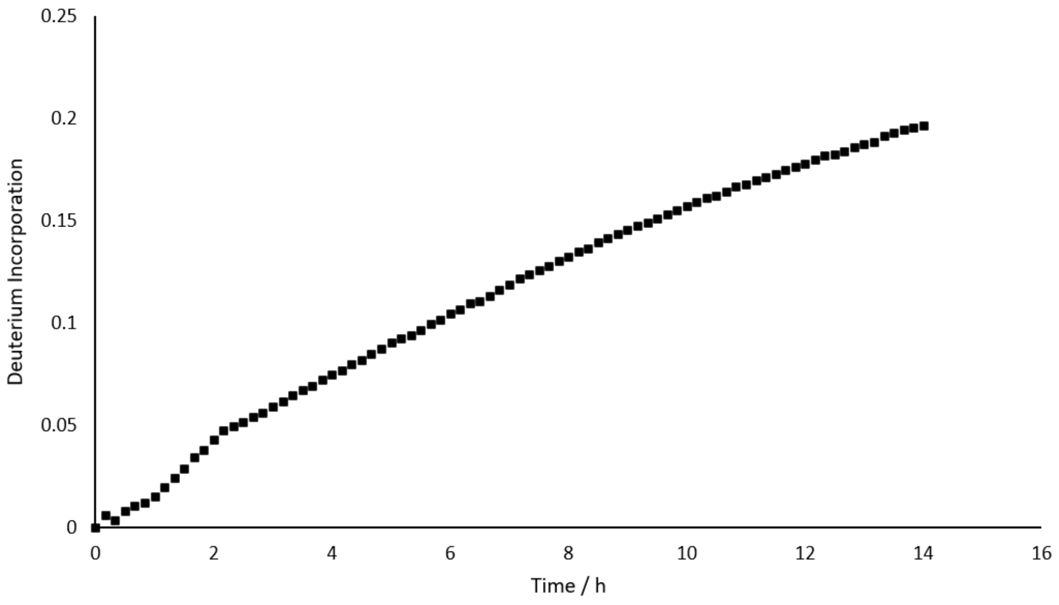


**Figure S2:** Plot of conversion to $PhSiD_3$ versus time at 298 K, catalysed by **4a**, reported as a fraction of conversion to $PhSiD_3$.

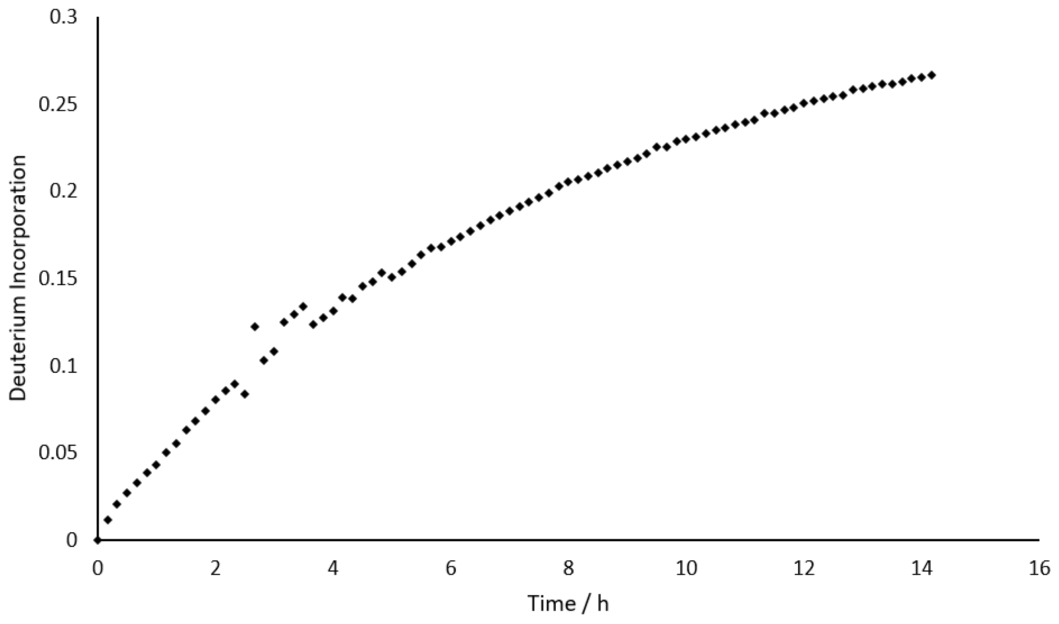


**Figure S3:** Plot of conversion to $PhSiD_3$ versus time at 308 K, catalysed by **4a**, reported as a fraction of conversion to $PhSiD_3$.

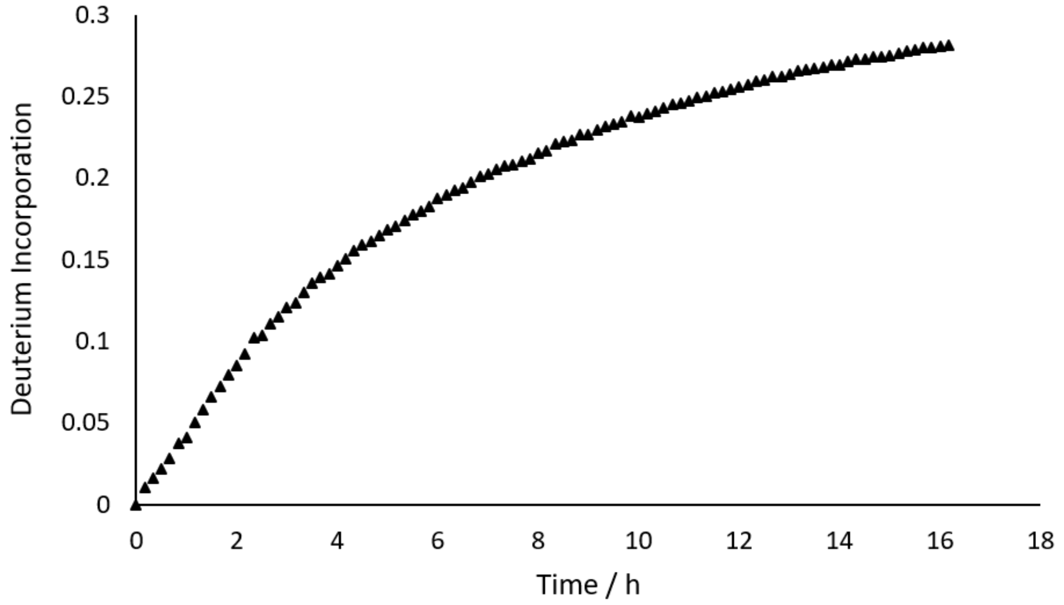


**Figure S4:** Plot of conversion to $PhSiD_3$ versus time at 318 K, catalysed by **4a**, reported as a fraction of conversion to $PhSiD_3$.

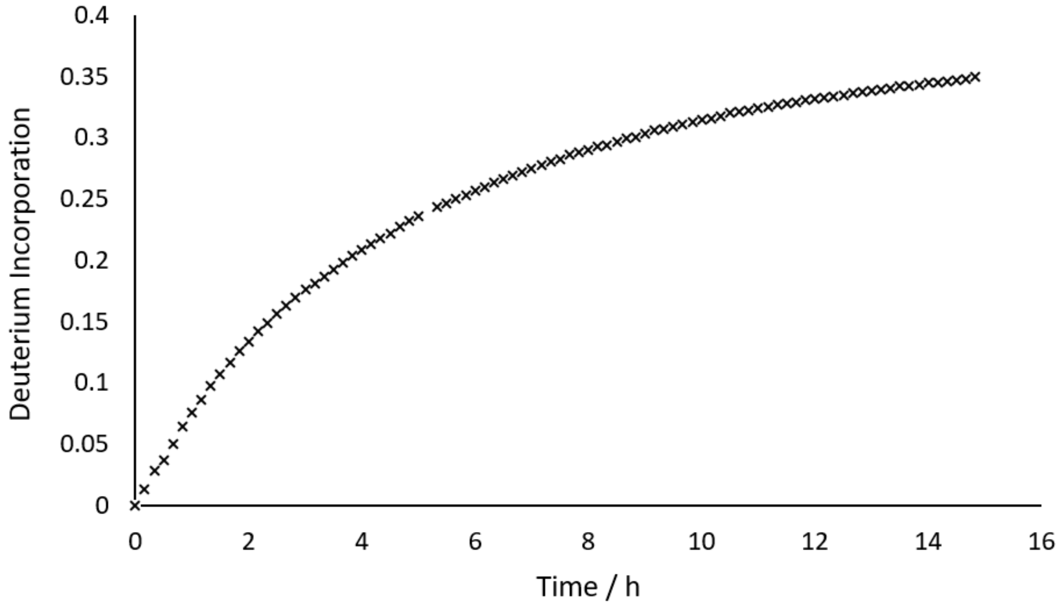


**Figure S5:** Plot of conversion to $PhSiD_3$ versus time at 328 K, catalysed by **4a**, reported as a fraction of conversion to $PhSiD_3$.

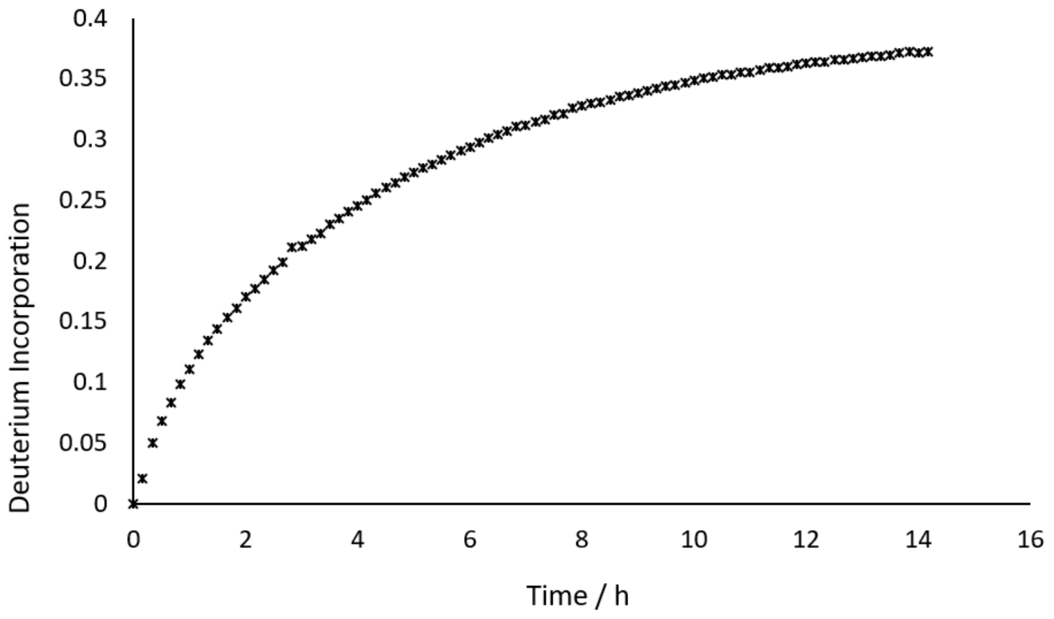


**Figure S6:** Plot of conversion to $PhSiD_3$ versus time at 338 K, catalysed by **4a**, reported as a fraction of conversion to $PhSiD_3$.

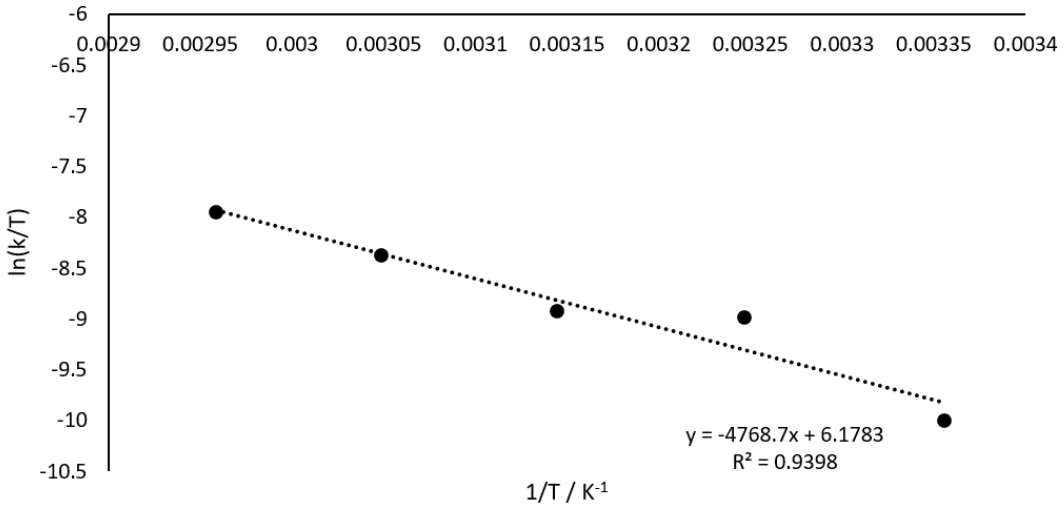


**Figure S7:** Eyring plot for deuteration of $PhSiH_3$, catalysed by **4a**.

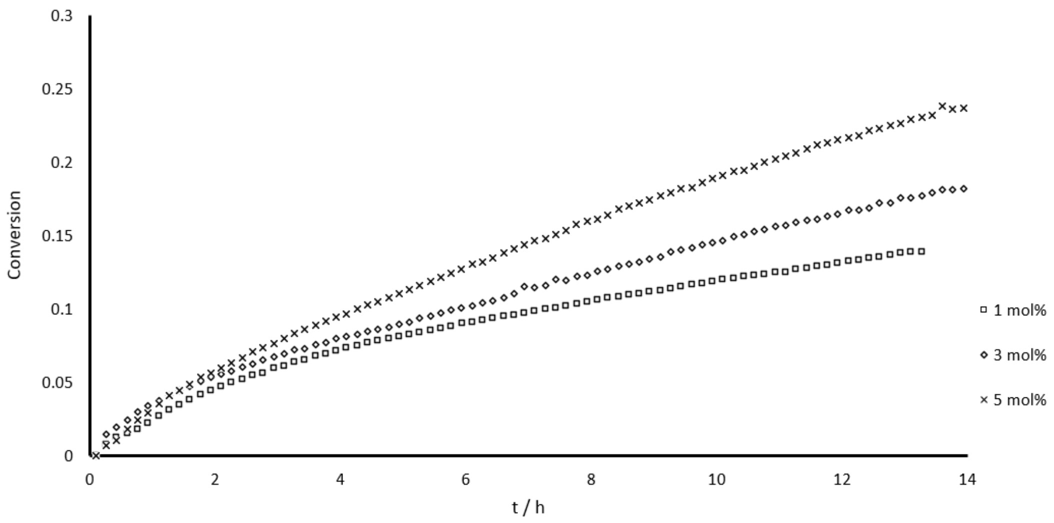


**Figure S8:** Plot of conversion to $PhSiD_3$ versus time, catalysed by **4a** (calculated as monomeric **4a**), reported as a fraction of conversion to $PhSiD_3$.

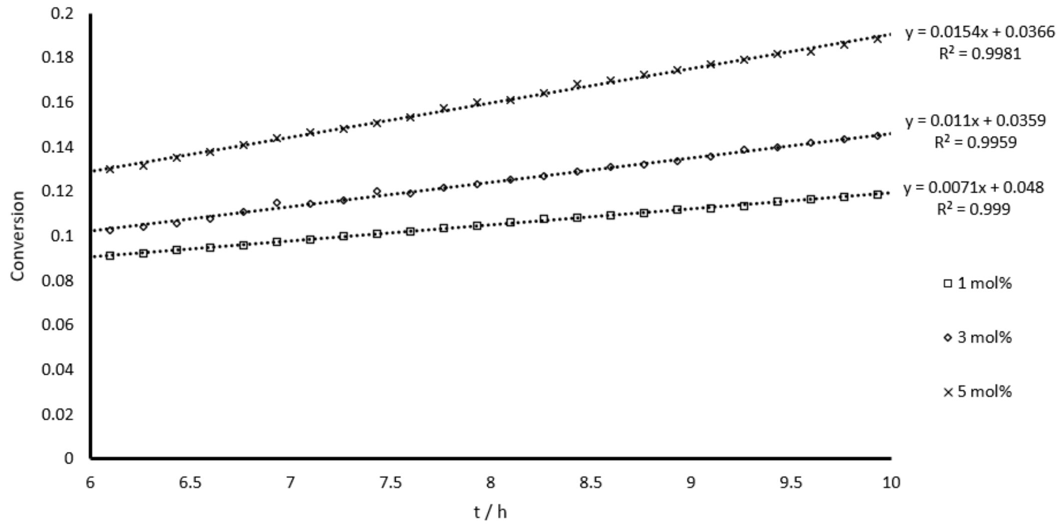


**Figure S9:** Plot of conversion to $PhSiD_3$ versus time, catalysed by **4a** (calculated as monomeric **4a**), reported as a fraction of conversion to $PhSiD_3$.

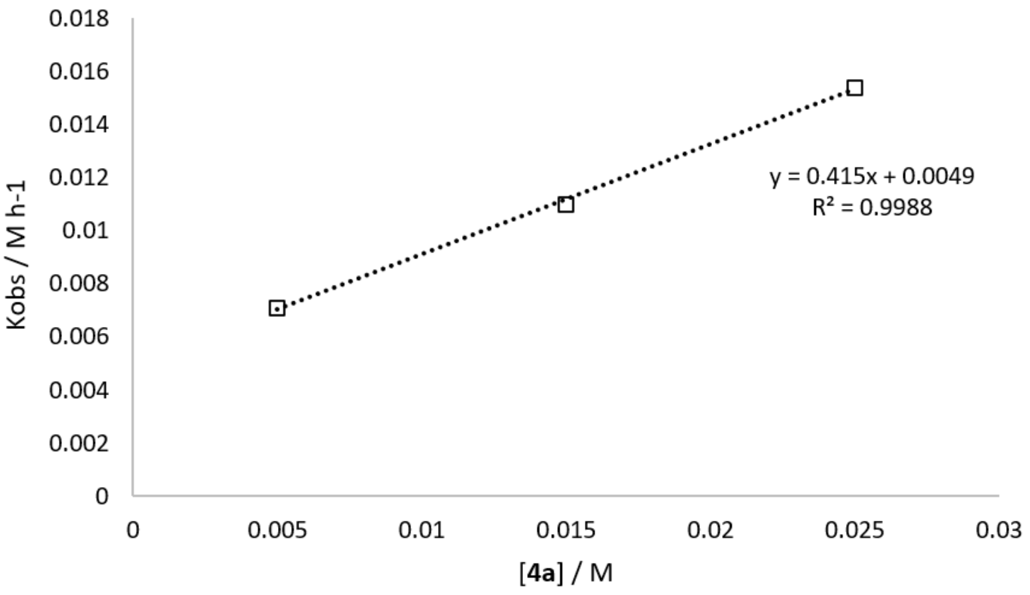


**Figure S10:** Plot of rate of H/D exchange versus catalyst concentration, catalysed by **4a** (calculated as monomeric **4a**).

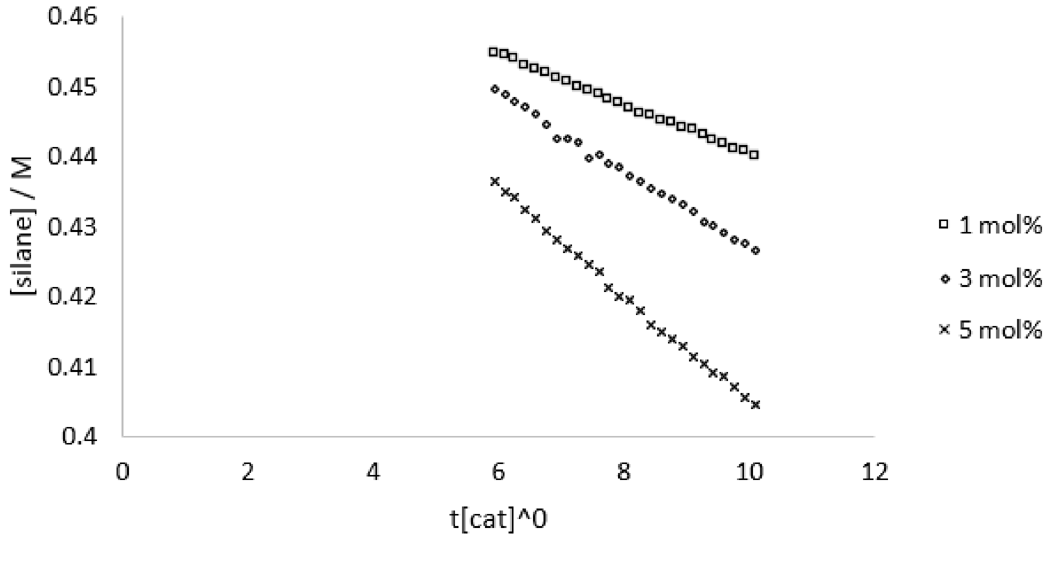


**Figure S11:** Plot of $PhSiH_3$ concentration versus $t[cat]^0$, catalysed by **4a** (calculated as monomeric **4a**).

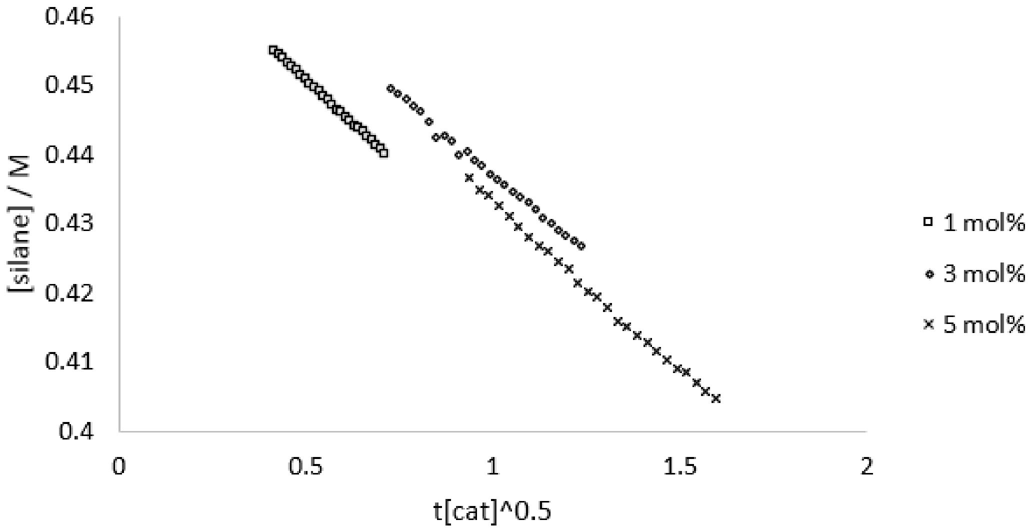


**Figure S12:** Plot of $PhSiH_3$ concentration versus t[cat]$^{0.5}$, catalysed by **4a** (calculated as monomeric **4a**).

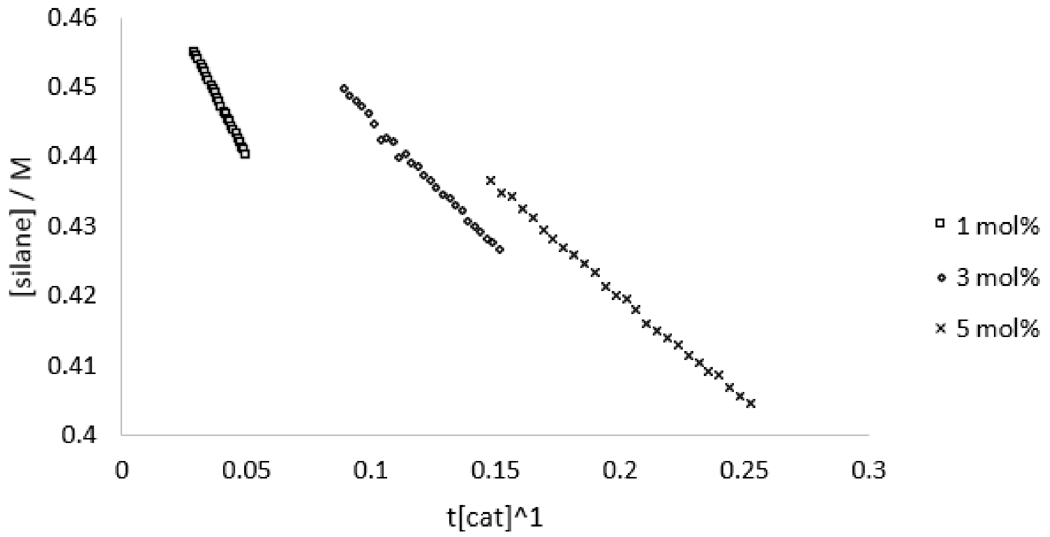


**Figure S13:** Plot of $PhSiH_3$ concentration versus t[cat]$^{1}$, catalysed by **4a** (calculated as monomeric **4a**).

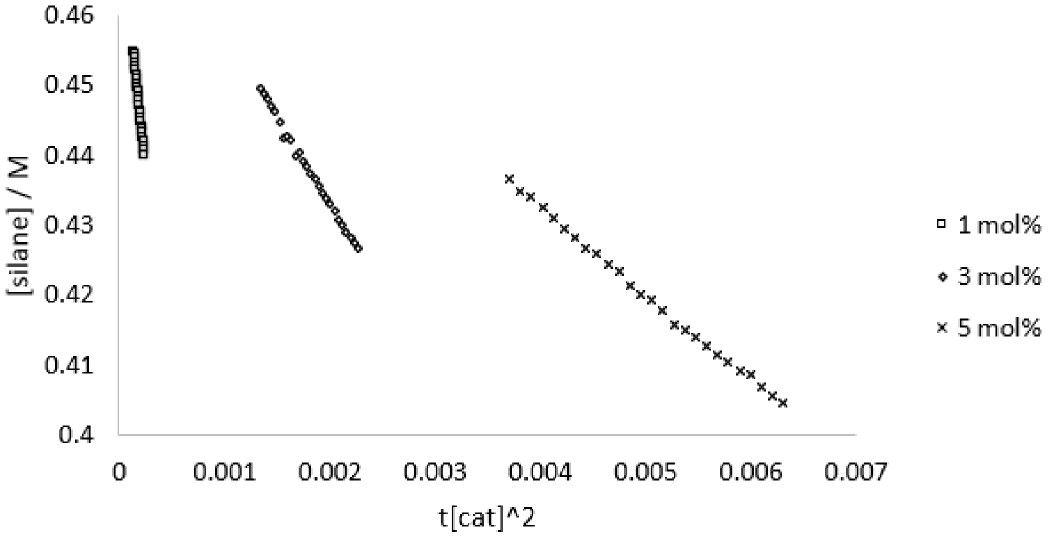


**Figure S14:** Plot of $PhSiH_3$ concentration versus t$[cat]^2$, catalysed by **4a** (calculated as monomeric **4a**).

# 9 Substrate Synthesis

**(4-(Dimethylsilyl)phenyl)methanol**

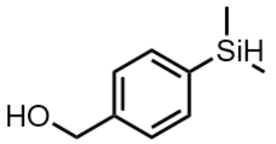


Title compound was synthesised according to literature procedure.[10] To a solution of (4-bromophenyl)methanol (2.81 g, 15.0 mmol, 1.0 equiv.) in THF (50 mL) at -78 °C, $^{n}$butyllithium (2.5M solution in hexanes, 15.0 mL, 37.5 mmol, 2.5 equiv.) was added dropwise over 30 minutes. The mixture was stirred at this temperature for 2 hours before the dropwise addition of chlorodimethylsilane (2.25 mL, 20.0 mmol, 1.3 equiv.). The solution was warmed to room temperature and stirred for 16 hours. The reaction was quenched with saturated aqueous $NH_4Cl$ solution (50 mL) and extracted with DCM (3 × 50 mL). The organic extracts were combined, washed with water (50 mL) and brine (50 mL), dried over $MgSO_4$, filtered and concentrated under reduced pressure. The crude product was purified by column chromatography ($SiO_2$, pet. ether/EtOAc (4:1)) yielding (4-(dimethylsilyl)phenyl)methanol as a colourless oil (1.19 g, 7.14 mmol, 48%). **$^1$H NMR** ($CDCl_3$, 400 MHz) $\delta$ 7.55 (d, $J$=7.9 Hz, 2H), 7.37 (d, $J$=7.6 Hz, 2H), 4.69 (s, 2H), 4.44 (hept, $J$=3.7 Hz, SiH), 0.35 (d, $J$=3.7 Hz, 6H); **$^{13}$C{$^1$H} NMR** ($CDCl_3$, 125 MHz) $\delta$ 142.0, 137.0, 134.4, 126.6, 65.4, -3.6; **$^{29}$Si NMR** ($CDCl_3$, 125 MHz) $\delta$ -17.3. NMR data in agreement with the literature.[10]

**(4-(Chloromethyl)phenyl)dimethylsilane**

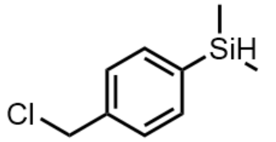

Title compound was synthesised according to literature procedure.[10] To a stirred solution of (4-(dimethylsilyl) phenyl)methanol (1.00 g, 6.01 mmol, 1.0 equiv.) in DCM (10 mL), triethylamine (1.01 mL, 7.22 mmol, 1.2 equiv.) was added dropwise. 4-Methylbenzenesulfonyl chloride (1.26 g, 6.61 mmol, 1.1 equiv.) was added and the solution was stirred for 16 hours. The mixture was diluted in DCM (10 mL) and washed with water (10 mL) and brine (10 mL). Organic extract was dried over $MgSO_4$, filtered and concentrated under reduced pressure. Crude product was purified by FCC ($SiO_2$, pentane) yielding (4-(chloromethyl)phenyl)dimethylsilane as a colourless oil (394 mg, 2.13 mmol, 36%). **$^1$H NMR** ($CDCl_3$, 400 MHz) $\delta$ 7.55 (d, $J$=7.9 Hz, 2H), 7.39 (d, $J$=7.9 Hz, 2H), 4.59 (s, 2H), 4.43 (hept, $J$=3.6 Hz, SiH), 0.35 (d, $J$=3.8 Hz, 6H); **$^{13}$C{$^1$H} NMR** ($CDCl_3$, 125 MHz) $\delta$ 138.5, 138.1, 134.6, 46.3, -3.7; **$^{29}$Si NMR** ($CDCl_3$, 125 MHz) $\delta$ -17.0. NMR data in agreement with the literature.[10]

**4-Bromostyrene**

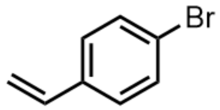

Title compound was synthesised according to literature procedure.[11] To a stirred suspension of methyltriphenylphosphonium iodide (5.33 g, 13.2 mmol, 1.1 equivs.) in THF (50 mL) at 0 °C, $^n$butyllithium (2.5M solution in hexanes, 6.6 mL, 16.5 mmol, 1.4 equiv.) was added dropwise over 30 minutes and left stirring for a further 2 hours. A solution of 4-bromobenzaldehyde (2.22 g, 12.0 mmol, 1.0 equiv.) in THF (10 mL) was added dropwise, warmed to room temperature and stirred for 16 hours. $Et_2O$ (100 mL) was added and the solution was washed with water (100 mL) and brine (100 mL), dried over $MgSO_4$, filtered and concentrated under reduced pressure. Crude product was purified by FCC ($SiO_2$, pentane) yielding 4-bromostyrene as a colourless oil (739 mg, 4.04 mmol, 34%). **$^1$H NMR** ($CDCl_3$, 400 MHz) $\delta$ 7.37 (d, $J$=8.4 Hz, 2H), 7.20 (d, $J$=8.5 Hz, 2H), 6.58 (dd, $J$=17.6, 10.9 Hz, 1H), 5.66 (d, $J$=18.1 Hz, 1H), 5.20 (d, $J$=10.9 Hz, 1H); **$^{13}$C{$^1$H} NMR** ($CDCl_3$, 100 MHz) $\delta$ 136.6, 135.9, 131.8, 127.9, 121.7, 114.8. NMR data in agreement with the literature.[11]

**Dimethyl(4-vinylphenyl)silane**

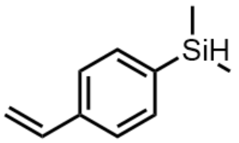

Title compound was synthesised by modified literature procedure.[10] To a solution of 4-bromostyrene (719 mg, 3.93 mmol, 1 equiv.) in THF (10 mL) at -78 °C, $^n$butyllithium (2.5M solution in hexanes, 1.89 mL, 1.2 equiv.) was added dropwise over 30 minutes. The mixture was stirred at this temperature for 2 hours before the dropwise addition of chlorodimethylsilane (873 $\mu$L, 7.86 mmol, 2.0 equiv.). The solution was warmed to room temperature

and stirred for 16 hours. The reaction was quenched with saturated aqueous $NH_4Cl$ solution (10 mL) and extracted with diethylether (3 × 10 mL). The organic extracts were combined, washed with water (20 mL) and brine (20 mL), dried over $MgSO_4$, filtered and concentrated under reduced pressure. The crude product was purified by FCC ($SiO_2$, pentane) yielding dimethyl(4-vinylphenyl)silane as a colourless oil (495 mg, 3.05 mmol, 78%). **$^1$H NMR** ($CDCl_3$, 400 MHz) $\delta$ 7.53 (d, $J$=8.0 Hz, 2H), 7.42 (d, $J$=7.9 Hz, 2H), 6.73 (dd, $J$=17.6, 10.9 Hz, 1H), 5.80 (d, $J$=17.6 Hz, 1H), 5.28 (d, $J$=10.9 Hz, 1H), 4.44 (hept, $J$=3.8 Hz, 1H), 0.36 (d, $J$=3.8 Hz, 6H); **$^{13}$C{$^1$H} NMR** ($CDCl_3$, 125 MHz) $\delta$ 138.5, 137.2, 137.0, 134.4, 125.8, 114.4, -3.62; **$^{29}$Si NMR** ($CDCl_3$, 125 MHz) $\delta$ -17.3. NMR data in agreement with the literature.[10]

# 10 Substrate Scope Spectra

**Methylphenyl(silane-d$_2$), 2a**

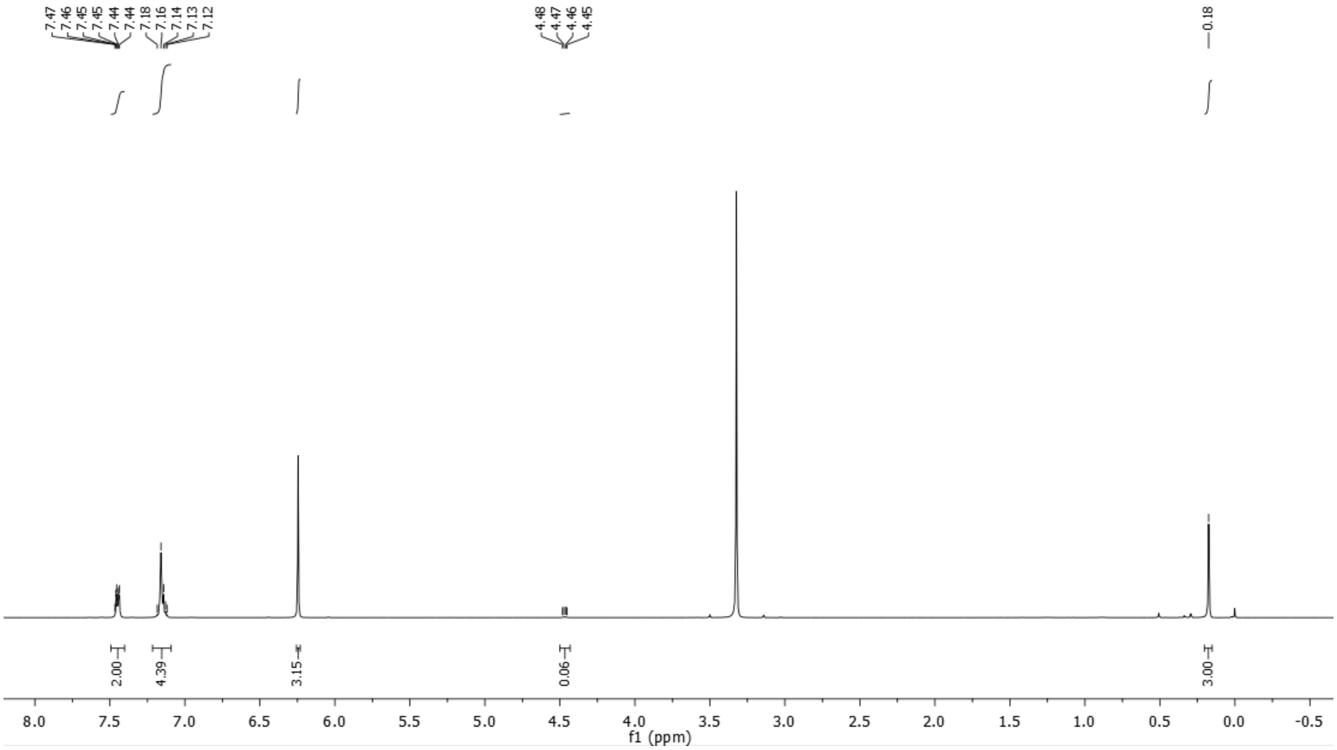


**Figure S15:** $^1$H NMR spectrum of methylphenyl(silane-d$_2$) and 1,3,5-trimethoxybenzene.

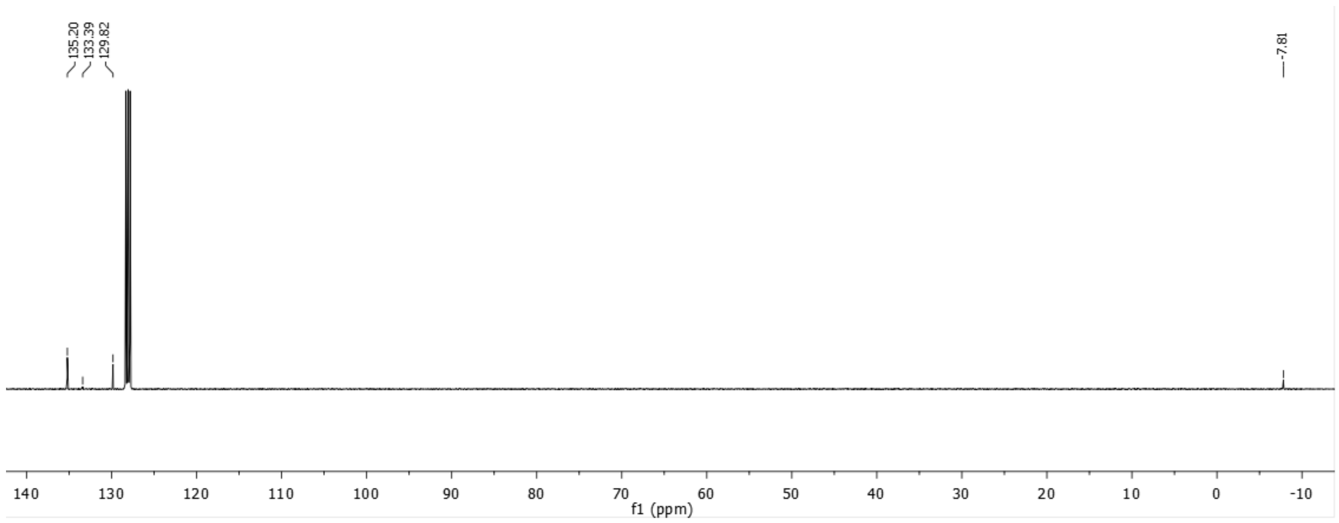


**Figure S16:** $^{13}C\{^{1}H\}$ NMR spectrum of methylphenyl(silane-$d_2$).

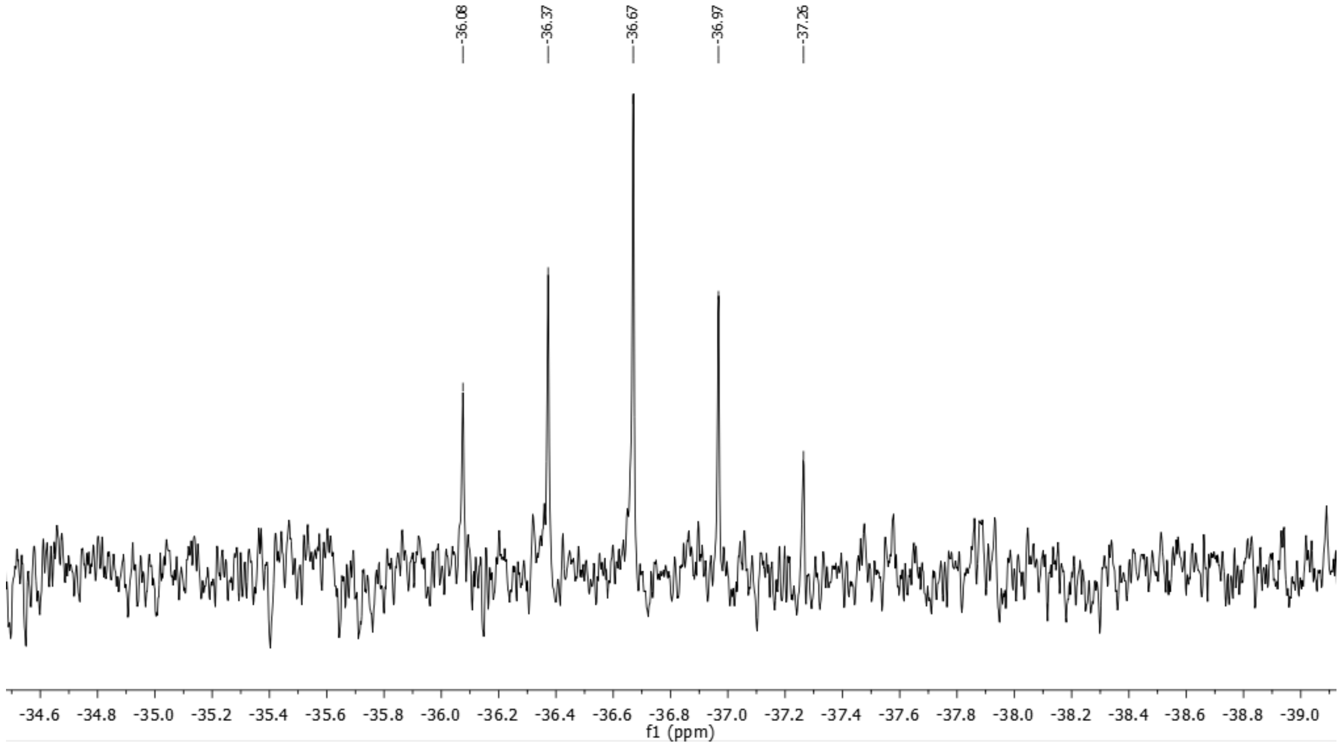


**Figure S17:** $^{29}Si$ NMR spectrum of methylphenyl(silane-$d_2$) and 1,3,5-trimethoxybenzene.

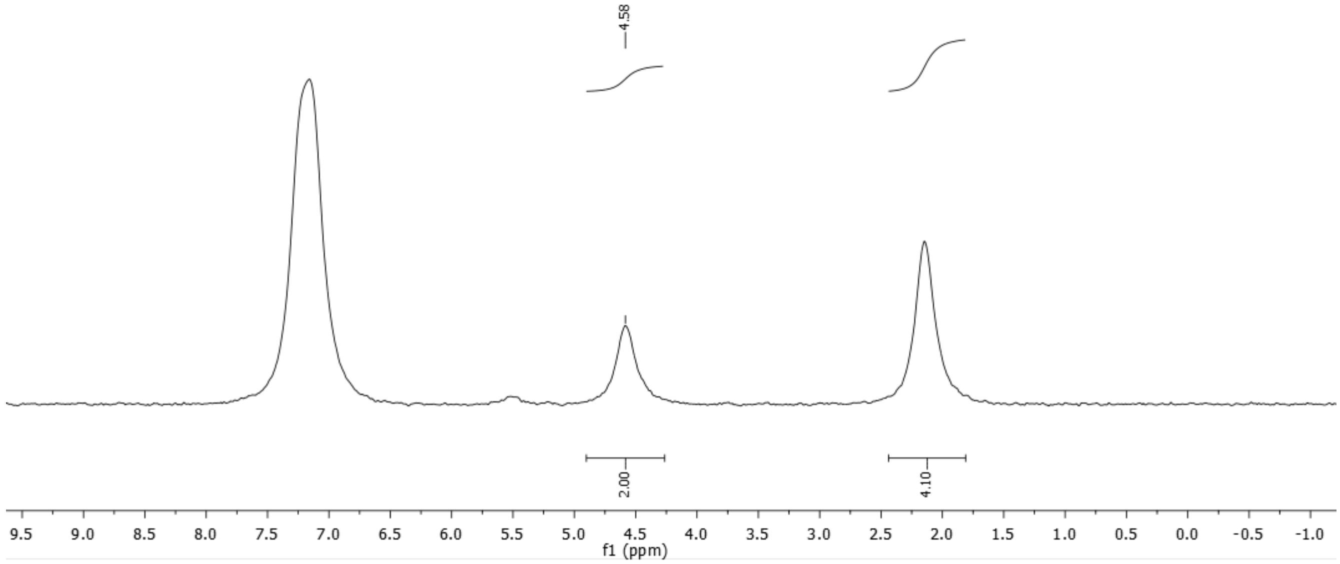


**Figure S18:** $^{2}$H NMR spectrum of methylphenyl(silane-$d_2$) and toluene-$d_8$.

**Phenyl(silane-$d_3$), 2b (catalysed by 1a)**

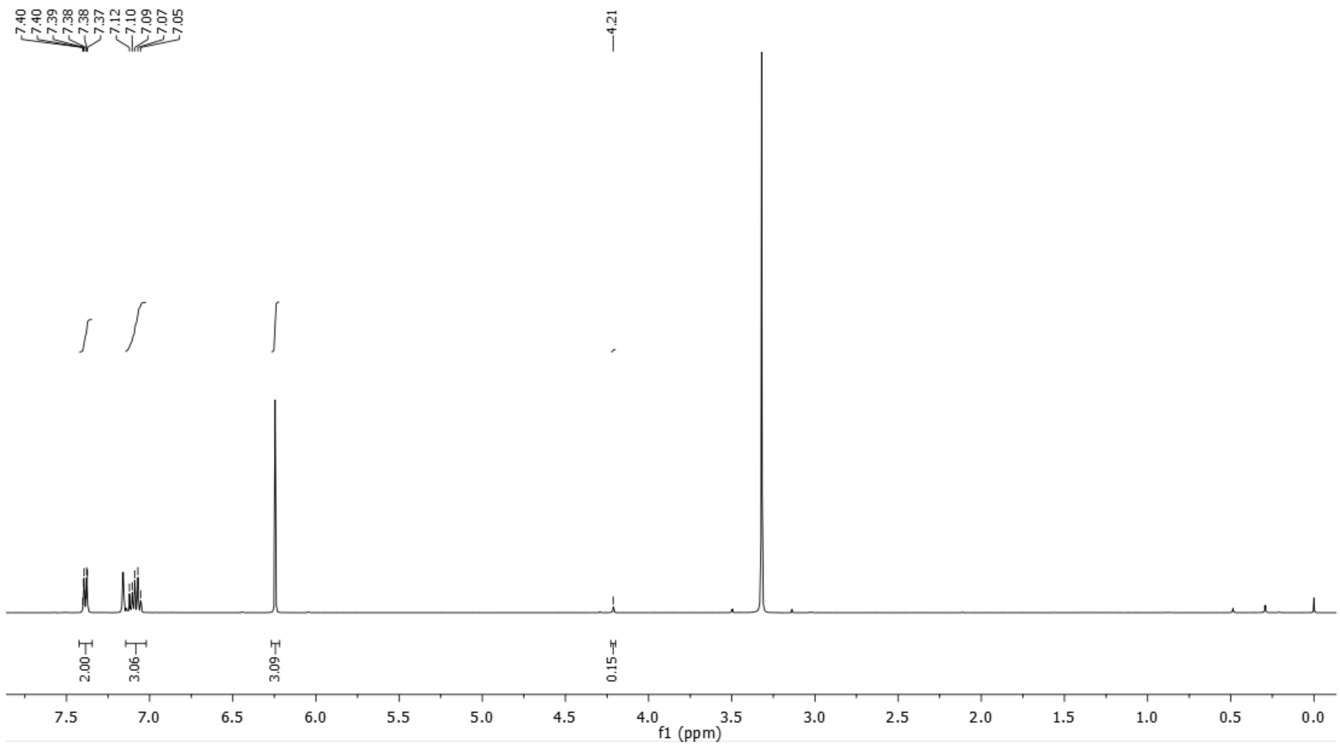


**Figure S19:** $^{1}$H NMR spectrum of phenyl(silane-$d_3$) and 1,3,5-trimethoxybenzene.

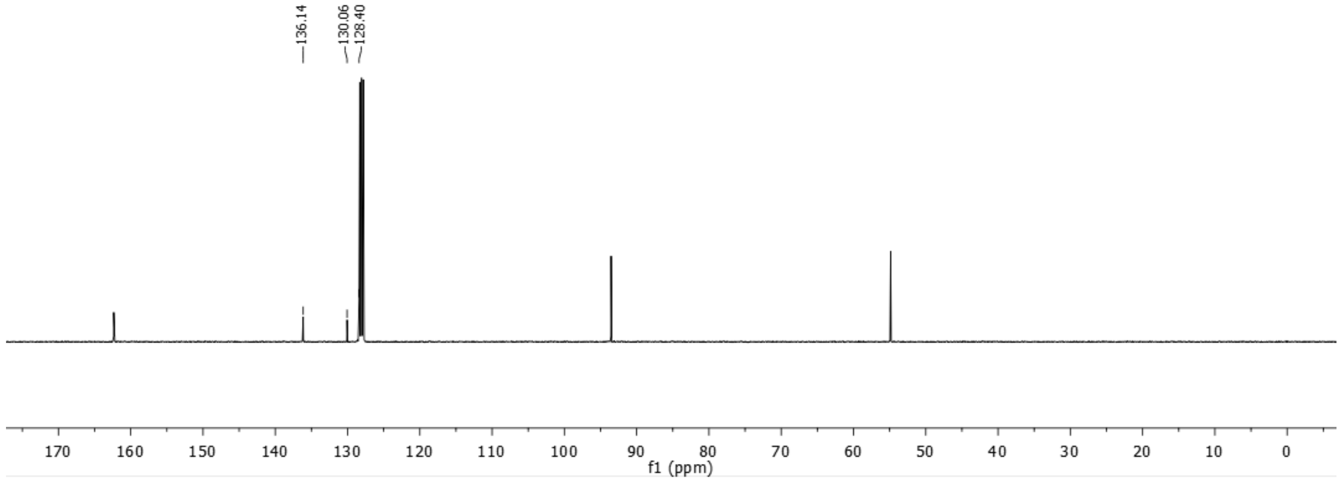


**Figure S20:** $^{13}C\{^1H\}$ NMR spectrum of phenyl(silane-d$_3$) and 1,3,5-trimethoxybenzene.

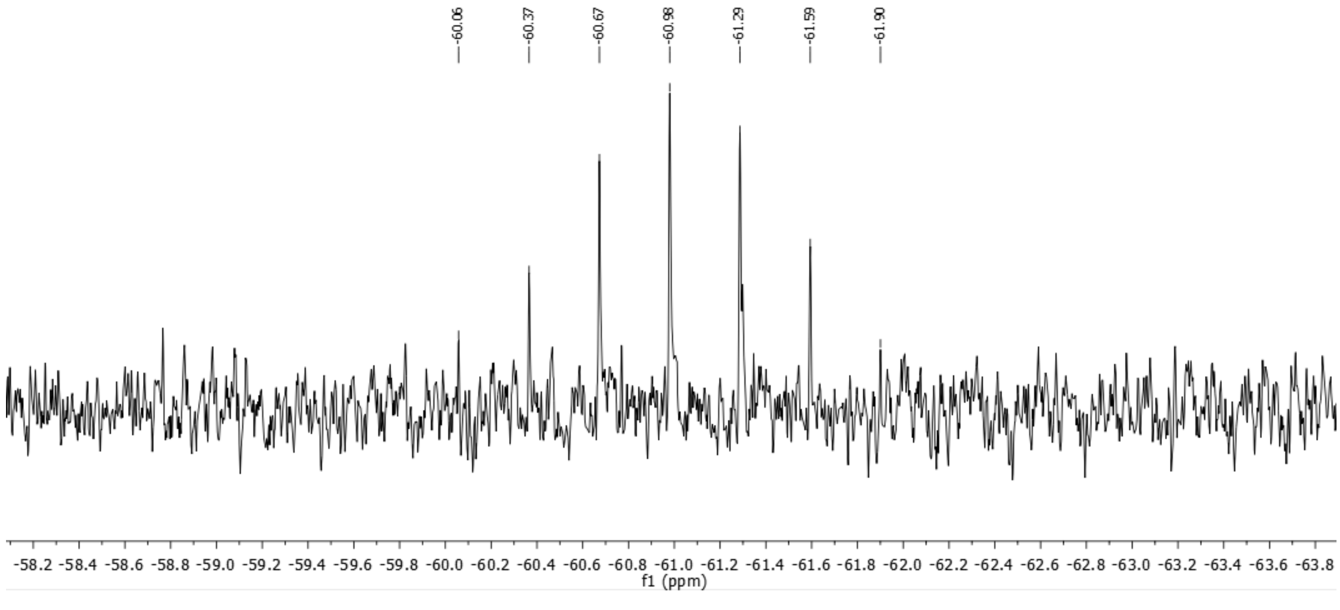


**Figure S21:** $^{29}Si$ NMR spectrum of phenyl(silane-d$_3$) and 1,3,5-trimethoxybenzene.

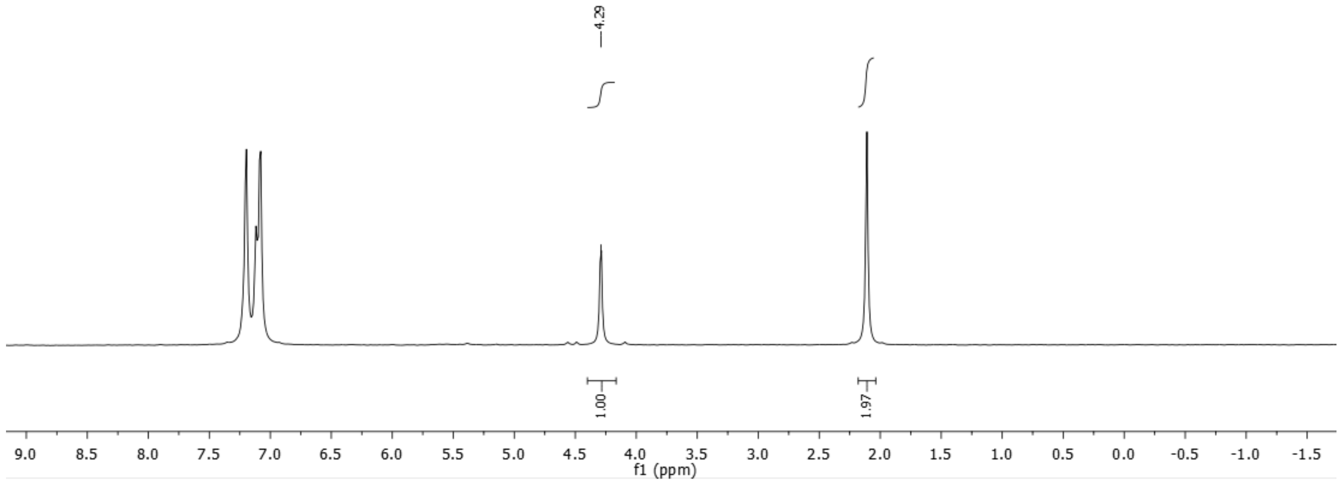


**Figure S22:** $^2$H NMR spectrum of phenyl(silane-$d_3$) and toluene-$d_8$.

**Phenyl(silane-$d_3$), 2b (catalysed by 1b)**

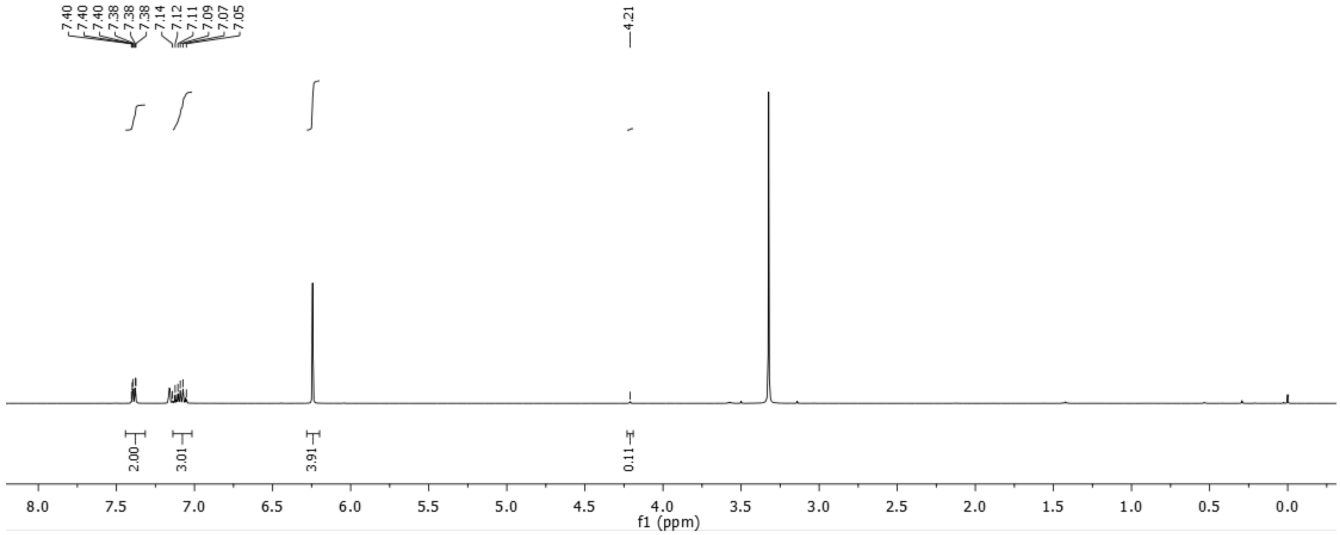


**Figure S23:** $^1$H NMR spectrum of phenyl(silane-$d_3$) and 1,3,5-trimethoxybenzene.

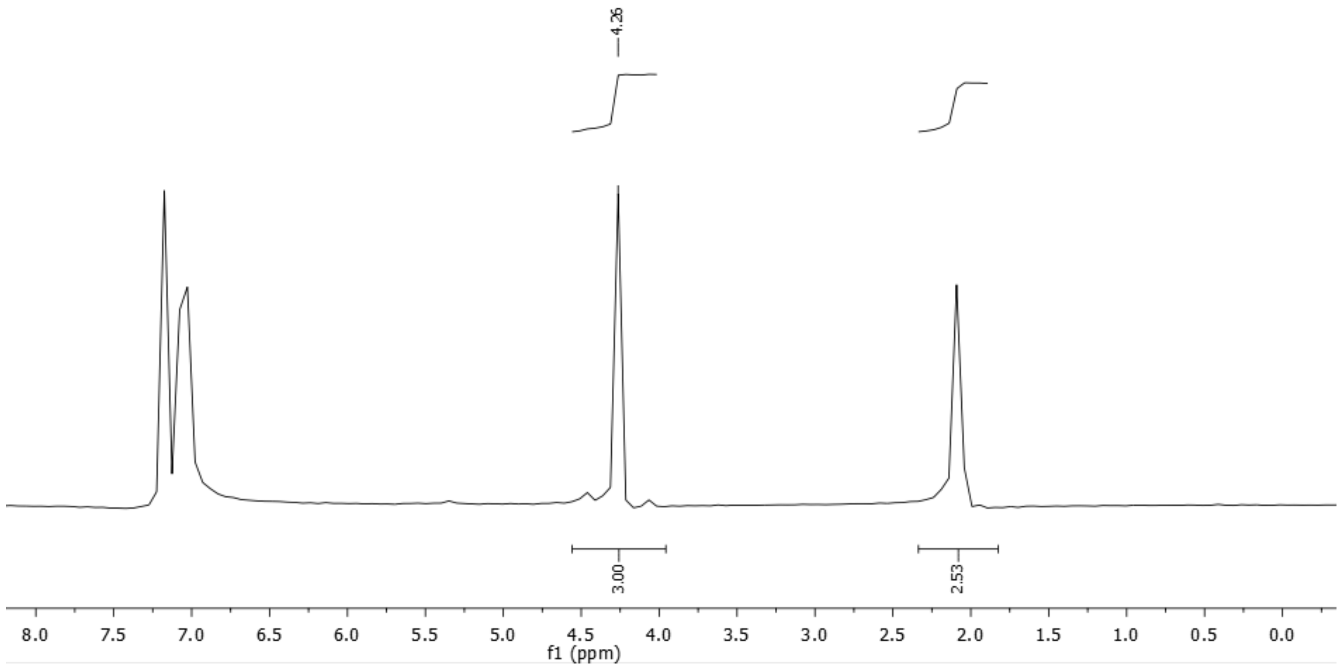


**Figure S24:** $^{2}$H NMR spectrum of phenyl(silane-$d_3$) and toluene-$d_8$.

**Diphenyl(silane-$d_2$), 2c**

**0.25 mmol**

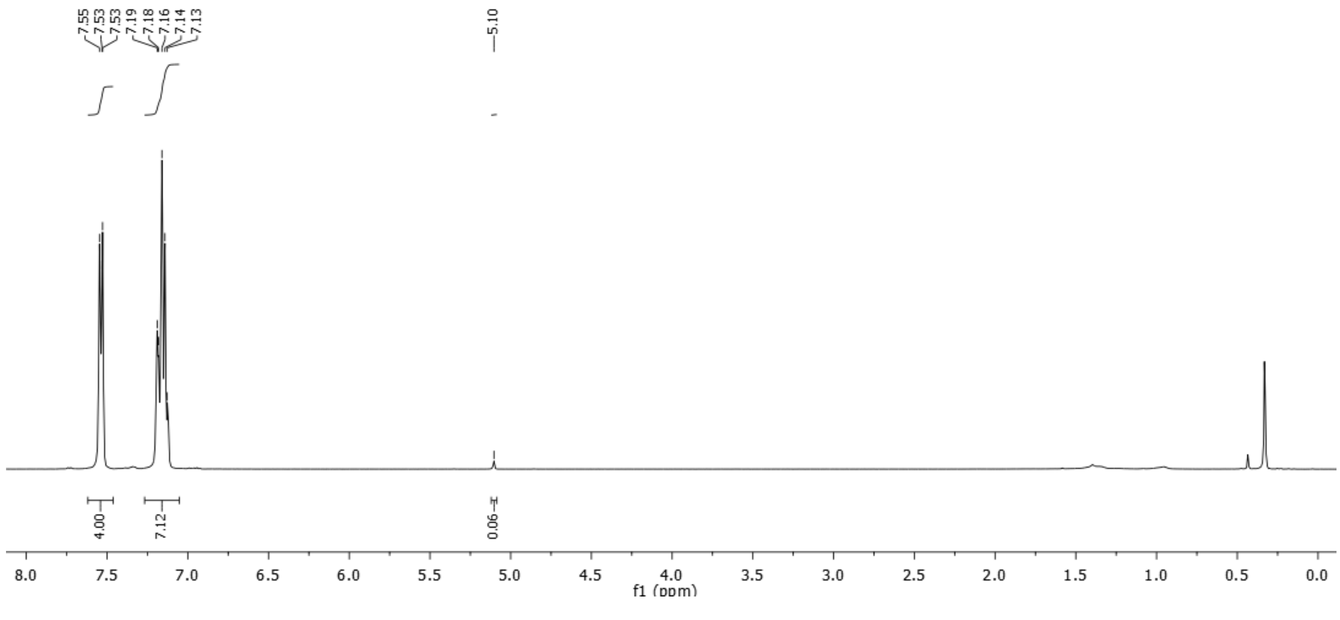


**Figure S25:** $^{1}$H NMR spectrum of diphenyl(silane-$d_2$).

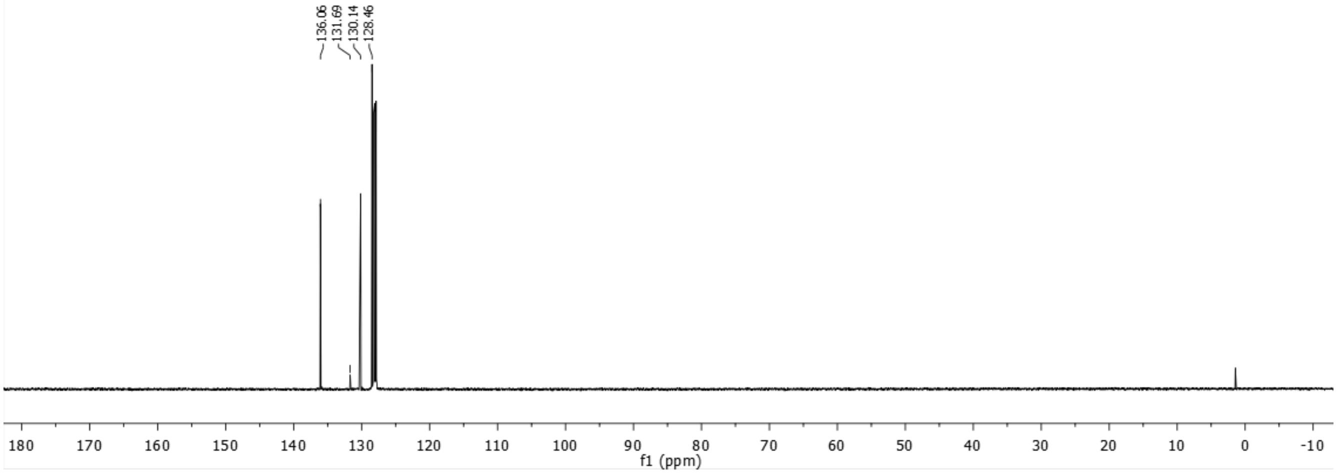


**Figure S26:** $^{13}C\{^{1}H\}$ NMR spectrum of diphenyl(silane-d$_2$).

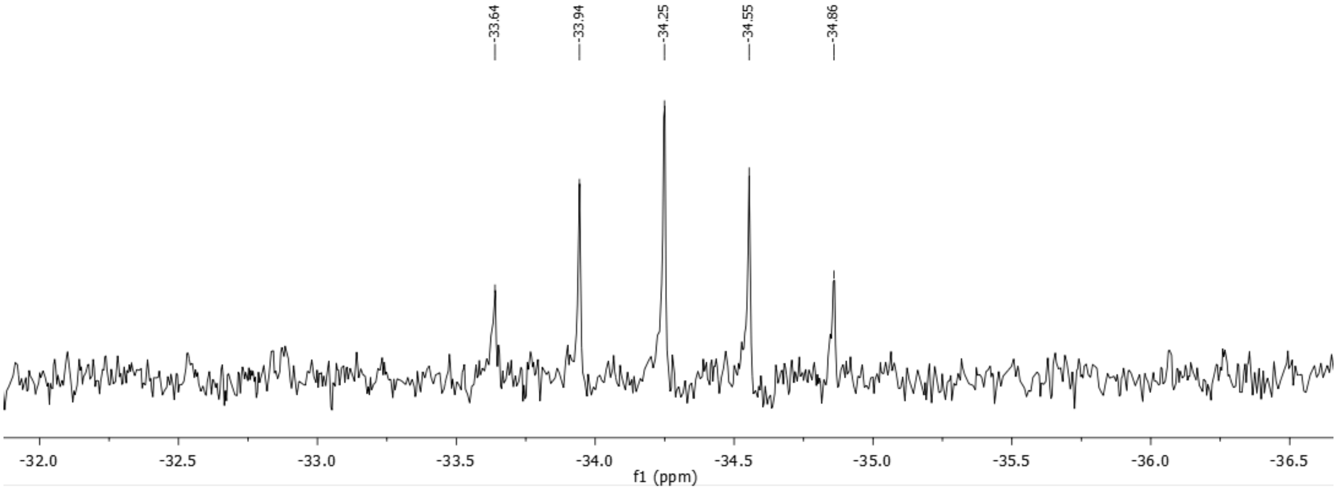


**Figure S27:** $^{29}Si$ NMR spectrum of diphenyl(silane-d$_2$).

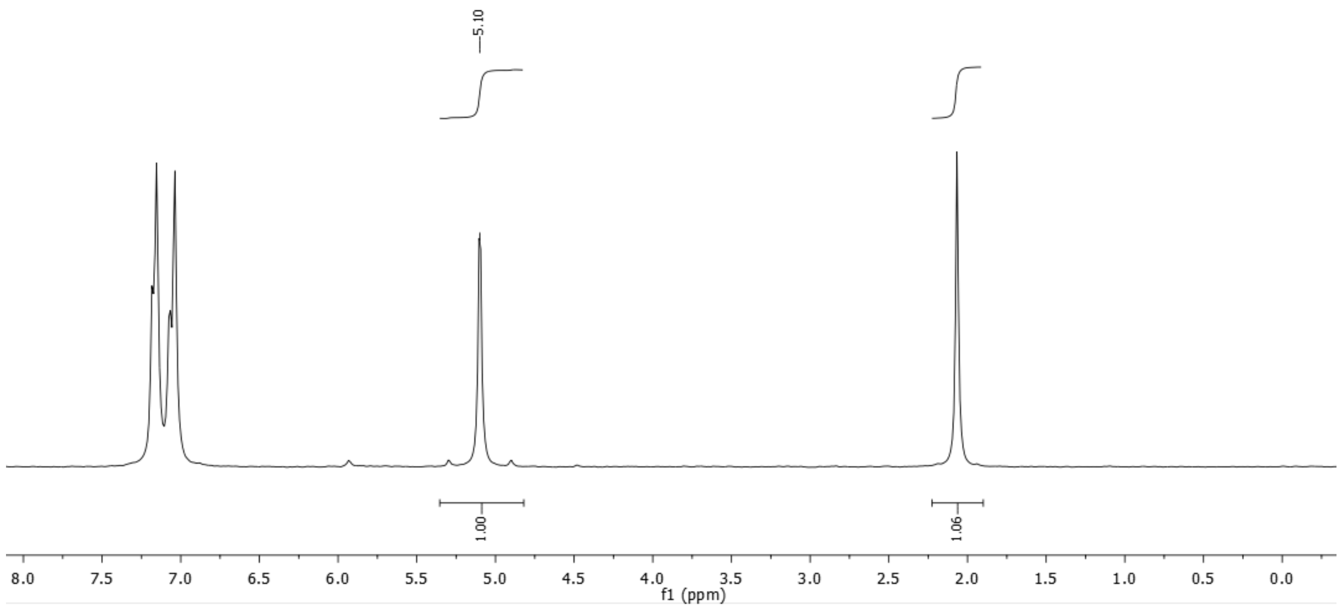


**Figure S28:** $^{2}H$ NMR spectrum of diphenyl(silane-$d_2$) and toluene-$d_8$.

<u>**2.5 mmol**</u>

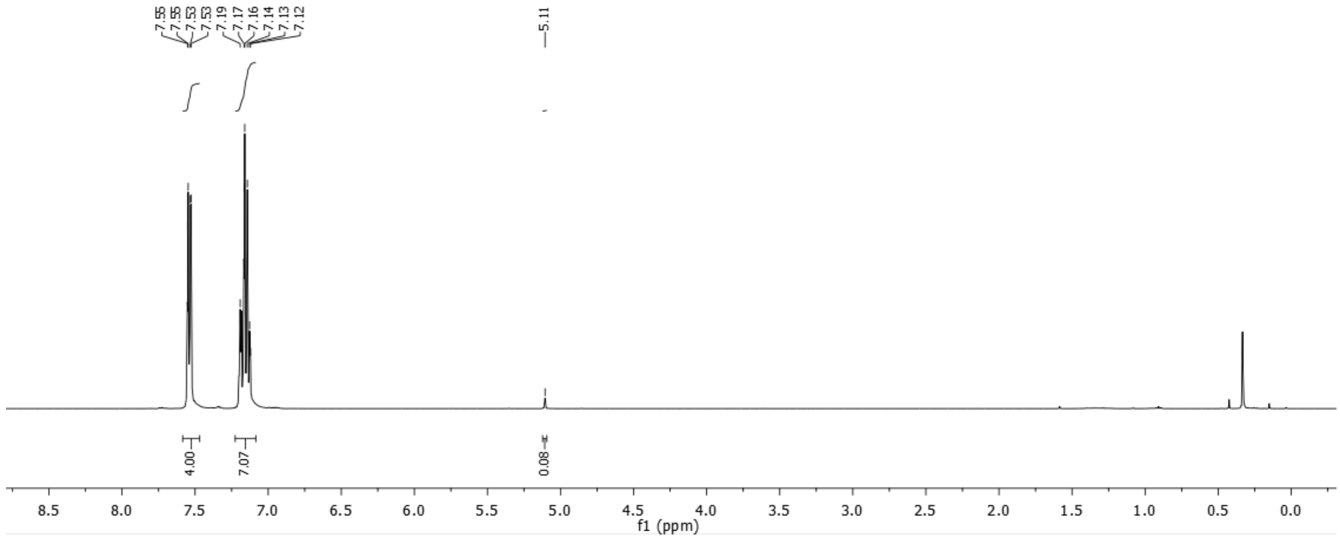


**Figure S29:** $^{1}H$ NMR spectrum of diphenyl(silane-$d_2$).

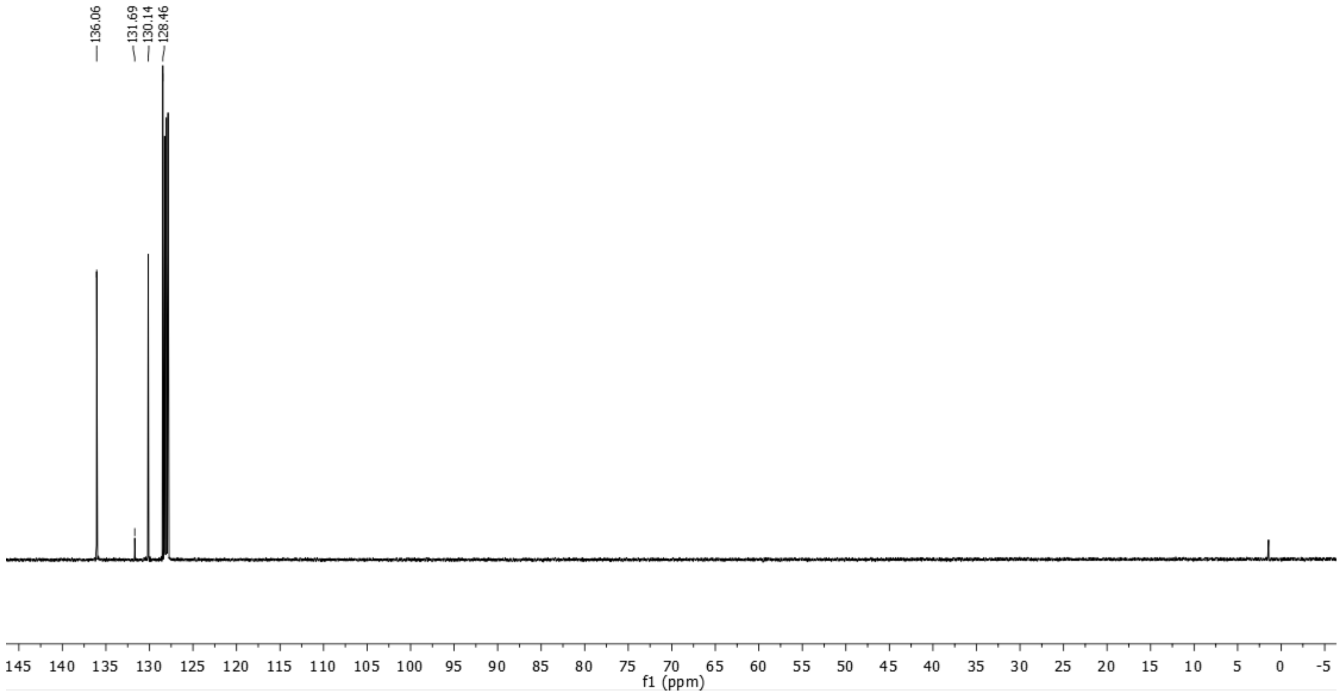


**Figure S30:** $^{13}C\{^{1}H\}$ NMR spectrum of diphenyl(silane-d$_2$).

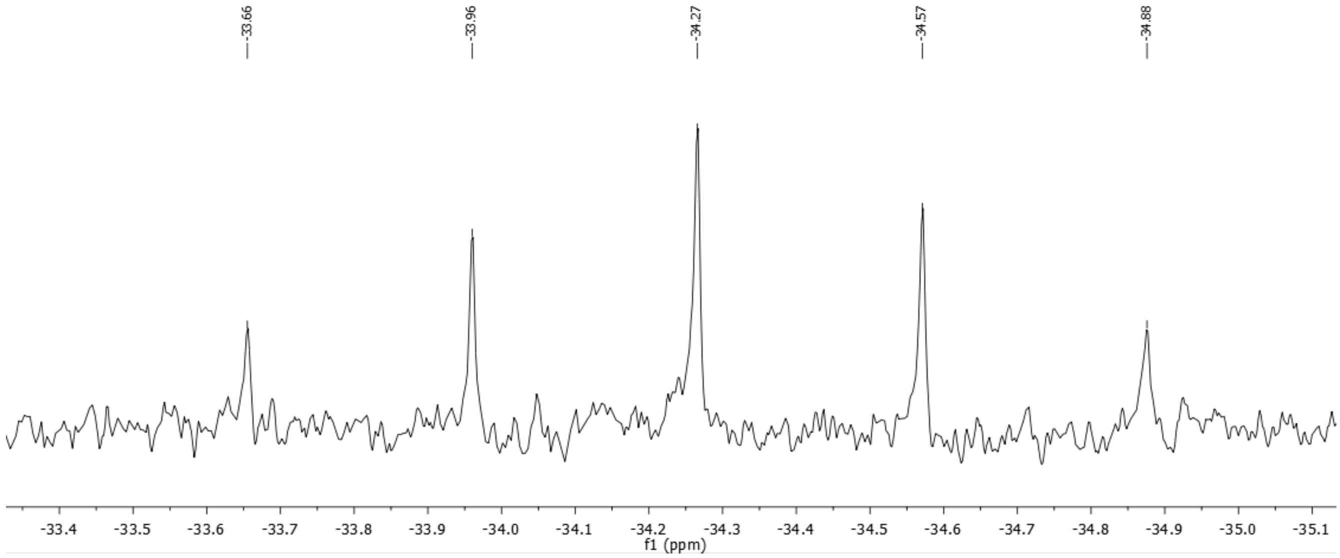


**Figure S31:** $^{29}Si$ NMR spectrum of diphenyl(silane-d$_2$).

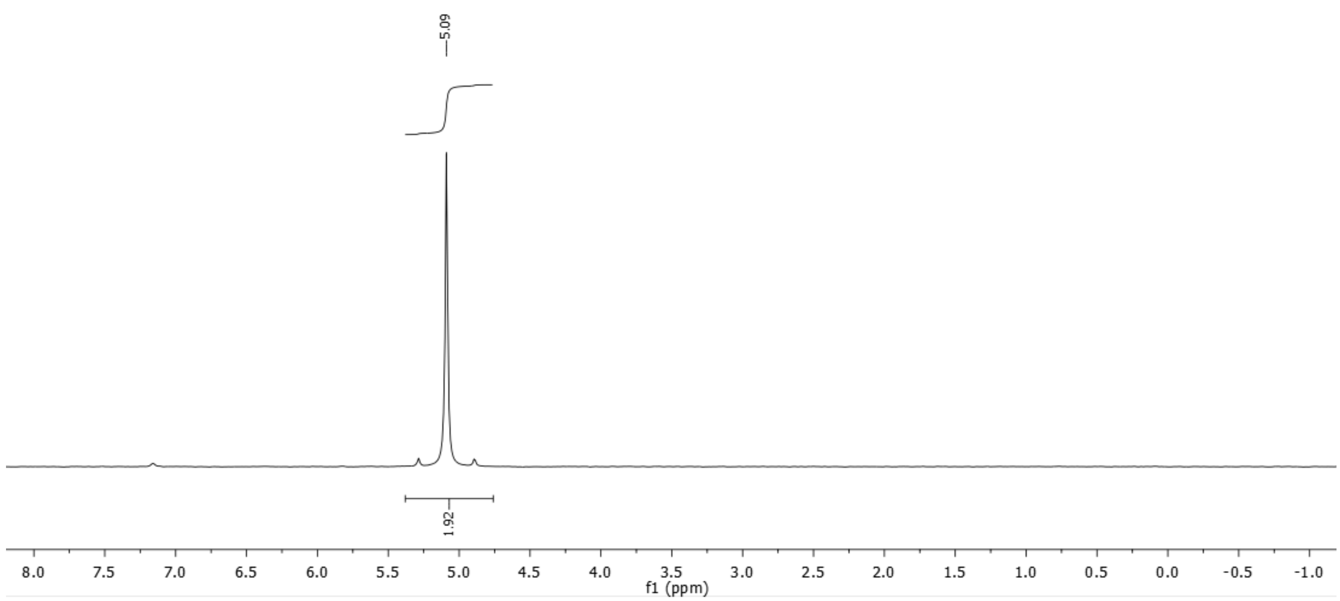


**Figure S32:** $^{2}H$ NMR spectrum of diphenyl(silane-$d_2$).

**0.25 mmol in Pentane**

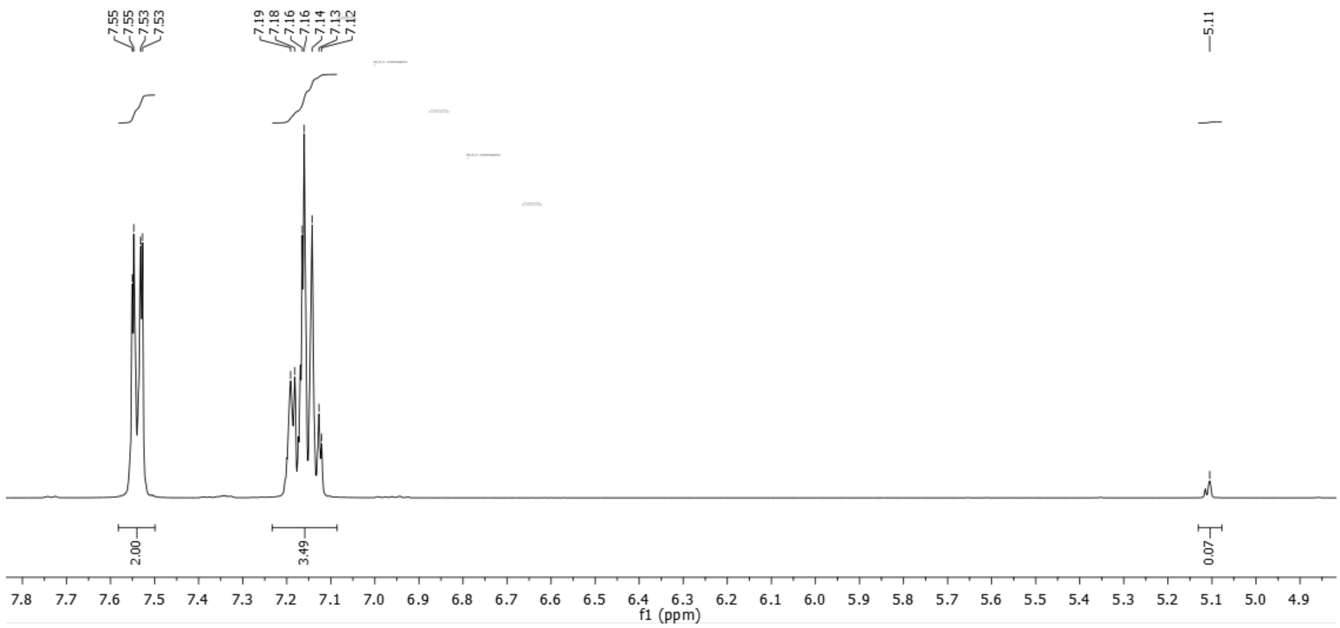


**Figure S33:** $^{1}H$ NMR spectrum of diphenyl(silane-$d_2$).

**Diethyl(silane-$d_2$), 2d**

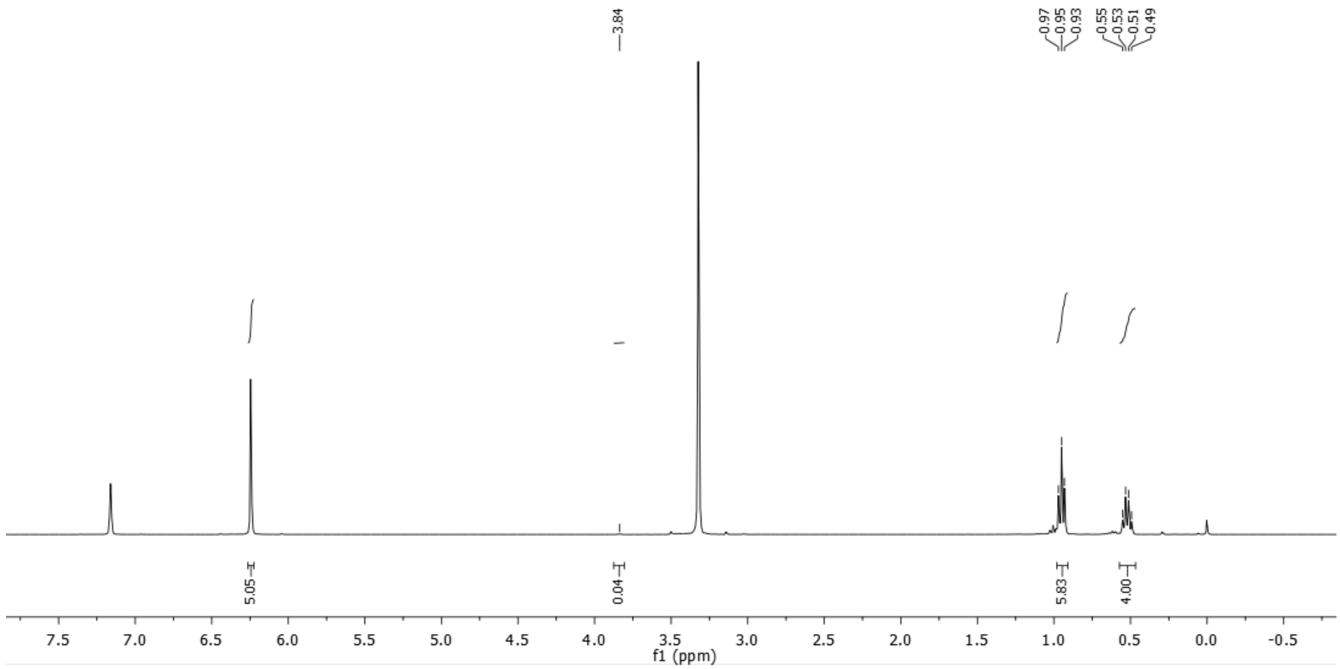


**Figure S34:** $^{1}$H NMR spectrum of diethyl(silane-d$_2$) and 1,3,5-trimethoxybenzene.

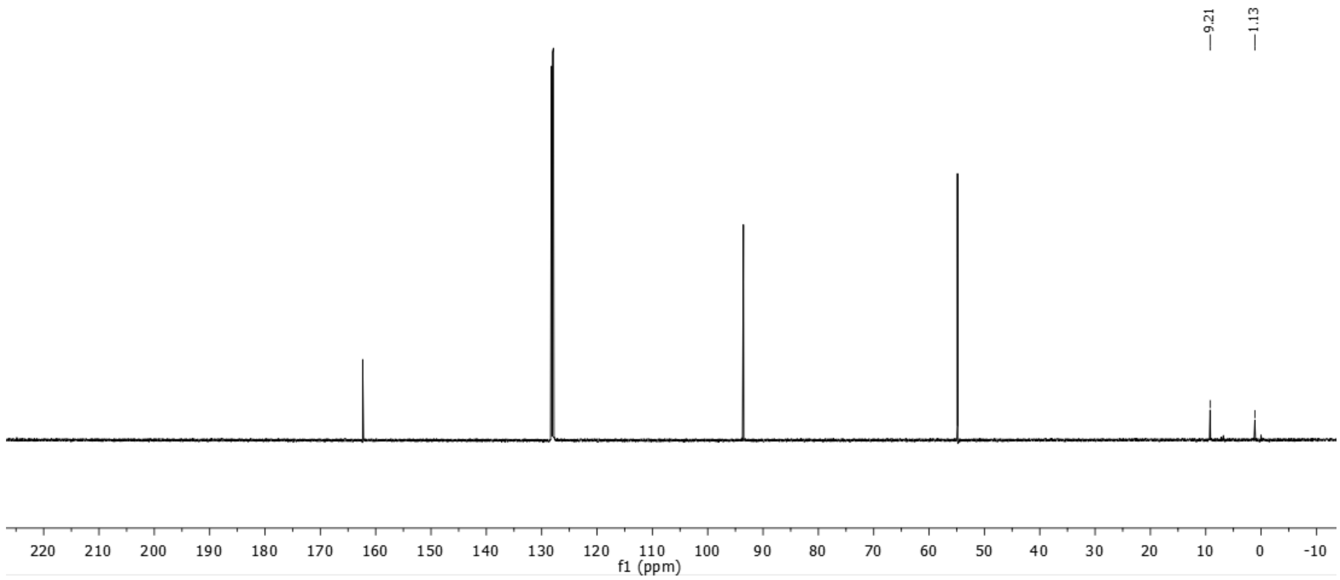


**Figure S35:** $^{13}$C{$^{1}$H} NMR spectrum of diethyl(silane-d$_2$) and 1,3,5-trimethoxybenzene.

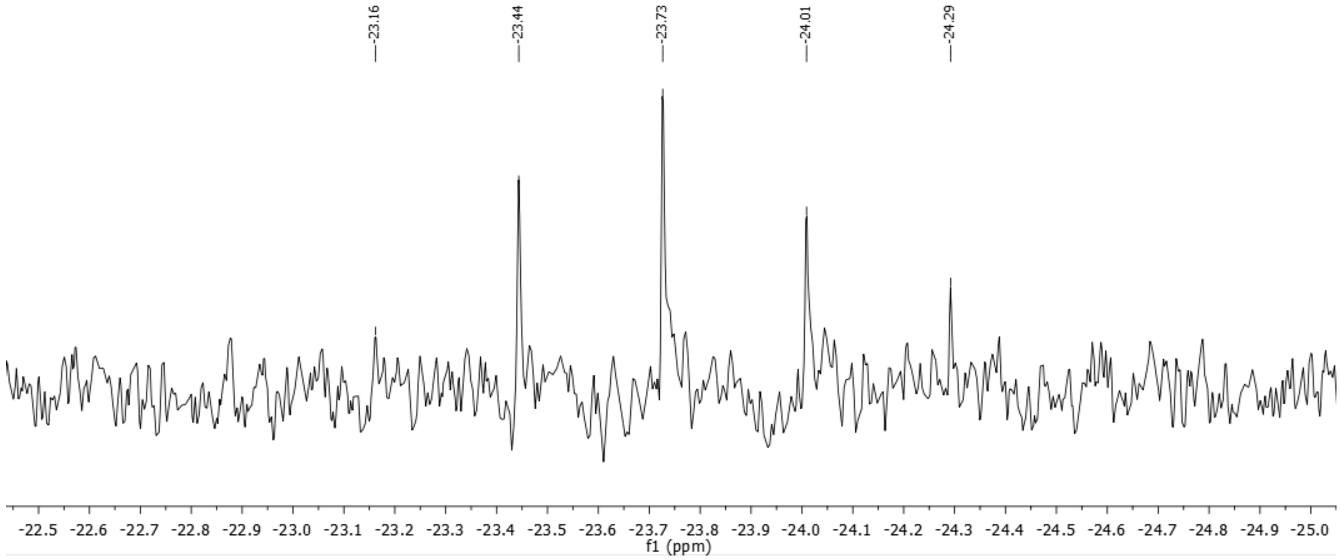


**Figure S36:** $^{29}$Si NMR spectrum of diethyl(silane-d$_2$) and 1,3,5-trimethoxybenzene.

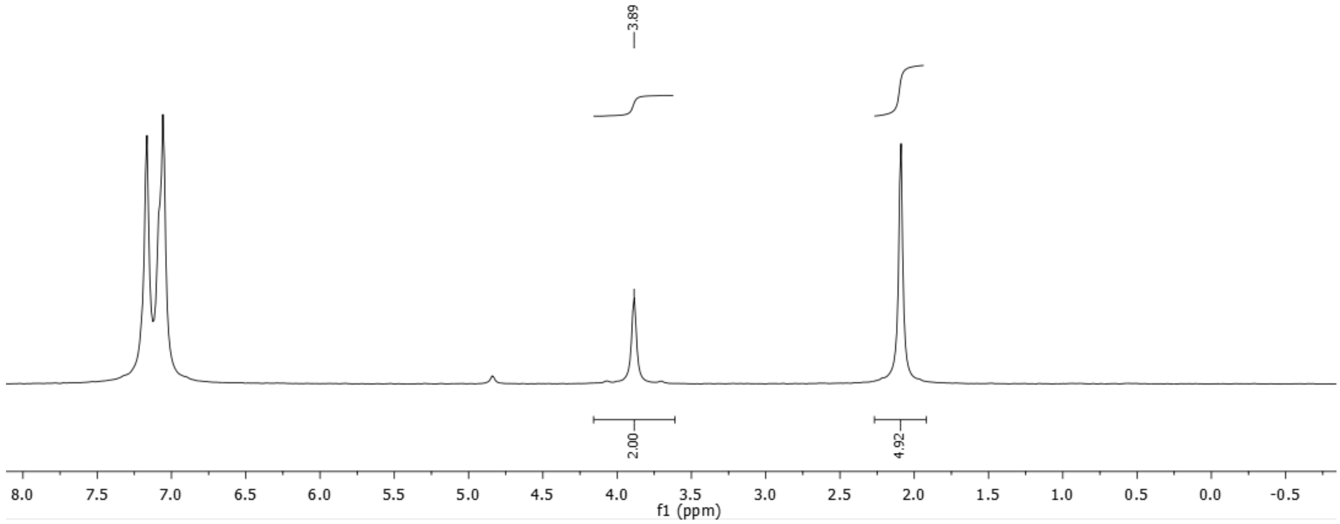


**Figure S37:** $^2$H NMR spectrum of diethyl(silane-d$_2$) and toluene-d$_8$.

**Hexyl(silane-d$_3$), 2e**

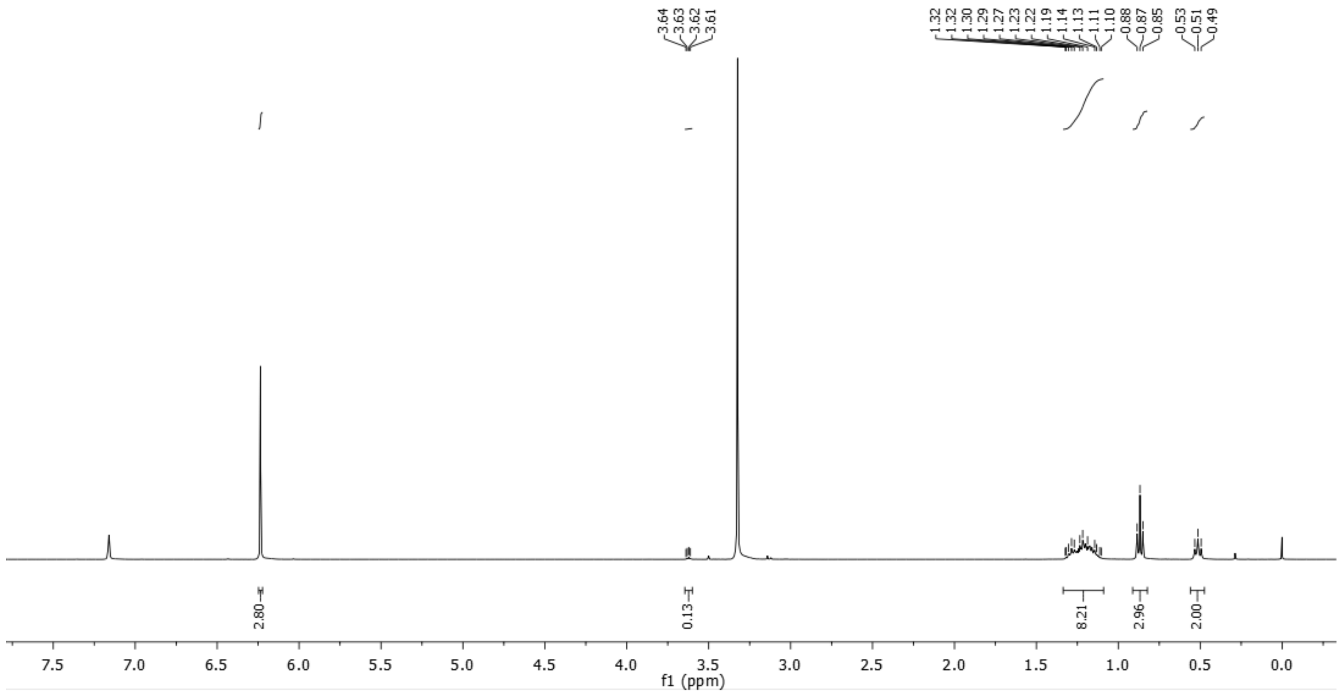


**Figure S38:** $^{1}$H NMR spectrum of hexyl(silane-d$_3$) and 1,3,5-trimethoxybenzene.

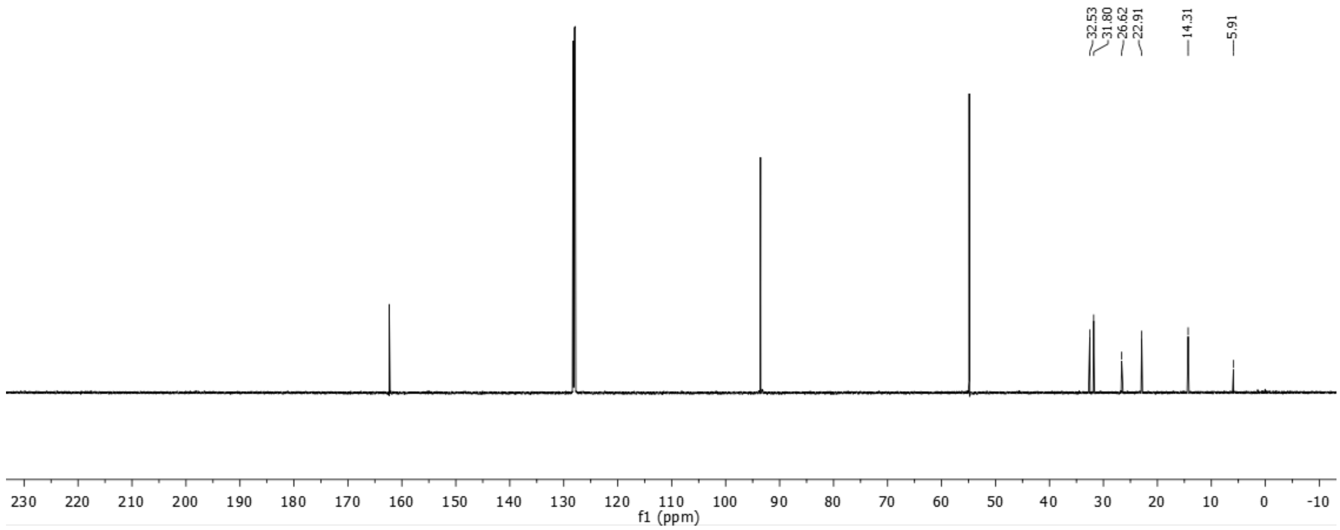


**Figure S39:** $^{13}$C{$^{1}$H} NMR spectrum of hexyl(silane-d$_3$) and 1,3,5-trimethoxybenzene.

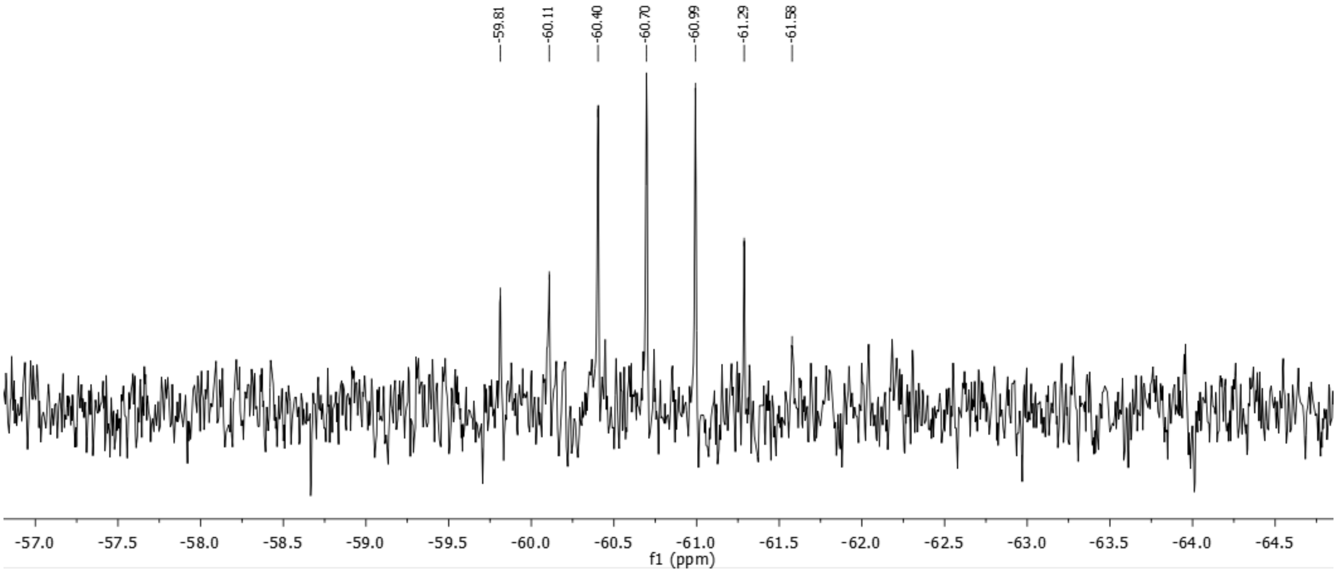


**Figure S40:** $^{29}$Si NMR spectrum of hexyl(silane-d$_3$) and 1,3,5-trimethoxybenzene.

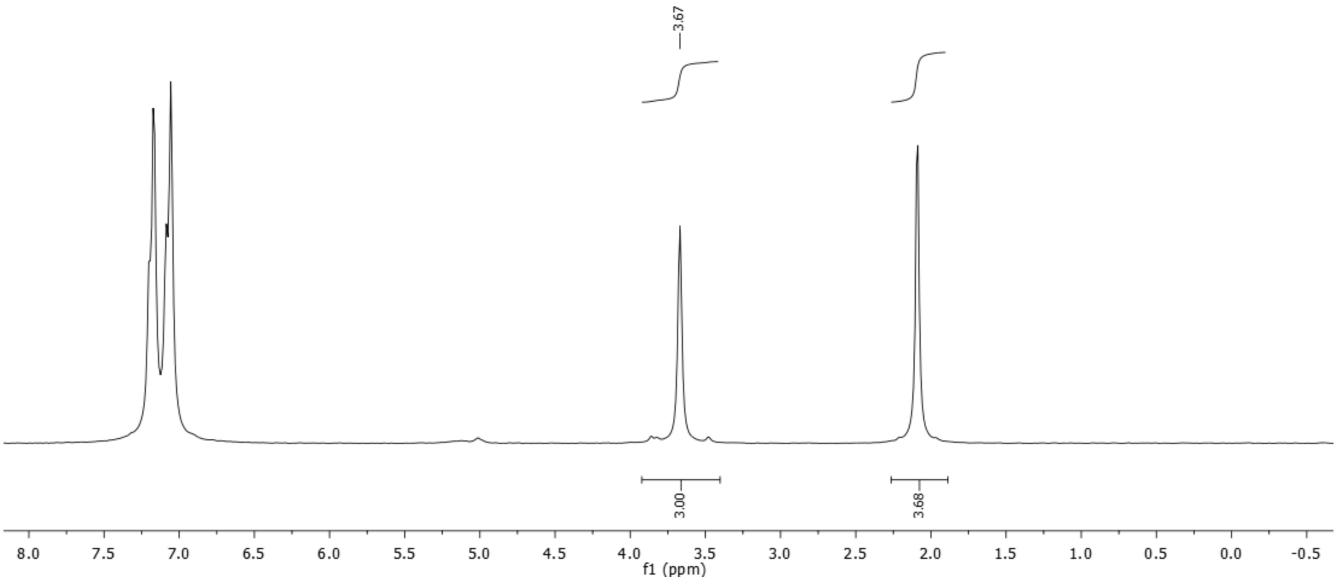


**Figure S41:** $^{2}$H NMR spectrum of hexyl(silane-d$_3$) and toluene-d$_8$.

**Octadecyl(silane-d$_3$), 2f**

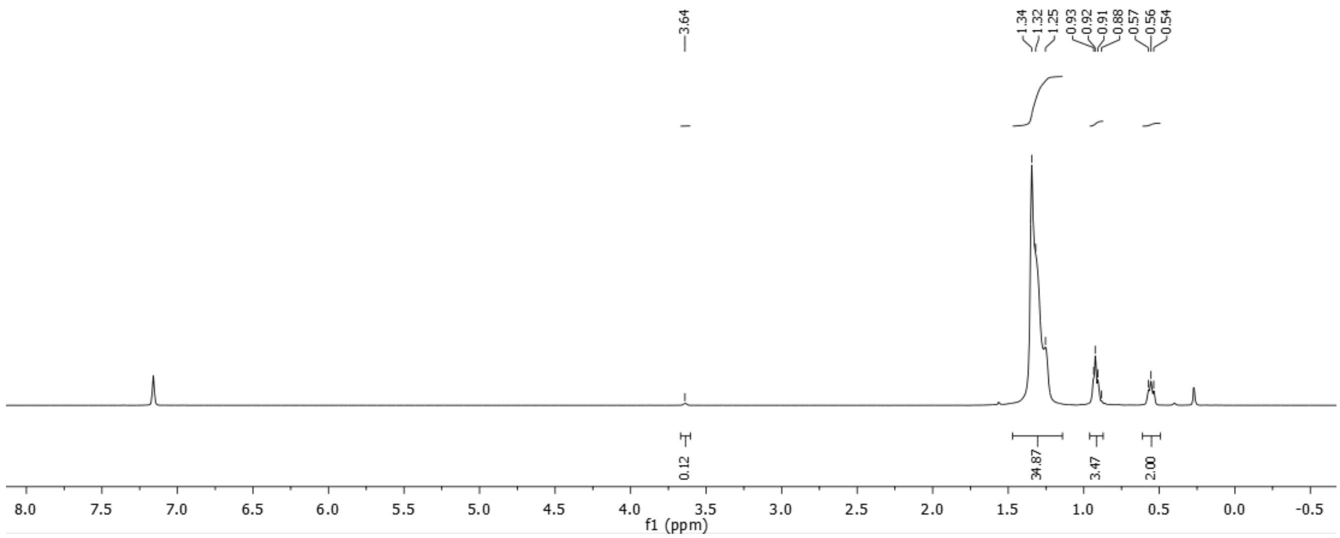


**Figure S42:** $^{1}$H NMR spectrum of octadecyl(silane-d$_3$).

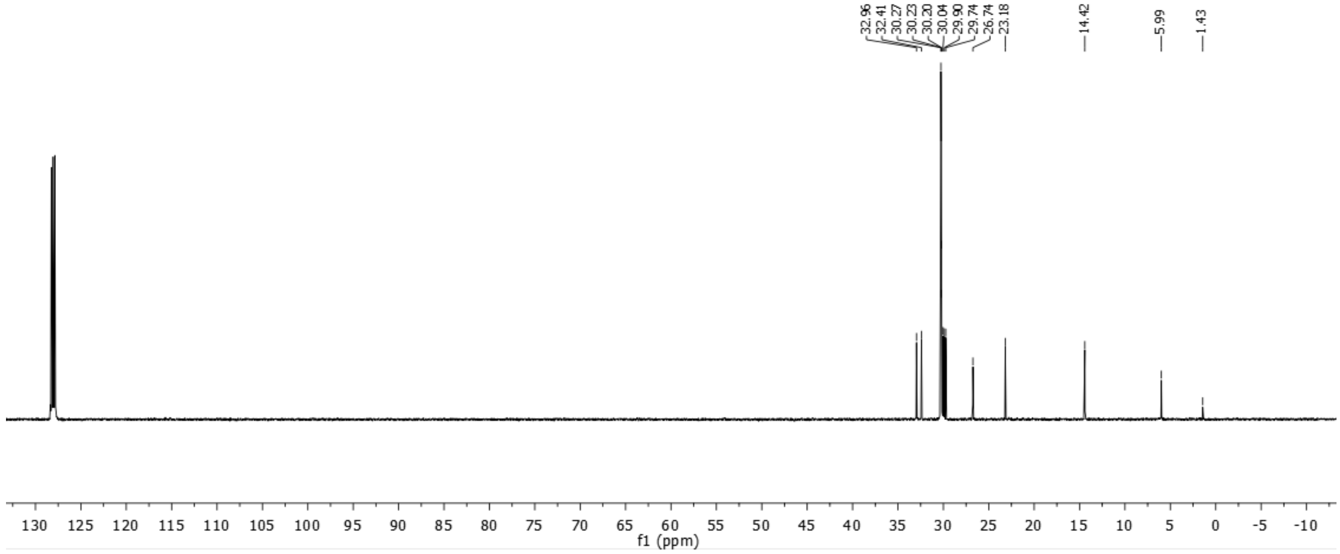


**Figure S43:** $^{13}$C{$^{1}$H} NMR spectrum of octadecyl(silane-d$_3$).

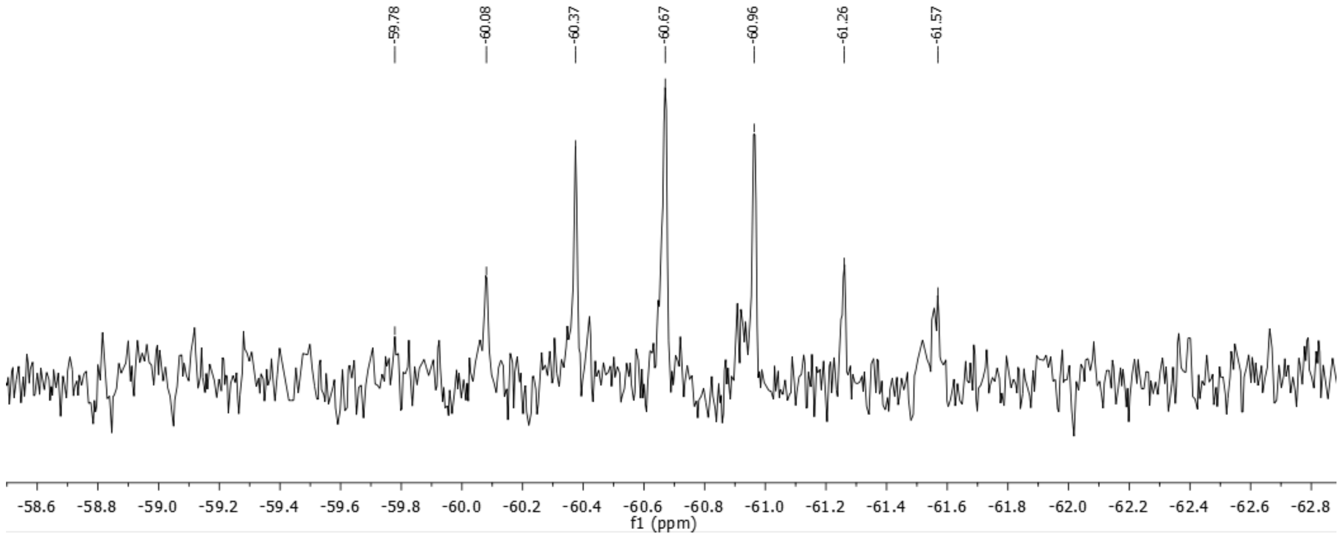


**Figure S44:** $^{29}$Si NMR spectrum of octadecyl(silane-d$_3$).

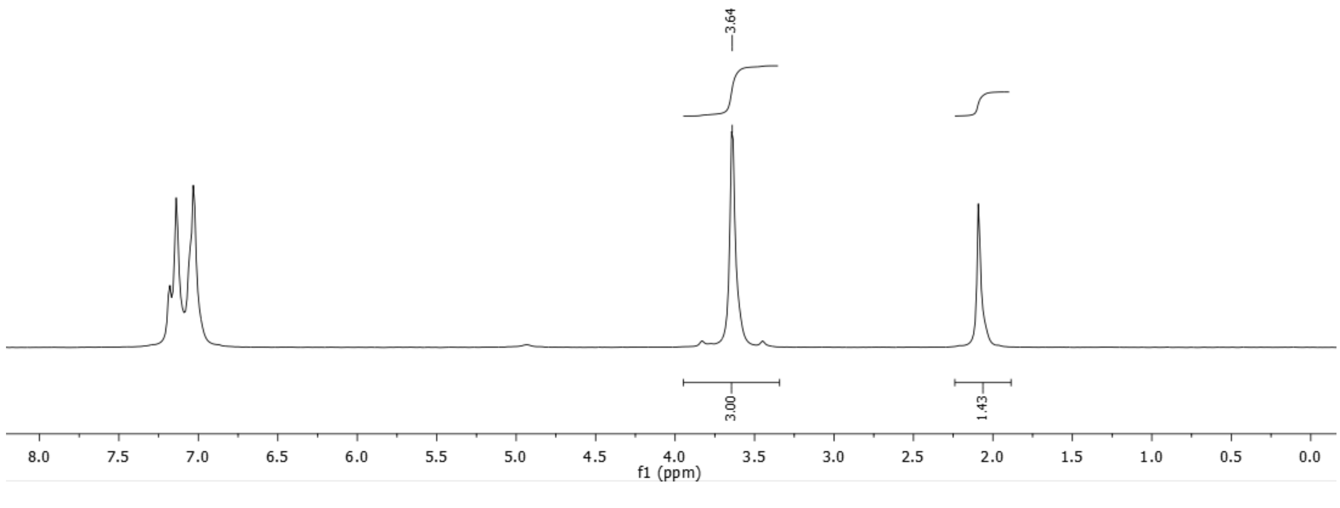


**Figure S45:** $^{2}$H NMR spectrum of octadecyl(silane-d$_3$) and toluene-d$_8$.

**1,1,3,3-Tetramethyldi(siloxane-d), 2g**

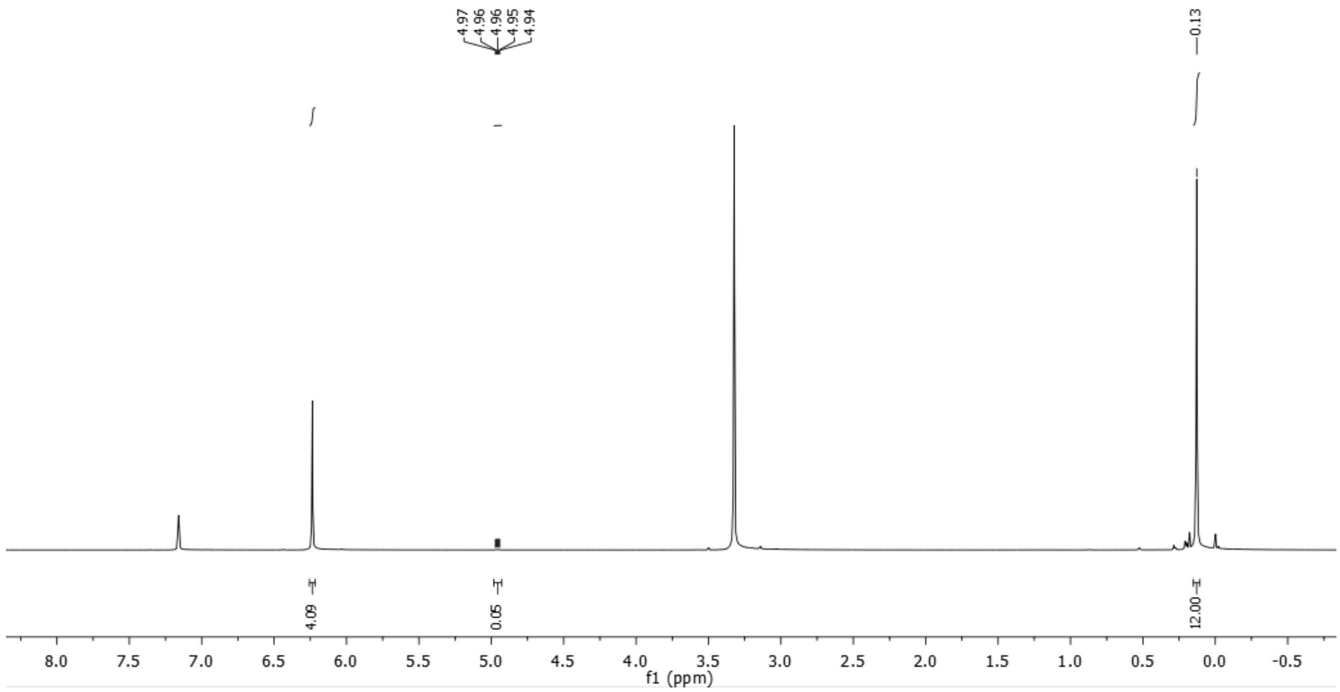


**Figure S46:** $^{1}H$ NMR spectrum of 1,1,3,3-tetramethyldi(siloxane-d) and 1,3,5-trimethoxybenzene.

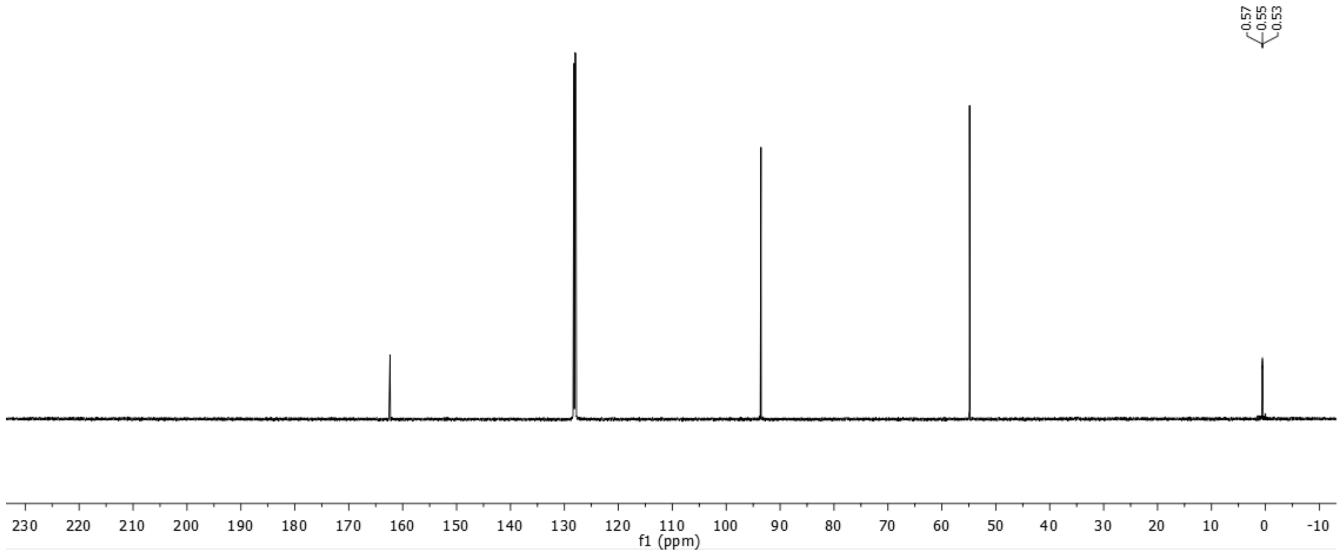


**Figure S47:** $^{13}C\{^{1}H\}$ NMR spectrum of 1,1,3,3-tetramethyldi(siloxane-d) and 1,3,5-trimethoxybenzene.

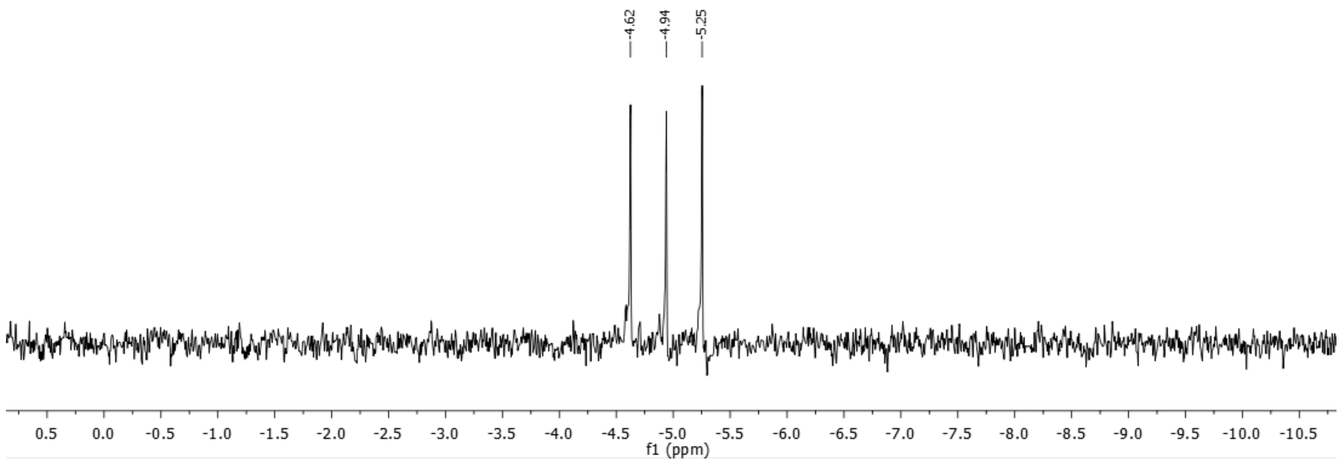


**Figure S48:** $^{29}$Si NMR spectrum of 1,1,3,3-tetramethyldi(siloxane-d) and 1,3,5-trimethoxybenzene.

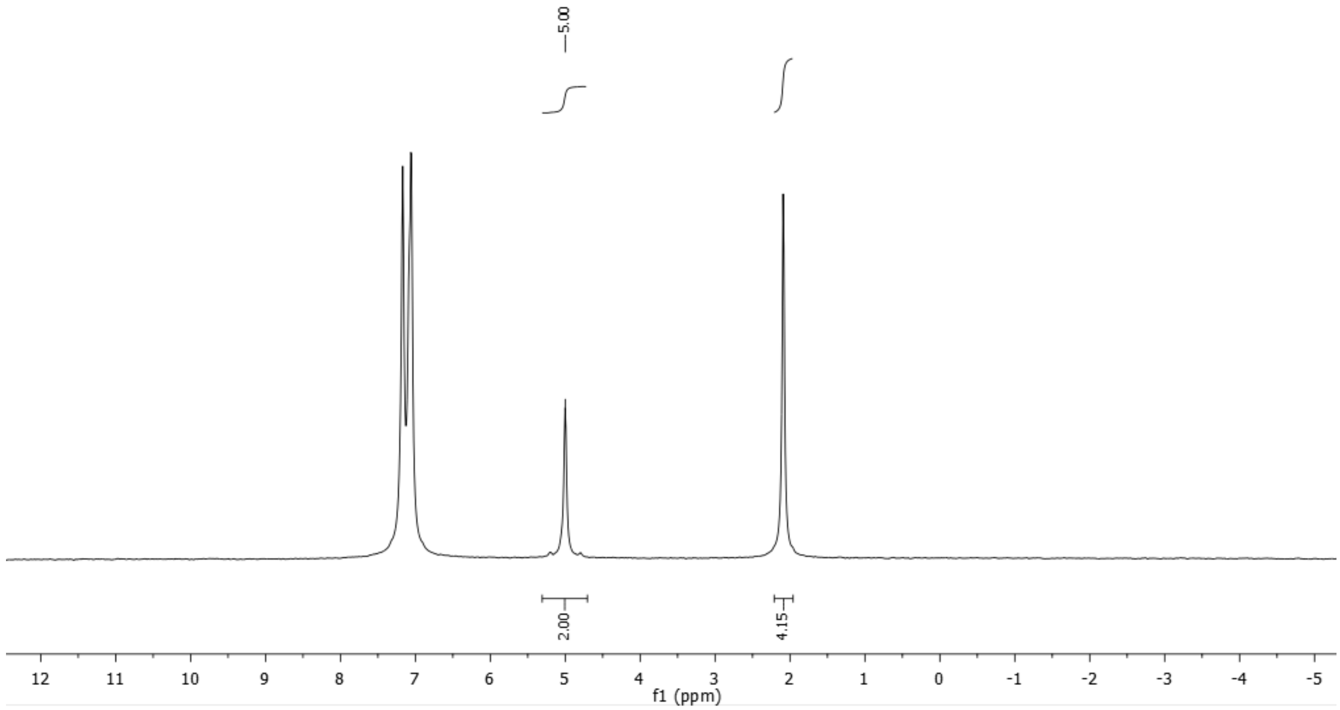


**Figure S49:** $^{2}$H NMR spectrum of 1,1,3,3-tetramethyldi(siloxane-d) and toluene-$d_8$.

**4,4,5,5-Tetramethyl-1,3,2-dioxa(borolane-d), 2h**

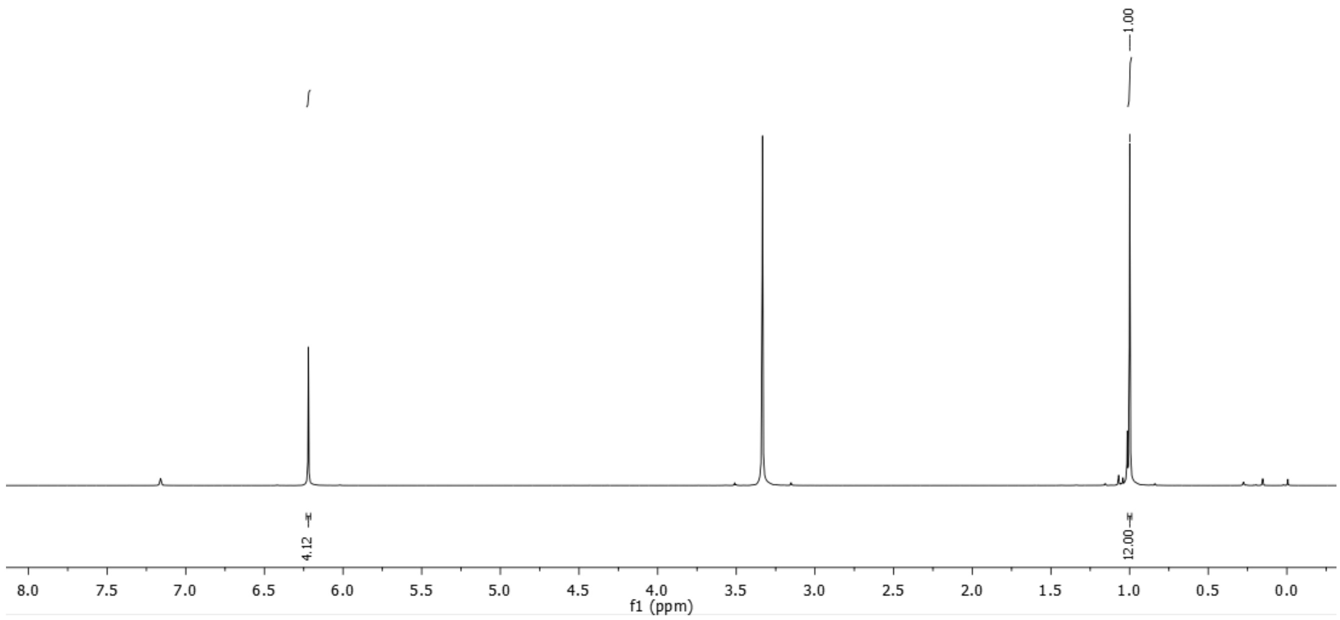


**Figure S50:** $^{1}H$ NMR spectrum of 4,4,5,5-tetramethyl-1,3,2-dioxa(borolane-d) and 1,3,5-trimethoxybenzene.

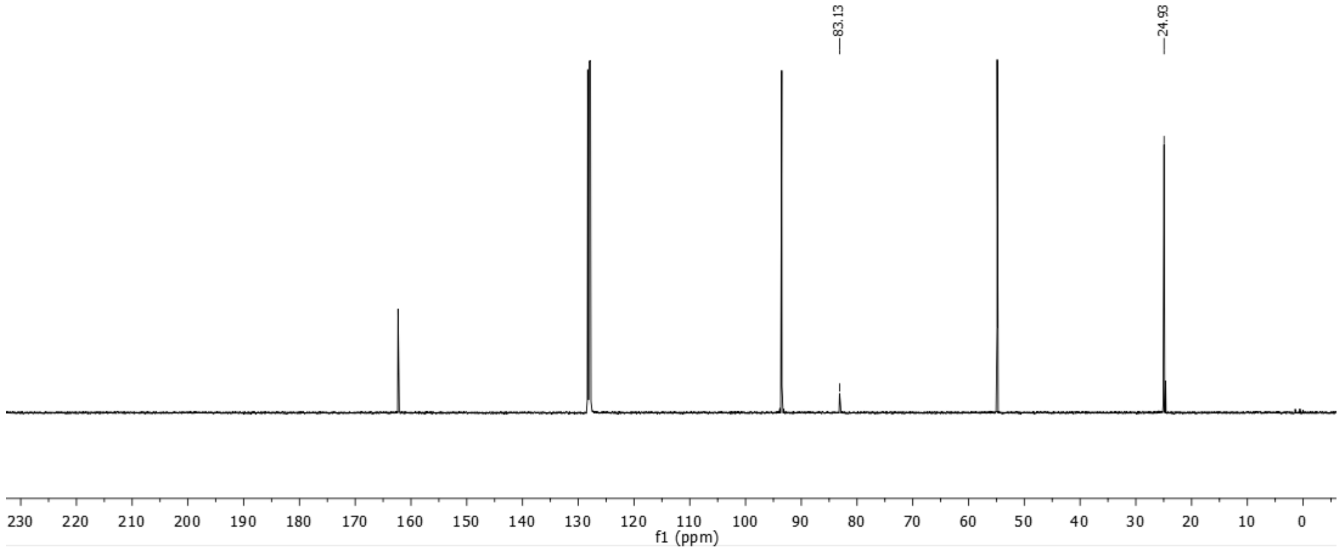


**Figure S51:** $^{13}C\{^{1}H\}$ NMR spectrum of 4,4,5,5-tetramethyl-1,3,2-dioxa(borolane-d) and 1,3,5-trimethoxybenzene.

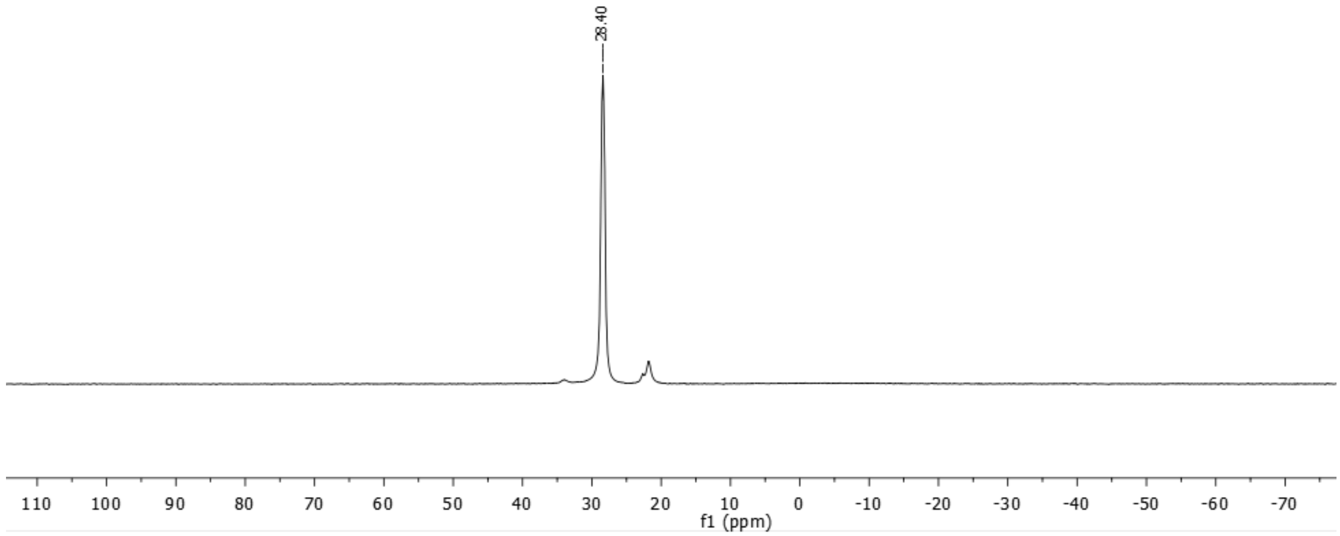

**Figure S52:** $^{11}$B NMR spectrum of 4,4,5,5-tetramethyl-1,3,2-dioxa(borolane-d) and 1,3,5-trimethoxybenzene.

**(Propyl-2-d)benzene, 2i**

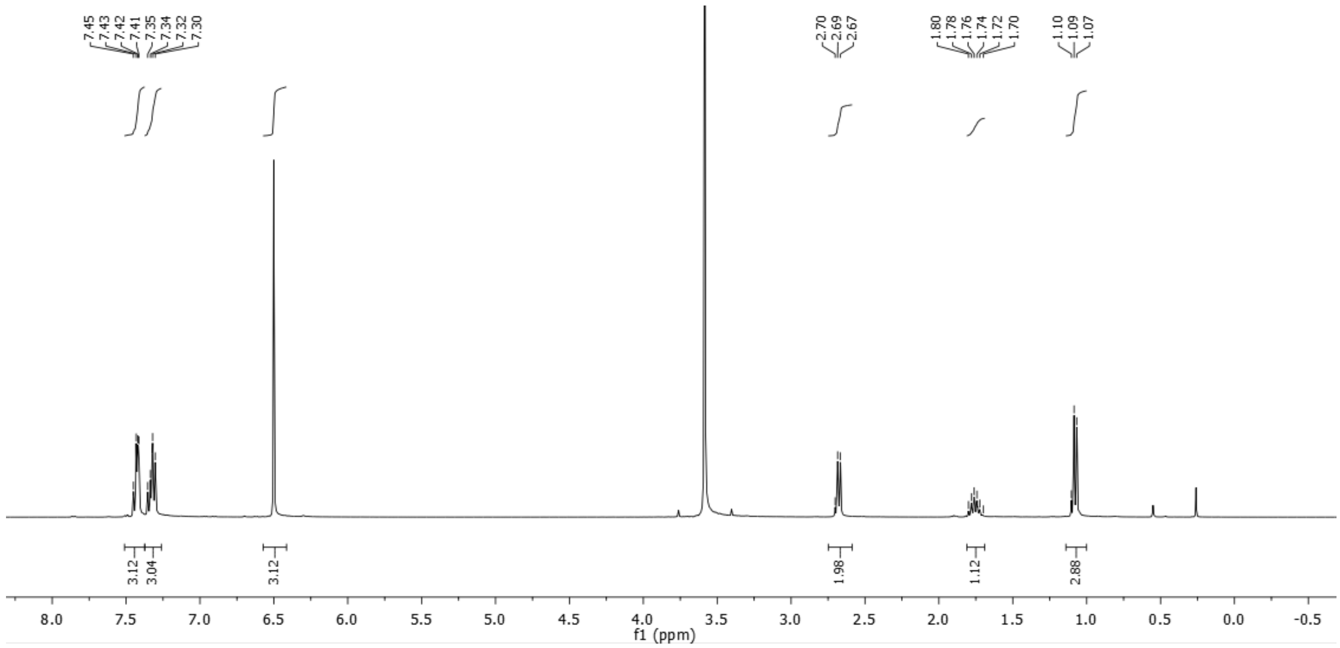

**Figure S53:** $^{1}$H NMR spectrum of (propyl-2-d)benzene and 1,3,5-trimethoxybenzene.

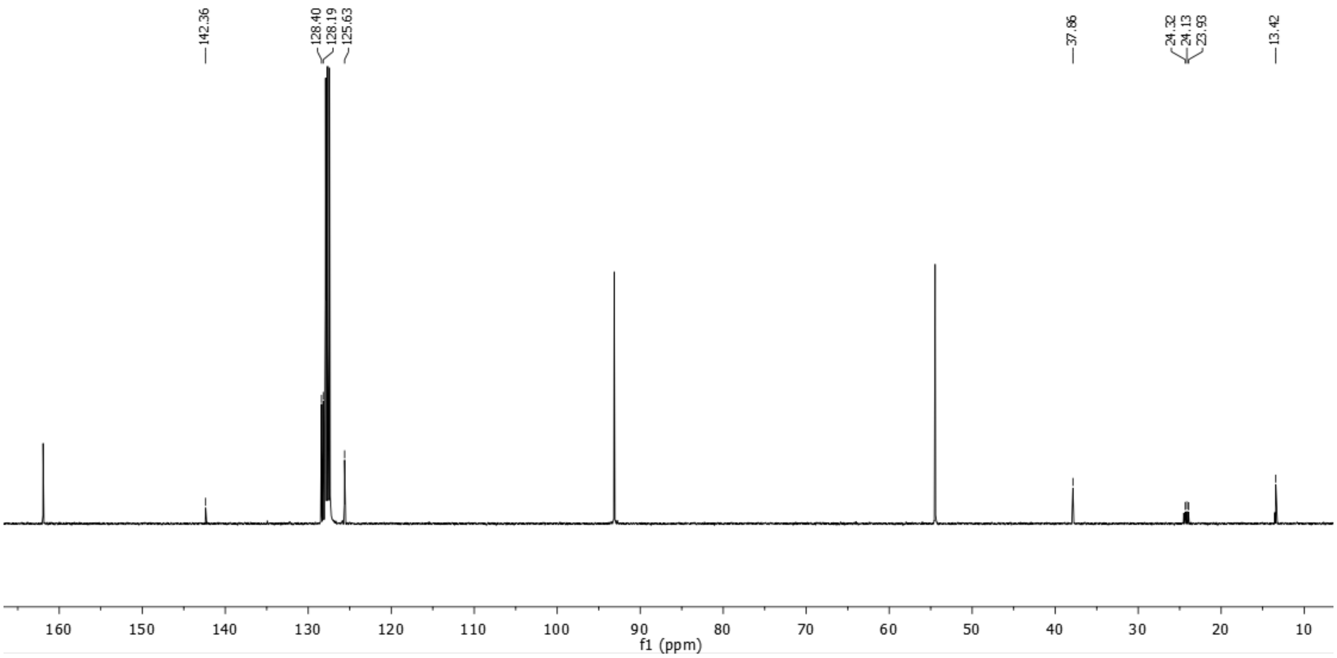


**Figure S54:** $^{13}C\{^1H\}$ NMR spectrum of (propyl-2-d)benzene and 1,3,5-trimethoxybenzene.

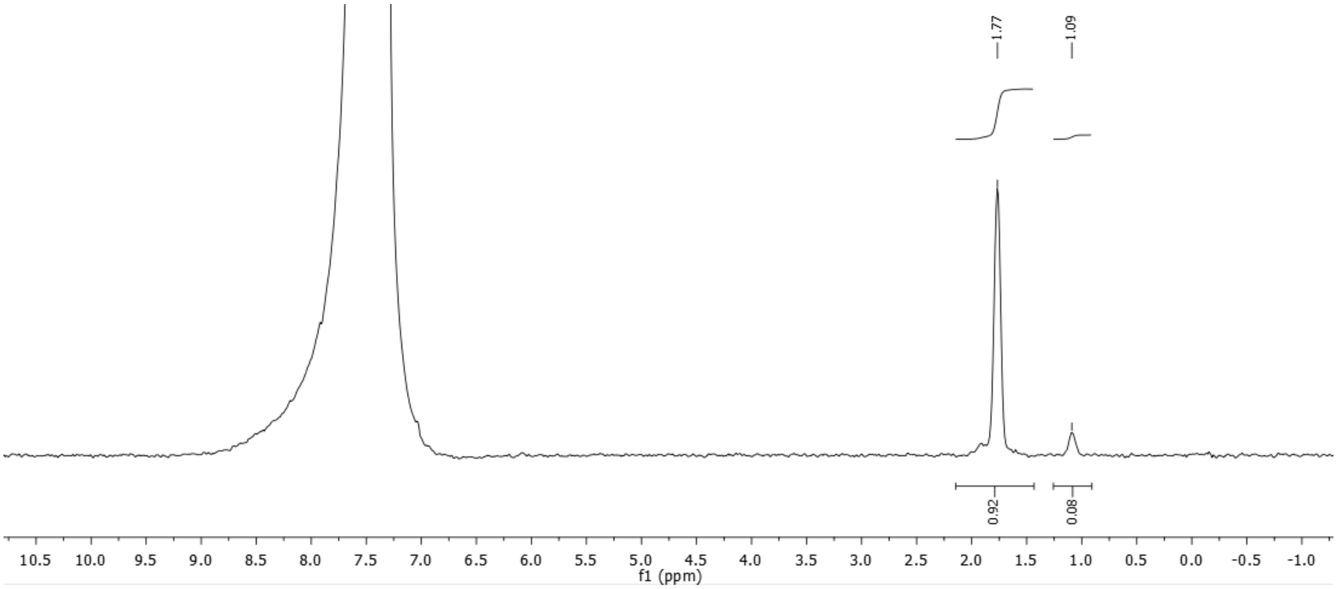


**Figure S55:** $^2H$ NMR spectrum of (propyl-2-d)benzene.

**Methylphenylsilane, 2a-H**

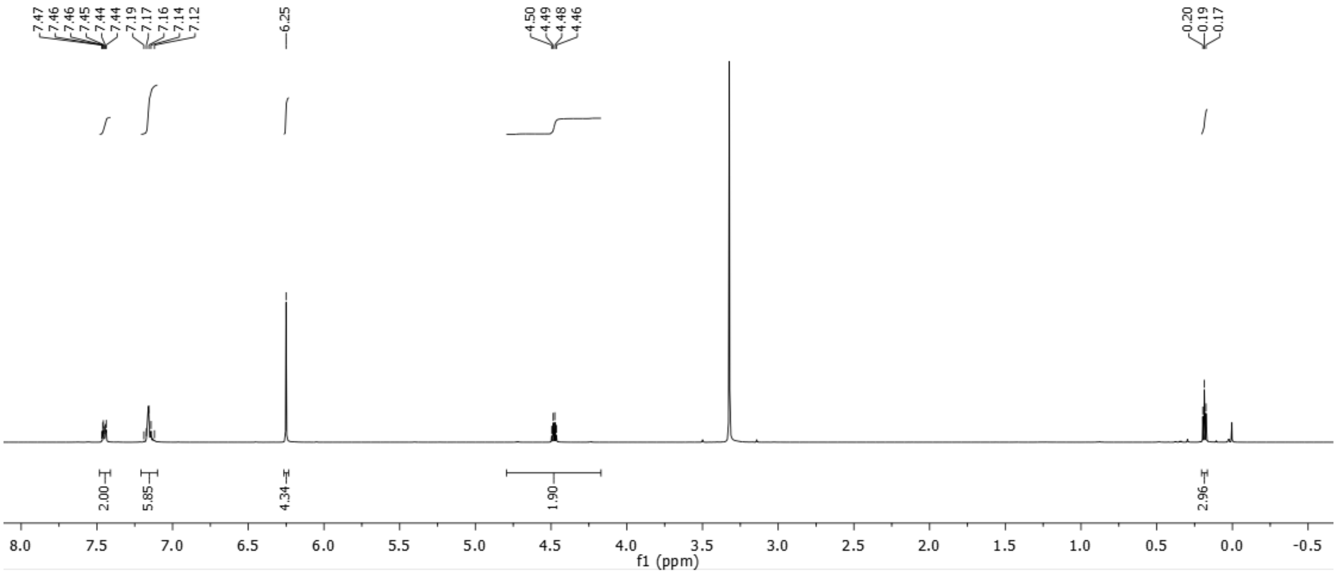

**Figure S56:** $^{1}$H NMR spectrum of methylphenylsilane and 1,3,5-trimethoxybenzene.

# 11 Complex and Reagent Spectra

### $^{2,6\text{-dimethylphenyl}}\beta$-Diketiminate

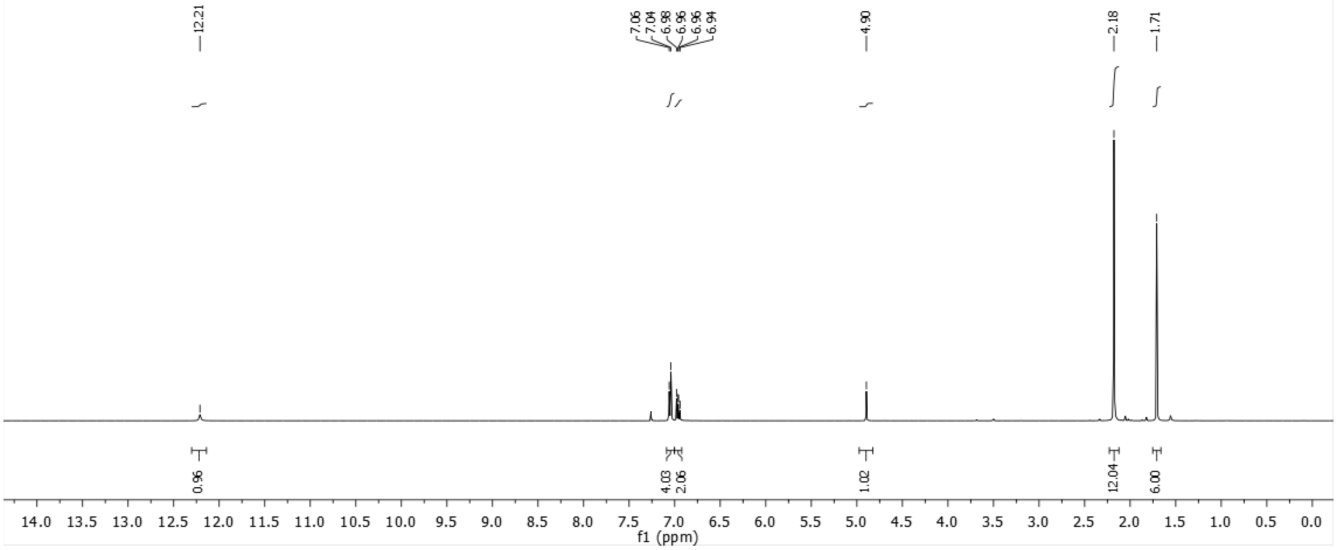

**Figure S57:** $^{1}$H NMR spectrum of $^{2,6\text{-dimethylphenyl}}\beta$-diketiminate.

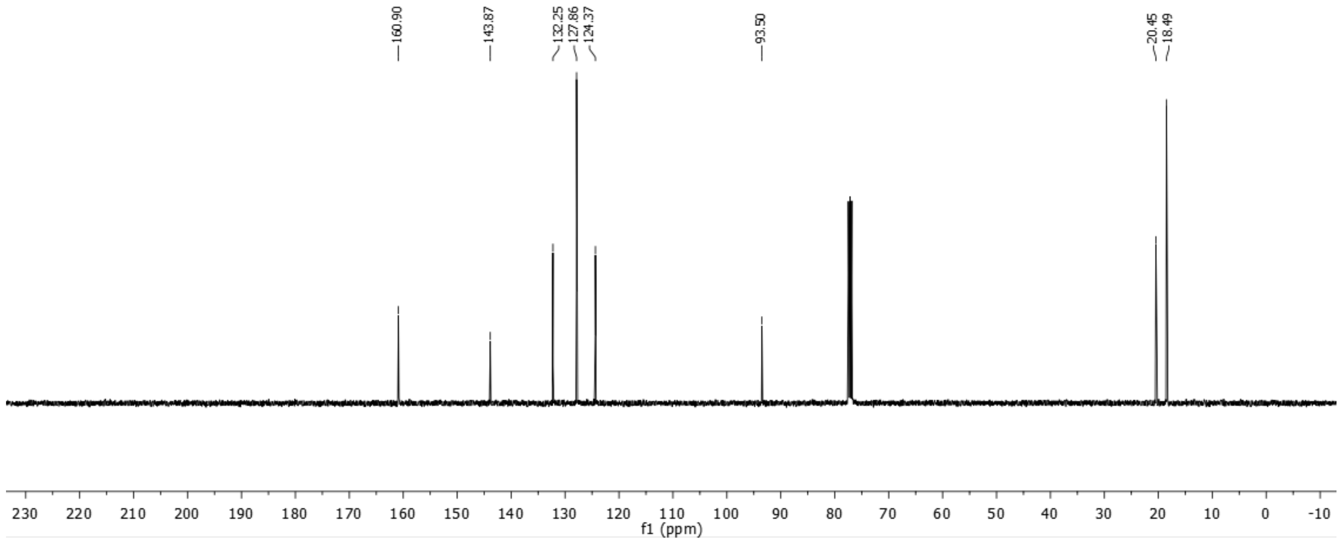


**Figure S58:** $^{13}C\{^1H\}$ NMR spectrum of $^{2,6\text{-dimethylphenyl}}\beta$-diketiminate.

### $^{2,6\text{-dimethylphenyl}}$BDKFe($CH_2$TMS), 1b

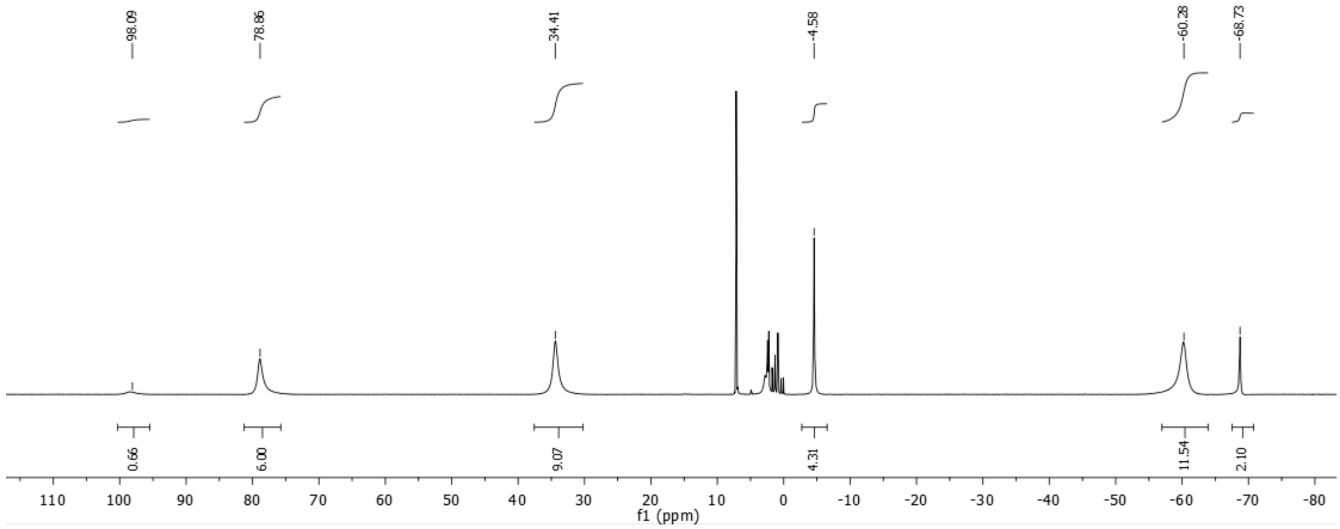


**Figure S59:** $^1H$ NMR spectrum of $^{2,6\text{-dimethylphenyl}}$BDKFe($CH_2$TMS).

### $^{2,6\text{-dipp}}$BDKFe-($\mu$-H)$_2$-9-BBN, 3a

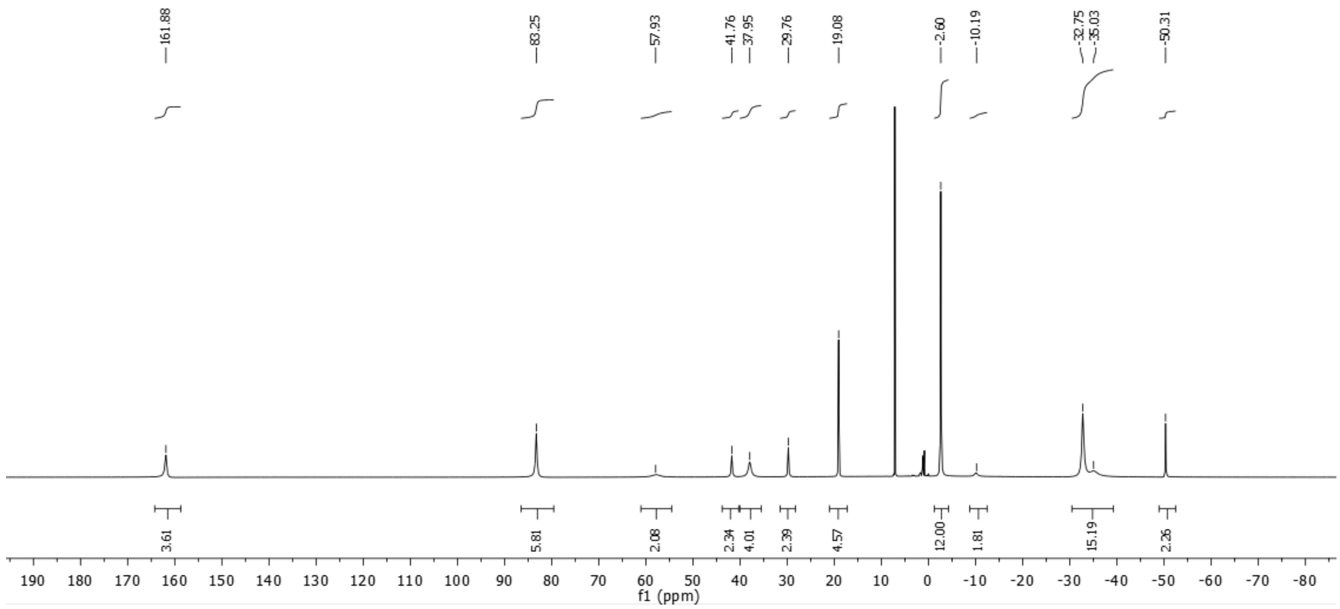


**Figure S60:** $^{1}H$ NMR spectrum of $^{2,6\text{-diisopropylphenyl}}$BDKFe-($\mu$-H)$_2$-9-BBN

**$^{2,6\text{-dmp}}$BDKFe-($\mu$-H)-9-BBN, 3b**

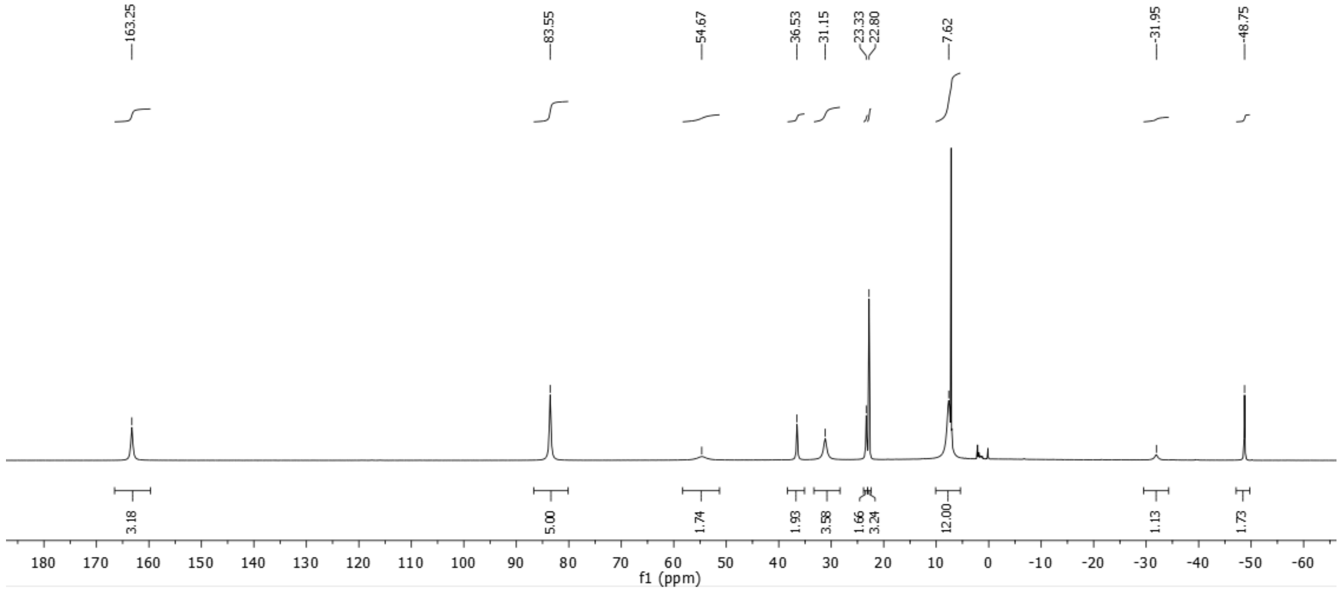


**Figure S61:** $^{1}H$ NMR spectrum of $^{2,6\text{-dimethylphenyl}}$BDKFe-($\mu$-H)$_2$-9-BBN.

# 12 Synthesised Substrate Spectra

**(4-(Dimethylsilyl)phenyl)methanol**

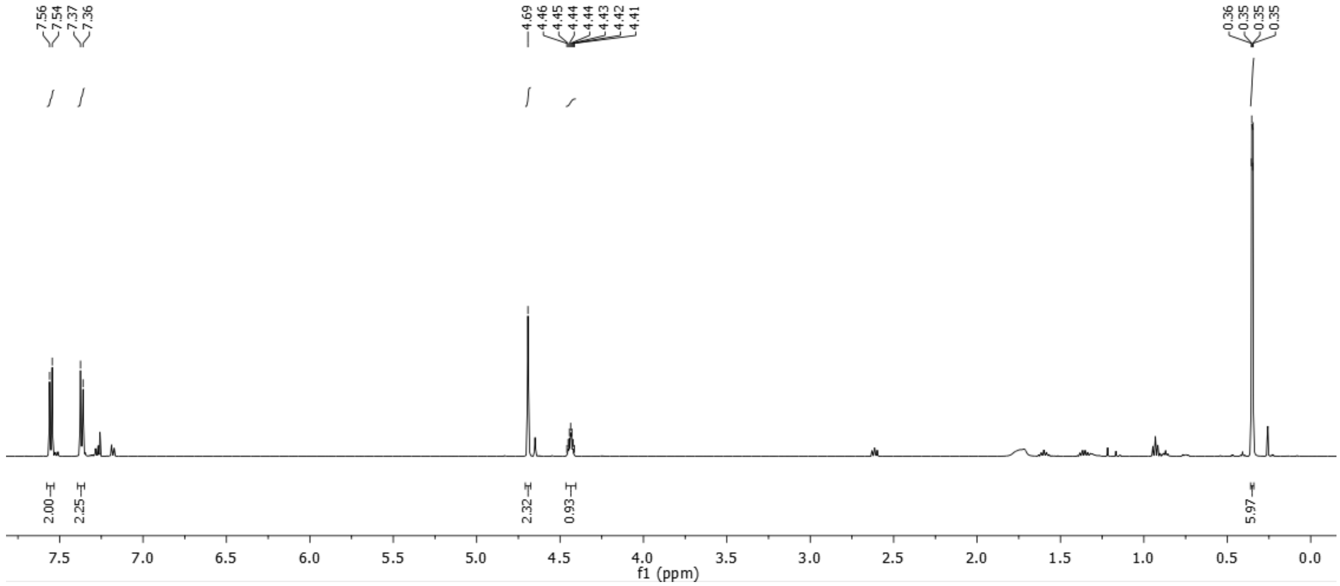


**Figure S62:** $^{1}$H NMR spectrum of (4-(dimethylsilyl)phenyl)methanol.

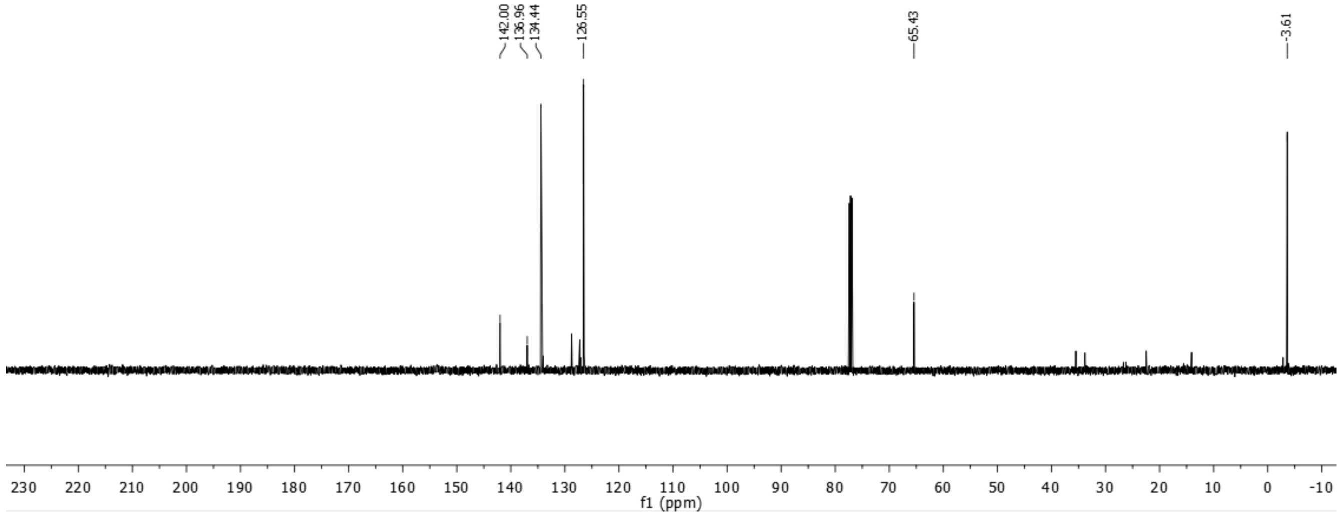


**Figure S63:** $^{13}C\{^{1}H\}$ NMR spectrum of (4-(dimethylsilyl)phenyl)methanol.

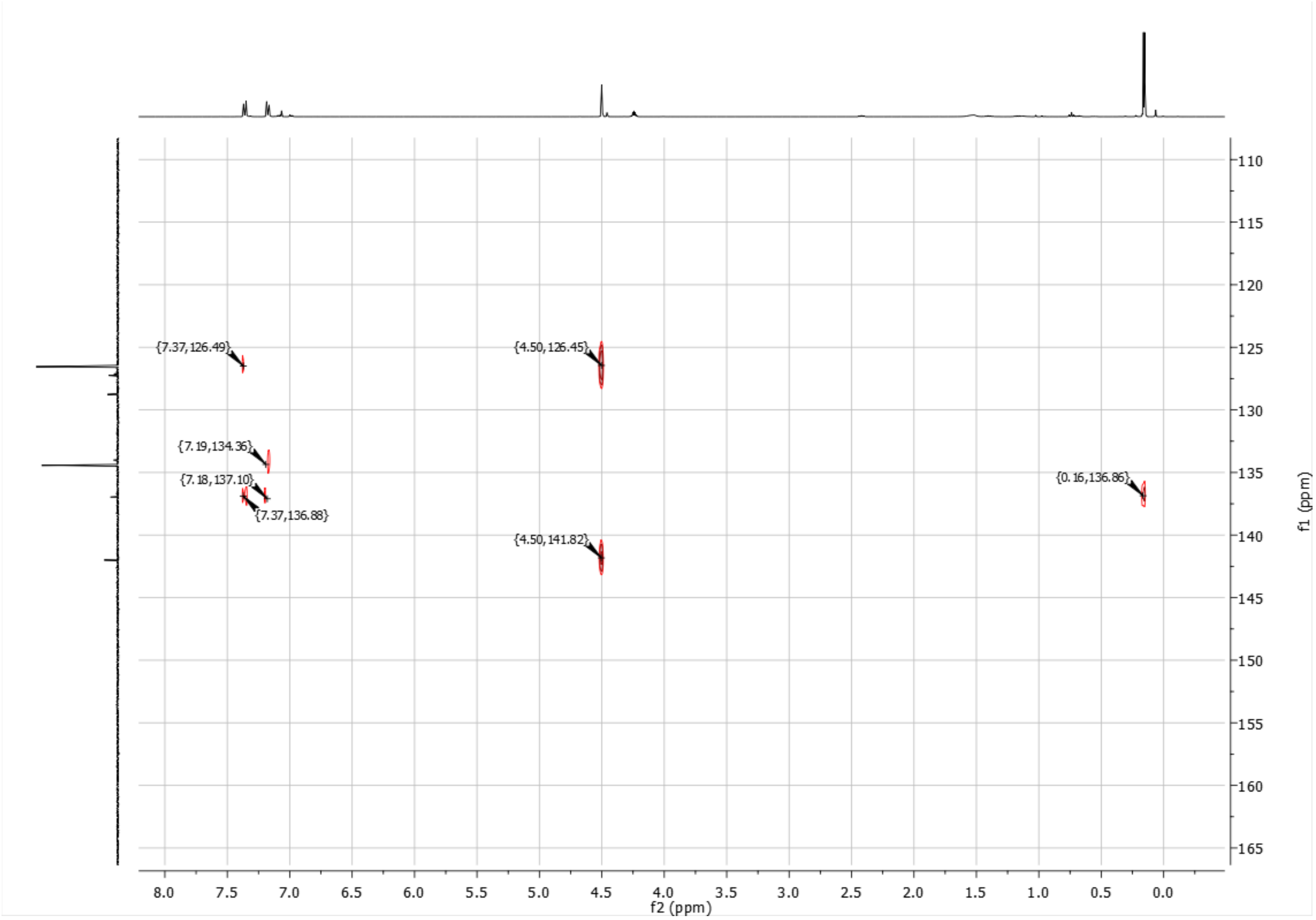


**Figure S64:** HMBC NMR spectrum of (4-(dimethylsilyl)phenyl)methanol.

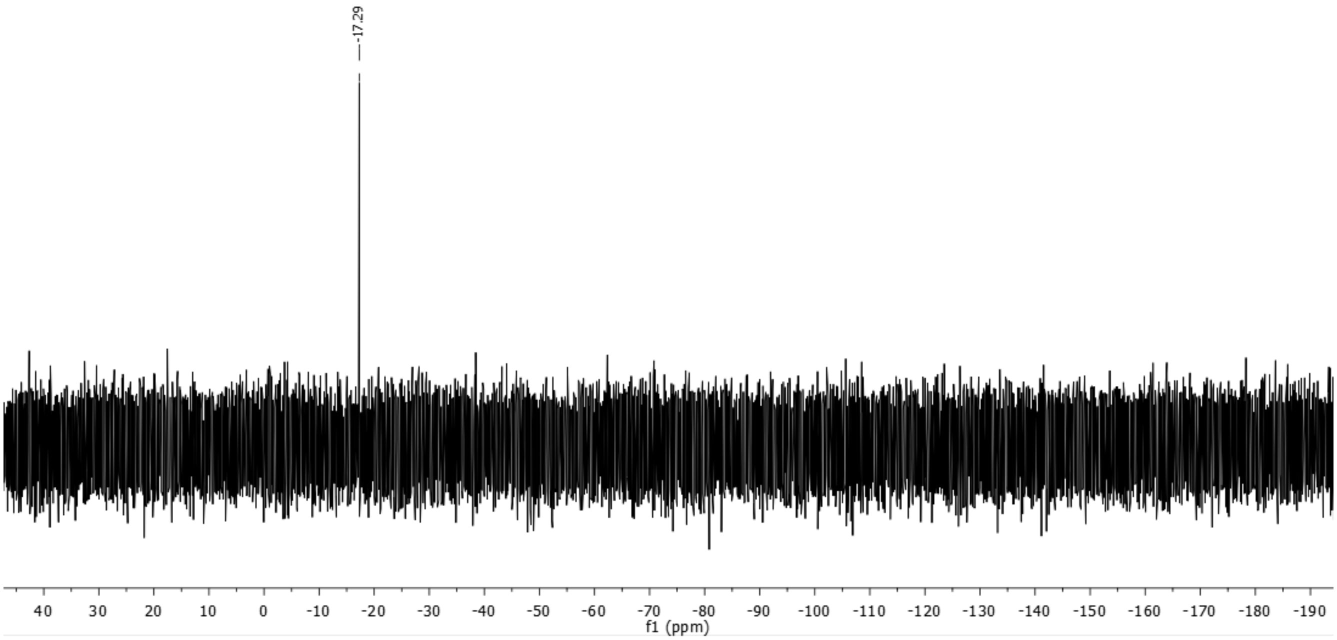


**Figure S65:** $^{29}$Si NMR spectrum of (4-(dimethylsilyl)phenyl)methanol.

**(4-(Chloromethyl)phenyl)dimethylsilane**

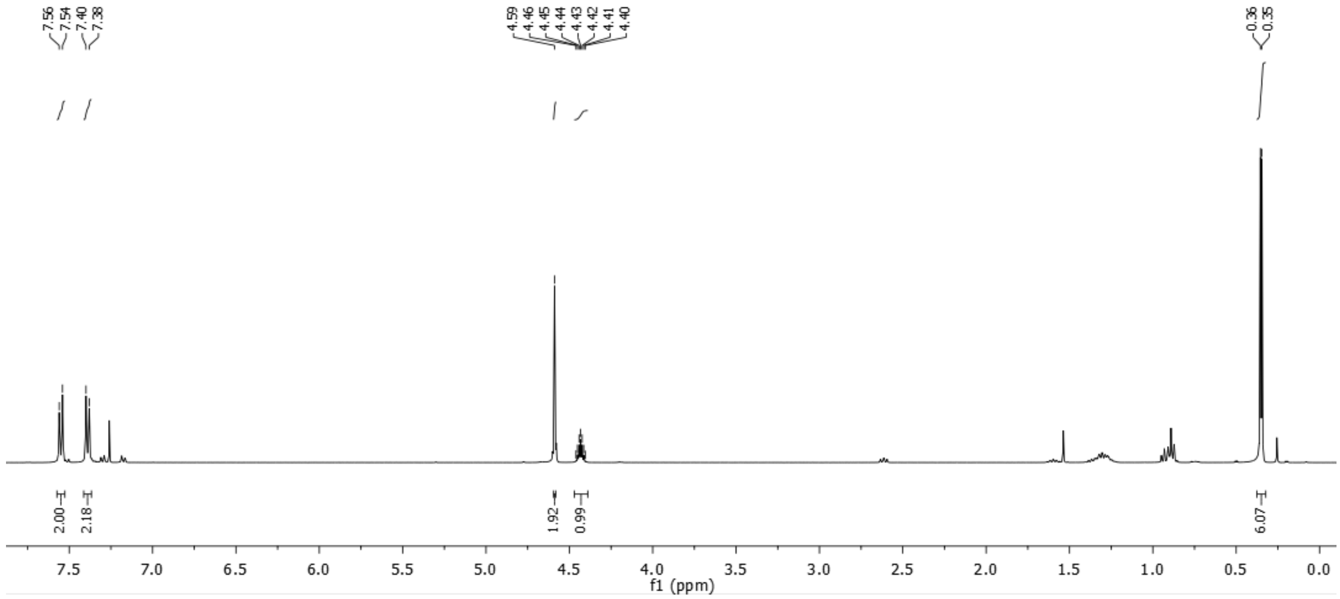


**Figure S66:** $^{1}$H NMR spectrum of (4-(chloromethyl)phenyl)dimethylsilane.

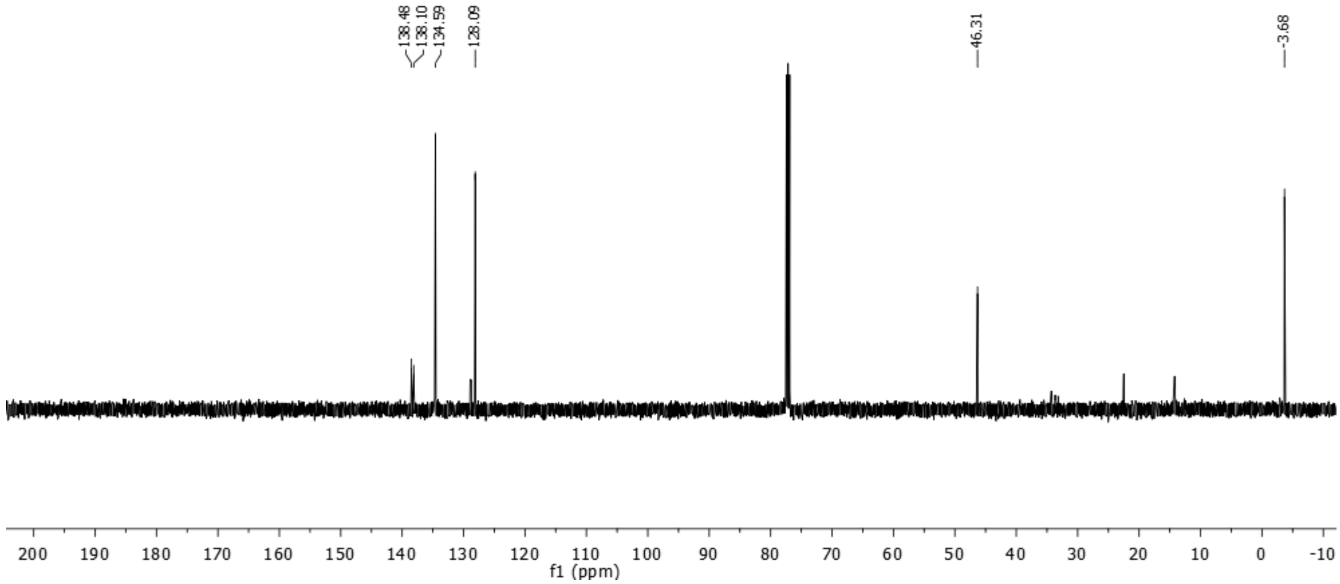


**Figure S67:** $^{13}C\{^{1}H\}$ NMR spectrum of (4-(chloromethyl)phenyl)dimethylsilane.

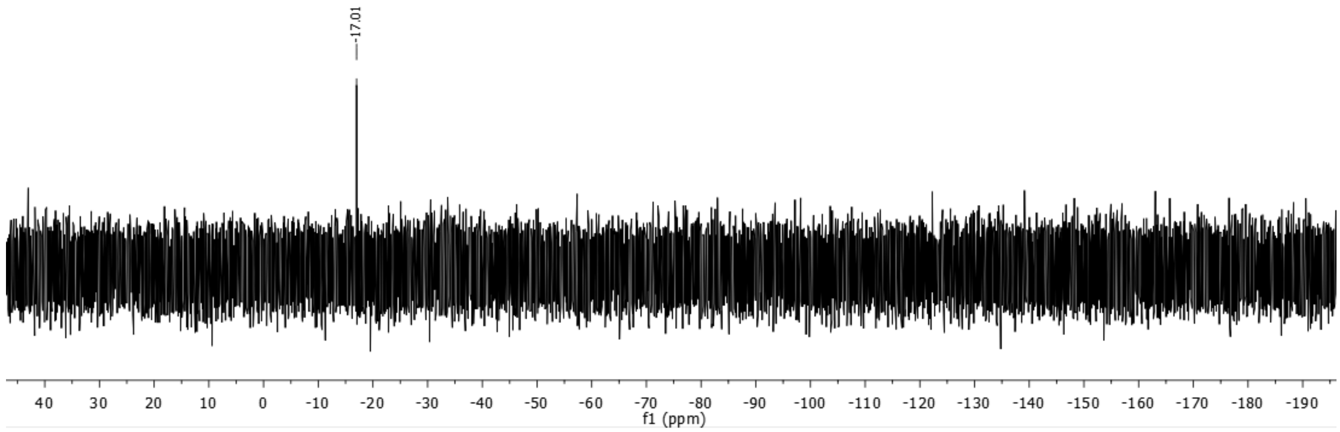


**Figure S68:** $^{29}$Si NMR spectrum of (4-(chloromethyl)phenyl)dimethylsilane.

**4-Bromostyrene**

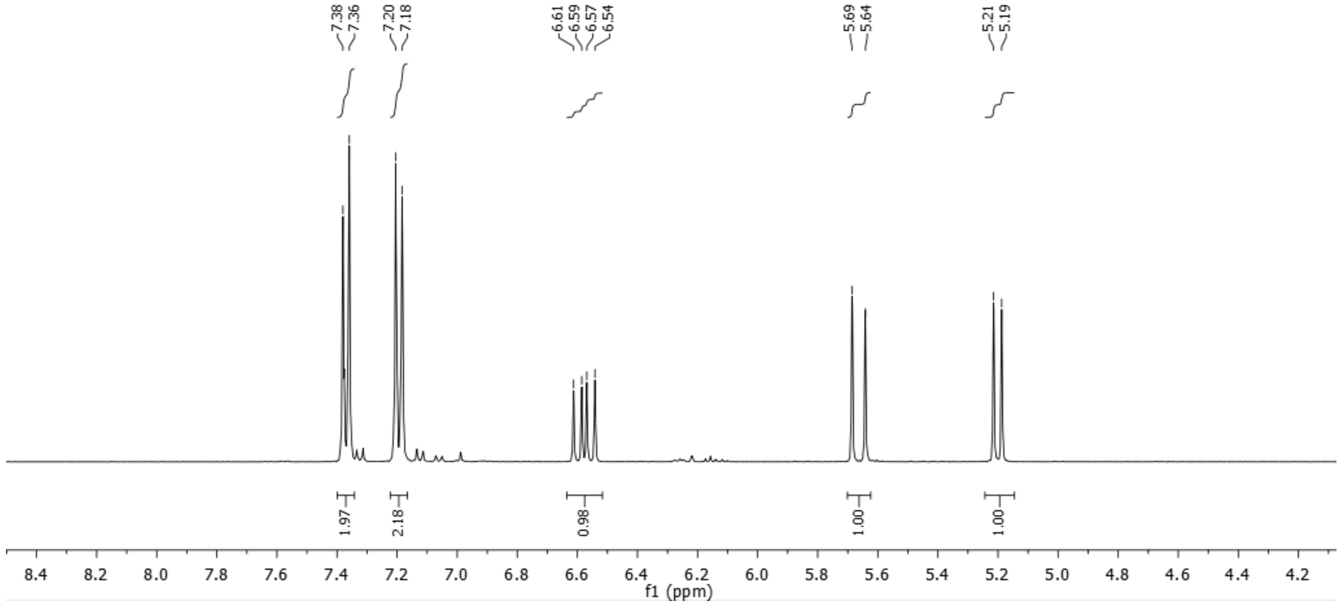


**Figure S69:** $^{1}$H NMR spectrum of 4-bromostyrene.

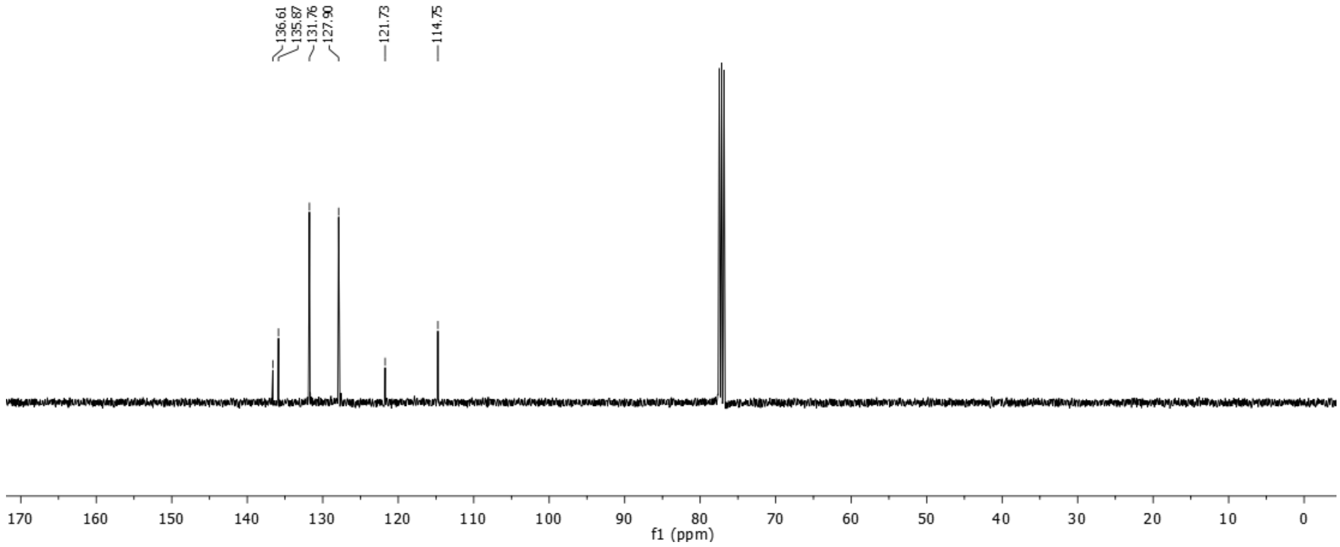


**Figure S70:** $^{13}C\{^1H\}$ NMR spectrum of 4-bromostyrene.

**Dimethyl(4-vinylphenyl)silane**

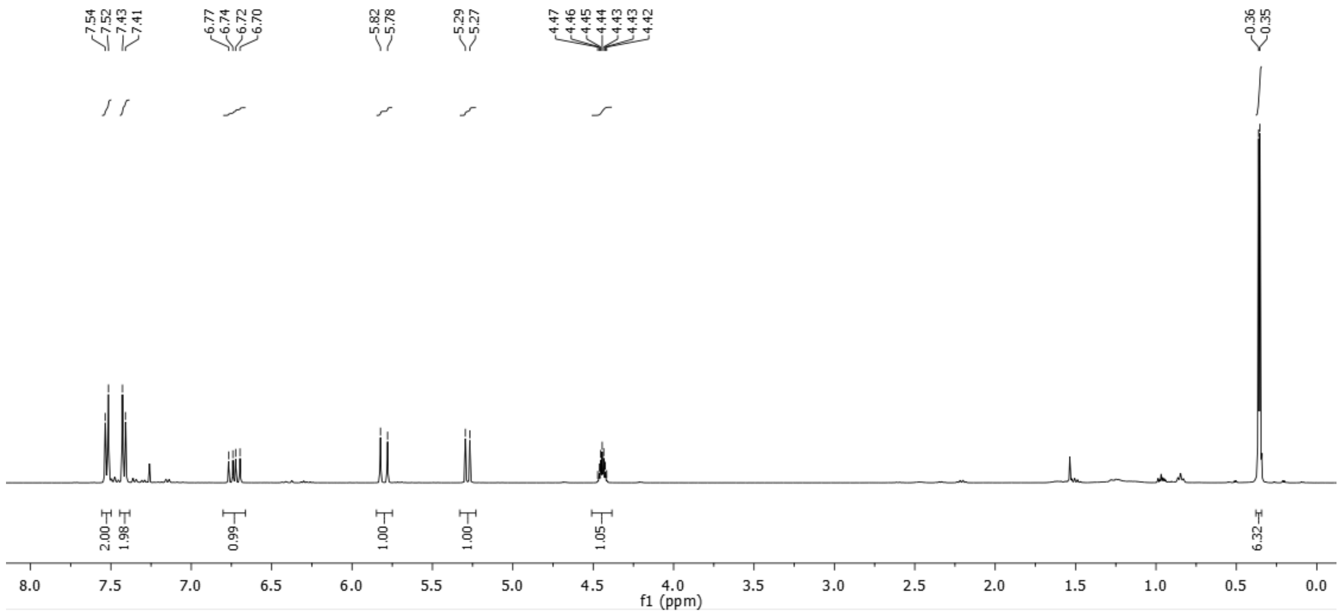


**Figure S71:** $^1H$ NMR spectrum of dimethyl(4-vinylphenyl)silane.

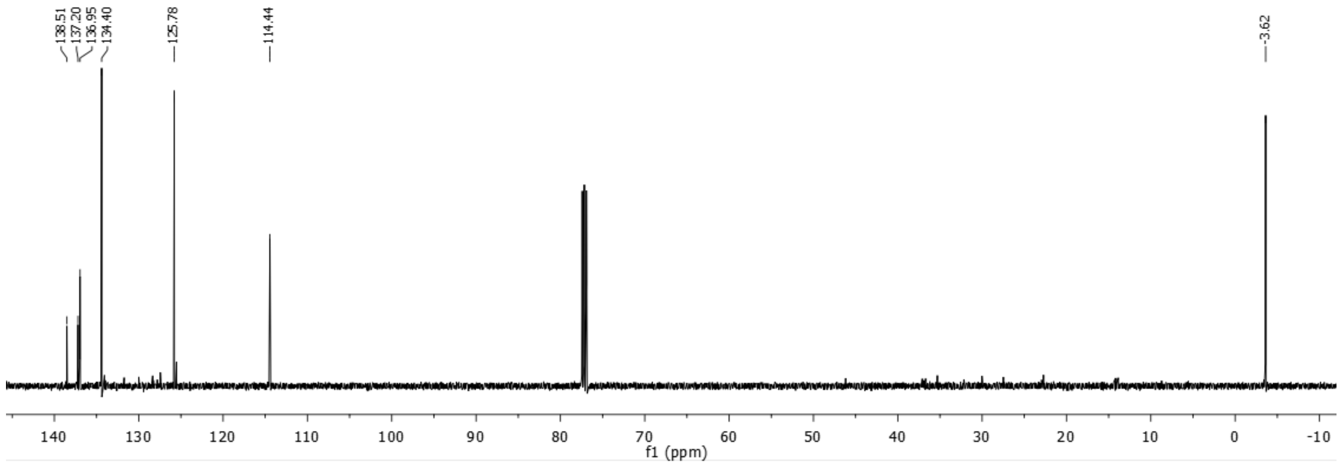


**Figure S72:** $^{13}C\{^1H\}$ NMR spectrum of dimethyl(4-vinylphenyl)silane.

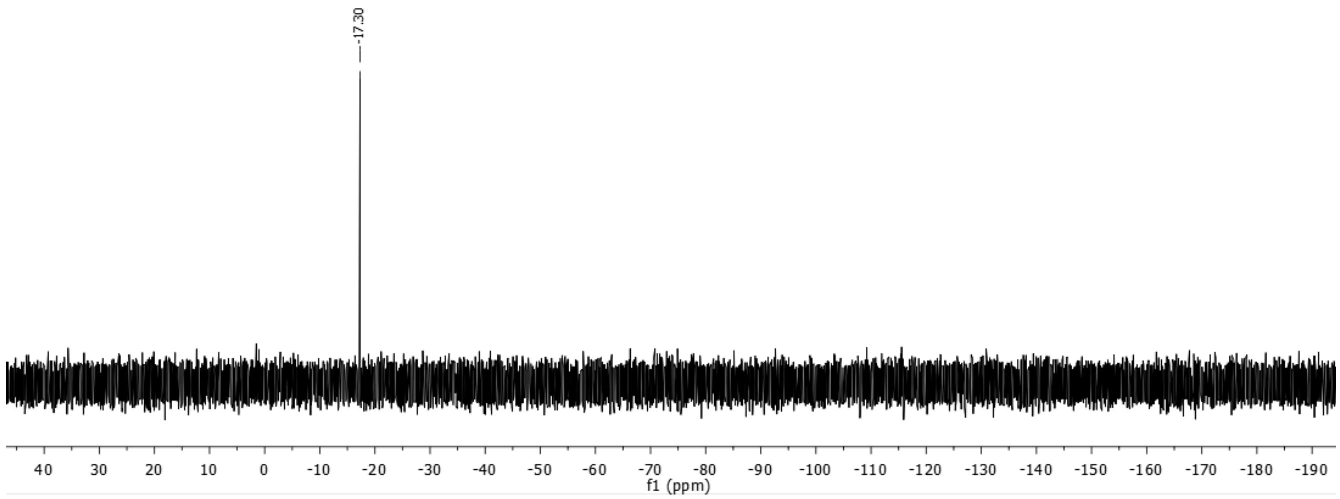


**Figure S73:** $^{29}Si$ NMR spectrum of dimethyl(4-vinylphenyl)silane.

# 13 Computational Detail

## 13.1 Computational Method

Density functional theory calculations were executed using Gaussian 16, A.03.[12] All geometry optimisations were computed with the BP86 functional, accompanied by the ultrafine integral grid option- 'int=grid=ultrafine'. Iron atoms were defined using the Struttgart-Dresden Effective Core Potentials and basis sets (SDDAll).[13] Other atoms were described with double-$\zeta$ plus polarization 6-31G** basis sets, defined as '**BS1**'.[14,15] Frequency calculations at the same level of theory were used to generate free energies, with energy minima (confirmed with no imaginary frequencies), corresponding to the relevant intermediate species along the reaction coordinate and saddle points (confirmed with one imaginary frequency), corresponding to the relevant TS. Single point energy corrections were calculated at the B3PW91-D3BJ/Def2-TZVP/IEF-PCM($C_6H_6$) level, with Ahlrichs triple-$\zeta$ basis set deployed on

all atoms.[16] This method follows that employed by Webster and co-workers, following successful benchmarking against an experimental $\beta$-hydrogen transfer with an analogous iron $\beta$-diketiminate system.[17] Free-energy profiles are valued in kcalmol$^{-1}$ at the B3PW91-D3BJ/Def2-TZVP/IEF-PCM($C_6H_6$)//BP86/**BS1** theory level described above.[18]

Following Webster and co-workers study,[17] empirical dispersion corrections were calculated with Grimme's D3[19] addition to KS-DFT, with the Becke-Johnson damping function.[20] Benzene solvent corrections were deployed using the IEF-PCM implemented in Gaussian 16, using optimised geometries at the BP86/**BS1** level.[21] All corrections and scaling factors were applied using the GoodVibes programme, frequency cut-off 100.0 cm$^{-1}$ (T = 298.15 K, C = 1.0 molL$^{-1}$, vibrational scale factor = 1.0).[22]

All iron containing structures for catalyst activation were optimised in both the quintet (denoted $^{5}$**X**) and triplet (denoted $^{3}$**X**) spin states to identify whether any spin-crossover mechanism is operable, again following preceding work by Webster and co-workers.[17] TSs for the H/D exchange on the triplet energy surface did not converge. Given the triplet state is consistently higher in energy than the quintet, these structures were omitted from the mechanistic investigation.

## 13.2 DFT-Derived Mechanism

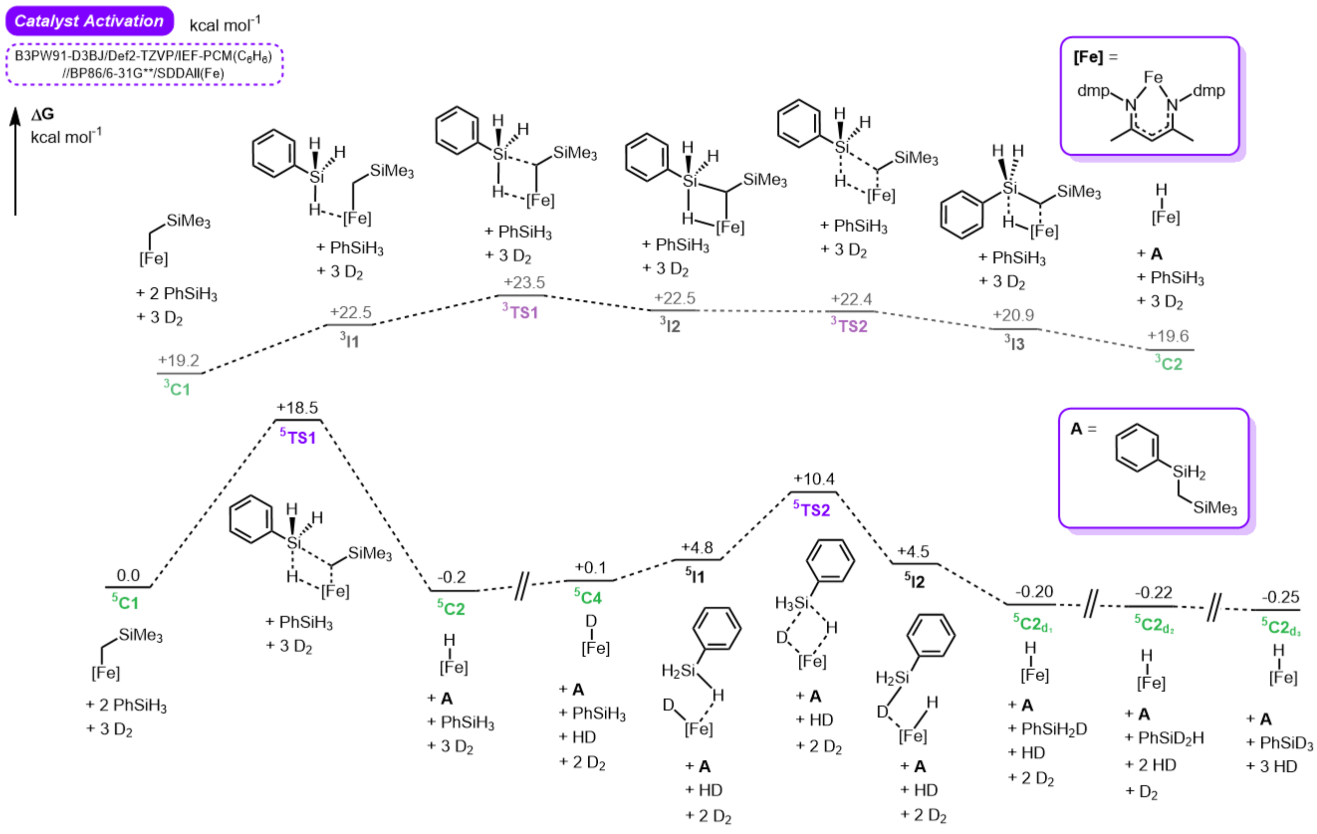


**Figure S74:** DFT-derived energy level diagram for catalyst activation and H/D exchange. Energies are calculated at the B3PW91-D3BJ/Def2-TZVP/IEF-PCM($C_6H_6$)//BP86/**BS1** level of theory. All energies are reported in kcal mol$^{-1}$ and referenced to $^{5}$**C1** and reactants.

## 13.3 Atomic Coordinates

### 13.3.1 Catalyst Activation

**Phenylsilane**

B3PW91-D3BJ/Def2-TZVP/IEF-PCM($C_6H_6$) Energy = -522.854721

Number of imaginary frequencies = 0

Lowest Frequency = 20.70

BP86/**BS1** Geometry

| | | | |
|---|---|---|---|
| C | 0.25681 | 1.21265 | -0.01036 |
| C | 1.66007 | 1.21460 | 0.00344 |
| C | 2.36398 | 0.00000 | 0.01136 |
| C | 1.66007 | -1.21460 | 0.00344 |
| C | 0.25681 | -1.21264 | -0.01037 |
| C | -0.47110 | 0.00001 | -0.01310 |
| H | -0.27752 | 2.16996 | -0.02472 |
| H | 2.20467 | 2.16481 | 0.00349 |
| H | 3.45906 | -0.00001 | 0.01906 |
| H | 2.20466 | -2.16482 | 0.00350 |
| H | -0.27753 | -2.16995 | -0.02472 |
| Si | -2.35776 | 0.00000 | 0.00636 |
| H | -2.87872 | -1.22259 | -0.68754 |
| H | -2.90700 | 0.00034 | 1.40352 |
| H | -2.87878 | 1.22223 | -0.68813 |

**$PhSiH_2CH_2TMS$, A**

B3PW91-D3BJ/Def2-TZVP/IEF-PCM($C_6H_6$) Energy = -970.760535

Number of imaginary frequencies = 0

Lowest Frequency = 10.31

BP86/**BS1** Geometry

| | | | |
|---|---|---|---|
| Si | 2.87087 | -0.00004 | -0.19511 |
| C | 1.00427 | -0.00011 | -0.59551 |
| H | 0.78017 | -0.88464 | -1.22286 |
| H | 0.78014 | 0.88408 | -1.22331 |
| C | 3.84164 | -0.00026 | -1.83130 |
| H | 3.60549 | -0.89076 | -2.43927 |
| H | 4.93048 | -0.00069 | -1.64849 |
| H | 3.60617 | 0.89044 | -2.43924 |
| C | 3.32196 | -1.55433 | 0.80089 |

| | | | |
|---|---|---|---|
| H | 2.80208 | -1.57981 | 1.77359 |
| H | 4.40697 | -1.59335 | 1.00035 |
| H | 3.05130 | -2.47477 | 0.25501 |
| C | 3.32200 | 1.55439 | 0.80065 |
| H | 3.05079 | 2.47479 | 0.25498 |
| H | 4.40712 | 1.59368 | 0.99951 |
| H | 2.80267 | 1.57966 | 1.77366 |
| C | -4.04325 | -1.21468 | -0.32072 |
| C | -2.70851 | -1.21143 | 0.11300 |
| C | -2.01228 | 0.00012 | 0.33440 |
| C | -2.70867 | 1.21149 | 0.11255 |
| C | -4.04341 | 1.21440 | -0.32118 |
| C | -4.71282 | -0.00023 | -0.5399 |
| H | -4.56342 | -2.16499 | -0.48279 |
| H | -2.20346 | -2.16906 | 0.28903 |
| H | -2.20375 | 2.16925 | 0.28822 |
| H | -4.56371 | 2.16457 | -0.48362 |
| H | -5.75534 | -0.00036 | -0.87550 |
| Si | -0.19742 | 0.00036 | 0.86869 |
| H | 0.03618 | 1.21896 | 1.71617 |
| H | 0.03630 | -1.21753 | 1.71714 |

**$D_2$**

B3PW91-D3BJ/Def2-TZVP/IEF-PCM($C_6H_6$) Energy = -1.181925

Number of imaginary frequencies = 0

Lowest Frequency = 3081.95

BP86/**BS1** Geometry

| | | | |
|---|---|---|---|
| H(Iso=2) | 0.00000 | 0.00000 | 0.37523 |
| H(Iso=2) | 0.00000 | 0.00000 | -0.37523 |

**HD**

B3PW91-D3BJ/Def2-TZVP/IEF-PCM($C_6H_6$) Energy = -1.179560

Number of imaginary frequencies = 0

Lowest Frequency = 3773.64

BP86/**BS1** Geometry

| | | | |
|---|---|---|---|
| H(Iso=2) | 0.00000 | 0.00000 | 0.37523 |
| H | 0.00000 | 0.00000 | -0.37523 |

**$^5$C1**

B3PW91-D3BJ/Def2-TZVP/IEF-PCM($C_6H_6$) Energy = -2636.794718

Number of imaginary frequencies = 0

Lowest Frequency = 16.72

BP86/**BS1** Geometry

| | | | |
|---|---|---|---|
| Fe | 0.06287 | 0.04069 | 0.30265 |
| Si | -0.39929 | 3.31717 | 0.00204 |
| N | -1.3251 | -1.3163 | -0.09575 |
| N | 1.58222 | -1.1738 | -0.09414 |
| C | -1.07888 | -2.60881 | -0.41314 |
| C | 0.22474 | -3.13373 | -0.56429 |
| H | 0.27726 | -4.1946 | -0.82132 |
| C | 1.47072 | -2.48063 | -0.41907 |
| C | 2.73145 | -3.3054 | -0.6237 |
| H | 3.35931 | -2.88791 | -1.42966 |
| H | 2.48457 | -4.3466 | -0.87882 |
| H | 3.35553 | -3.30741 | 0.2871 |
| C | -2.24934 | -3.55978 | -0.60774 |
| H | -2.86881 | -3.61869 | 0.30426 |
| H | -1.8972 | -4.57213 | -0.85506 |
| H | -2.91816 | -3.21594 | -1.41568 |
| C | -2.67811 | -0.8579 | 0.03308 |
| C | -3.29321 | -0.84583 | 1.31371 |
| C | -4.60622 | -0.35151 | 1.42513 |
| H | -5.08725 | -0.34385 | 2.41012 |
| C | -5.29803 | 0.12677 | 0.30371 |
| H | -6.31879 | 0.50856 | 0.40868 |
| C | -4.67405 | 0.12152 | -0.95191 |
| H | -5.20756 | 0.50213 | -1.83057 |
| C | -3.36145 | -0.36185 | -1.11016 |
| C | -2.55042 | -1.35202 | 2.53085 |
| H | -1.63412 | -0.76108 | 2.71615 |
| C | -2.68863 | -0.34476 | -2.465 |
| H | -2.40626 | -1.35693 | -2.80531 |
| C | 2.87603 | -0.5679 | 0.03219 |
| C | 3.48524 | -0.48606 | 1.31301 |
| C | 4.72752 | 0.16552 | 1.4269 |

| | | | |
|---|---|---|---|
| H | 5.20383 | 0.22877 | 2.41218 |
| C | 5.35464 | 0.73033 | 0.30751 |
| H | 6.32075 | 1.23443 | 0.41431 |
| C | 4.73662 | 0.65151 | -0.94847 |
| H | 5.22001 | 1.09651 | -1.82598 |
| C | 3.49385 | 0.01073 | -1.1093 |
| C | 2.8079 | -1.07983 | 2.52858 |
| H | 2.60852 | -2.15938 | 2.40814 |
| C | 2.82495 | -0.05026 | -2.46497 |
| H | 1.84236 | 0.45494 | -2.44972 |
| C | 0.13198 | 1.87254 | 1.09539 |
| H | -0.48933 | 1.88246 | 2.01551 |
| H | 1.18733 | 2.01953 | 1.40799 |
| C | -2.29712 | 3.41407 | -0.08686 |
| H | -2.73896 | 2.47187 | -0.4545 |
| H | -2.62672 | 4.22785 | -0.75685 |
| H | -2.72721 | 3.61054 | 0.91103 |
| C | 0.30011 | 3.12025 | -1.76332 |
| H | 1.39864 | 3.0093 | -1.74502 |
| H | 0.06357 | 3.99662 | -2.39269 |
| H | -0.1189 | 2.23101 | -2.26923 |
| C | 0.25442 | 4.97357 | 0.69278 |
| H | -0.12217 | 5.15356 | 1.71487 |
| H | -0.05887 | 5.82657 | 0.0644 |
| H | 1.35758 | 4.97935 | 0.73987 |
| H | 3.42824 | -0.94498 | 3.42917 |
| H | 1.8288 | -0.60016 | 2.7139 |
| H | 3.44665 | 0.44042 | -3.23094 |
| H | 2.63647 | -1.08791 | -2.79275 |
| H | -3.35042 | 0.09775 | -3.22655 |
| H | -1.75344 | 0.24379 | -2.43966 |
| H | -3.18218 | -1.28835 | 3.43133 |
| H | -2.22547 | -2.40096 | 2.41345 |

**$^5$TS1**

B3PW91-D3BJ/Def2-TZVP/IEF-PCM($C_6H_6$) Energy = -3159.619893

Number of imaginary frequencies = 1

Lowest Frequency = 67.39$i$

BP86/**BS1** Geometry

| | | | |
|---|---|---|---|
| Fe | -0.26958 | 0.36505 | -0.0246 |
| Si | -1.07293 | -2.89611 | -2.14115 |
| N | -1.80359 | 1.41121 | 0.70636 |
| N | 0.83822 | 1.95943 | -0.3615 |
| C | -1.7917 | 2.73748 | 0.94374 |
| C | -0.68214 | 3.56498 | 0.64873 |
| H | -0.79827 | 4.62078 | 0.90483 |
| C | 0.52673 | 3.22202 | 0.00441 |
| C | 1.50245 | 4.34357 | -0.31275 |
| H | 2.49168 | 4.15497 | 0.13744 |
| H | 1.12726 | 5.30792 | 0.06015 |
| H | 1.66467 | 4.43076 | -1.40134 |
| C | -3.02829 | 3.39856 | 1.53221 |
| H | -3.89446 | 3.29896 | 0.85488 |
| H | -2.85316 | 4.46921 | 1.71367 |
| H | -3.32067 | 2.92462 | 2.4848 |
| C | -2.99866 | 0.64933 | 0.92423 |
| C | -3.93568 | 0.50873 | -0.13727 |
| C | -5.07324 | -0.29451 | 0.06919 |
| H | -5.79952 | -0.40375 | -0.74464 |
| C | -5.28725 | -0.94511 | 1.2927 |
| H | -6.17542 | -1.56914 | 1.43565 |
| C | -4.35884 | -0.79281 | 2.33164 |
| H | -4.524 | -1.29595 | 3.29127 |
| C | -3.20632 | -0.00056 | 2.17168 |
| C | -3.72877 | 1.22431 | -1.45449 |
| H | -2.73334 | 1.01245 | -1.88493 |
| C | -2.22205 | 0.16758 | 3.30658 |
| H | -2.16341 | 1.21617 | 3.65077 |
| C | 2.03788 | 1.6888 | -1.10197 |
| C | 1.97844 | 1.66699 | -2.52385 |
| C | 3.13939 | 1.32536 | -3.24095 |
| H | 3.1003 | 1.30816 | -4.33635 |
| C | 4.33365 | 1.01133 | -2.57585 |
| H | 5.22623 | 0.73943 | -3.14889 |
| C | 4.38001 | 1.04725 | -1.17609 |

| | | | |
|---|---|---|---|
| H | 5.31179 | 0.80599 | -0.65221 |
| C | 3.24528 | 1.38767 | -0.41425 |
| C | 0.69996 | 2.02286 | -3.25192 |
| H | 0.36523 | 3.0497 | -3.01997 |
| C | 3.32831 | 1.4625 | 1.09311 |
| H | 2.42076 | 1.06305 | 1.57152 |
| C | -0.24372 | -1.32843 | -1.39151 |
| H | -1.15967 | -0.65285 | -1.42671 |
| H | 0.48723 | -0.9516 | -2.13303 |
| C | -1.70065 | -2.39886 | -3.87221 |
| H | -2.42213 | -1.56477 | -3.82162 |
| H | -2.20918 | -3.24825 | -4.36248 |
| H | -0.86924 | -2.08556 | -4.52742 |
| C | -2.55522 | -3.40559 | -1.07385 |
| H | -2.234 | -3.75465 | -0.07881 |
| H | -3.11352 | -4.22718 | -1.55649 |
| H | -3.25536 | -2.56559 | -0.92279 |
| C | 0.13357 | -4.34517 | -2.34564 |
| H | 1.02301 | -4.05459 | -2.92948 |
| H | -0.37066 | -5.16862 | -2.88278 |
| H | 0.48241 | -4.73373 | -1.37582 |
| C | 2.99969 | -1.42212 | 3.97645 |
| C | 1.99677 | -1.41174 | 2.99434 |
| C | 2.24192 | -1.87776 | 1.68387 |
| C | 3.54202 | -2.34095 | 1.38593 |
| C | 4.55843 | -2.34512 | 2.35748 |
| C | 4.28782 | -1.88637 | 3.65693 |
| H | 2.7815 | -1.06425 | 4.98936 |
| H | 0.99881 | -1.03082 | 3.25087 |
| H | 3.75839 | -2.70525 | 0.37333 |
| H | 5.56136 | -2.7061 | 2.10126 |
| H | 5.07647 | -1.88798 | 4.41761 |
| Si | 0.85563 | -1.99977 | 0.33304 |
| H | 0.04419 | -0.77814 | 1.21765 |
| H | 1.7883 | -2.58876 | -0.72307 |
| H | -0.07545 | -3.0892 | 0.78275 |
| H | 4.18954 | 0.88862 | 1.46854 |

| | | | |
|---|---|---|---|
| H | 3.44477 | 2.50733 | 1.43925 |
| H | 0.83727 | 1.94882 | -4.34267 |
| H | -0.13319 | 1.35539 | -2.96708 |
| H | -2.50704 | -0.45617 | 4.16875 |
| H | -1.20312 | -0.11508 | 2.98897 |
| H | -4.4937 | 0.92467 | -2.18864 |
| H | -3.78248 | 2.32235 | -1.34084 |

**$^5$C2**

B3PW91-D3BJ/Def2-TZVP/IEF-PCM($C_6H_6$) Energy = -2188.889209

Number of imaginary frequencies = 0

Lowest Frequency = 23.35

BP86/**BS1** Geometry

| | | | |
|---|---|---|---|
| Fe | 0.00000 | -0.00007 | -0.90725 |
| N | 1.44677 | 0.00001 | 0.42509 |
| N | -1.44677 | 0.00004 | 0.42509 |
| C | 1.27549 | 0.00016 | 1.76643 |
| C | 0.00000 | 0.00015 | 2.37683 |
| H | 0.00000 | 0.00021 | 3.46963 |
| C | -1.27549 | 0.00005 | 1.76643 |
| C | -2.49841 | -0.00003 | 2.66853 |
| H | -3.13271 | 0.88359 | 2.48021 |
| H | -2.20641 | 0.00002 | 3.72907 |
| H | -3.13255 | -0.88377 | 2.48025 |
| C | 2.49841 | 0.00035 | 2.66853 |
| H | 3.13269 | -0.88331 | 2.48033 |
| H | 2.20641 | 0.00046 | 3.72907 |
| H | 3.13257 | 0.88405 | 2.48012 |
| C | 2.76412 | 0.00001 | -0.14377 |
| C | 3.3898 | -1.23676 | -0.45603 |
| C | 4.6628 | -1.21248 | -1.05491 |
| H | 5.15295 | -2.16296 | -1.29523 |
| C | 5.30113 | -0.00001 | -1.35084 |
| H | 6.29114 | -0.00001 | -1.81846 |
| C | 4.66263 | 1.21246 | -1.0553 |
| H | 5.15264 | 2.16294 | -1.29593 |
| C | 3.38963 | 1.23675 | -0.45644 |

| | | | |
|---|---|---|---|
| C | 2.69507 | -2.54911 | -0.16673 |
| H | 1.74933 | -2.63273 | -0.7338 |
| C | 2.69471 | 2.5491 | -0.16756 |
| H | 2.42983 | 2.65722 | 0.89900 |
| C | -2.76412 | -0.00002 | -0.14377 |
| C | -3.38963 | -1.23680 | -0.4563 |
| C | -4.66263 | -1.21257 | -1.05517 |
| H | -5.15264 | -2.16308 | -1.29569 |
| C | -5.30113 | -0.00013 | -1.35084 |
| H | -6.29114 | -0.00018 | -1.81846 |
| C | -4.66280 | 1.21236 | -1.05504 |
| H | -5.15294 | 2.16282 | -1.29547 |
| C | -3.38980 | 1.23671 | -0.45617 |
| C | -2.69472 | -2.54912 | -0.16728 |
| H | -2.42982 | -2.65711 | 0.89929 |
| C | -2.69507 | 2.54909 | -0.16701 |
| H | -1.74932 | 2.63264 | -0.73408 |
| H | 0.00000 | -0.00019 | -2.51953 |
| H | -3.33378 | 3.40145 | -0.44865 |
| H | -2.43033 | 2.65708 | 0.89960 |
| H | -3.33337 | -3.40153 | -0.44886 |
| H | -1.74904 | -2.63256 | -0.73448 |
| H | 3.33335 | 3.40149 | -0.44925 |
| H | 1.74902 | 2.63247 | -0.73476 |
| H | 3.33379 | -3.40149 | -0.44826 |
| H | 2.43033 | -2.65697 | 0.89989 |

### $^{3}$C1

B3PW91-D3BJ/Def2-TZVP/IEF-PCM($C_6H_6$) Energy = -2636.76416

Number of imaginary frequencies = 0

Lowest Frequency = 12.68

BP86/**BS1** Geometry

| | | | |
|---|---|---|---|
| Fe | -0.01317 | -0.03651 | 0.2351 |
| Si | -0.21592 | 3.25811 | 0.00372 |
| N | -1.32319 | -1.38143 | -0.12058 |
| N | 1.43103 | -1.18321 | -0.10736 |
| C | -1.14969 | -2.67336 | -0.45723 |

| | | | |
|---|---|---|---|
| C | 0.14742 | -3.19845 | -0.61829 |
| H | 0.22615 | -4.25288 | -0.8926 |
| C | 1.35712 | -2.49586 | -0.45321 |
| C | 2.65347 | -3.26108 | -0.66983 |
| H | 3.26232 | -2.80841 | -1.47118 |
| H | 2.43968 | -4.30514 | -0.94272 |
| H | 3.28056 | -3.25863 | 0.23831 |
| C | -2.35297 | -3.57544 | -0.65183 |
| H | -2.96248 | -3.63008 | 0.26758 |
| H | -2.03905 | -4.59532 | -0.9201 |
| H | -3.01817 | -3.19593 | -1.44719 |
| C | -2.61996 | -0.80166 | 0.04108 |
| C | -3.20578 | -0.74687 | 1.33859 |
| C | -4.46211 | -0.13017 | 1.48131 |
| H | -4.92028 | -0.08404 | 2.47582 |
| C | -5.12807 | 0.42022 | 0.3771 |
| H | -6.10428 | 0.89837 | 0.50824 |
| C | -4.53836 | 0.36583 | -0.8947 |
| H | -5.05509 | 0.80238 | -1.75696 |
| C | -3.28183 | -0.23604 | -1.08717 |
| C | -2.50078 | -1.35564 | 2.53036 |
| H | -1.47486 | -0.95789 | 2.6364 |
| C | -2.64571 | -0.28493 | -2.45781 |
| H | -2.55509 | -1.31936 | -2.83548 |
| C | 2.72867 | -0.5788 | 0.04219 |
| C | 3.3543 | -0.58069 | 1.31592 |
| C | 4.61462 | 0.03192 | 1.44276 |
| H | 5.10505 | 0.03698 | 2.42306 |
| C | 5.24071 | 0.63381 | 0.34178 |
| H | 6.22145 | 1.10669 | 0.45847 |
| C | 4.60396 | 0.63415 | -0.90768 |
| H | 5.08655 | 1.11037 | -1.76902 |
| C | 3.34287 | 0.03414 | -1.08072 |
| C | 2.66531 | -1.19882 | 2.50965 |
| H | 2.45103 | -2.27192 | 2.35915 |
| C | 2.64324 | 0.06584 | -2.41925 |
| H | 1.67226 | 0.58886 | -2.33729 |

| | | | |
|---|---|---|---|
| C | 0.62396 | 1.76071 | 0.79979 |
| H | 0.40605 | 1.77776 | 1.8936 |
| H | 1.71586 | 1.89509 | 0.70021 |
| C | -2.07947 | 3.30583 | 0.39448 |
| H | -2.61489 | 2.43259 | -0.01665 |
| H | -2.54911 | 4.21359 | -0.02471 |
| H | -2.25272 | 3.31801 | 1.48519 |
| C | 0.02671 | 3.2787 | -1.88763 |
| H | 1.09767 | 3.22111 | -2.15023 |
| H | -0.37637 | 4.20464 | -2.33529 |
| H | -0.48101 | 2.42508 | -2.3701 |
| C | 0.55542 | 4.86703 | 0.69279 |
| H | 0.43146 | 4.93058 | 1.78800 |
| H | 0.08487 | 5.76265 | 0.24837 |
| H | 1.63748 | 4.91343 | 0.47838 |
| H | 3.28046 | -1.09609 | 3.41796 |
| H | 1.68914 | -0.70982 | 2.68860 |
| H | 3.25707 | 0.58494 | -3.17277 |
| H | 2.41923 | -0.94707 | -2.79845 |
| H | -3.2377 | 0.29083 | -3.18682 |
| H | -1.62014 | 0.12559 | -2.4345 |
| H | -3.05306 | -1.14972 | 3.46108 |
| H | -2.39800 | -2.45119 | 2.42995 |

**$^{3}$I1**

B3PW91-D3BJ/Def2-TZVP/IEF-PCM($C_6H_6$) Energy = -3159.613619

Number of imaginary frequencies = 0

Lowest Frequency = 15.15

BP86/**BS1** Geometry

| | | | |
|---|---|---|---|
| Fe | 0.15224 | -0.4811 | -0.05213 |
| Si | -0.26417 | -2.06949 | 2.98371 |
| N | -1.1848 | -0.89483 | -1.44348 |
| N | 1.69982 | -0.61639 | -1.23266 |
| C | -0.87366 | -1.35497 | -2.6763 |
| C | 0.45316 | -1.49091 | -3.13513 |
| H | 0.56642 | -1.8896 | -4.14597 |
| C | 1.65145 | -1.12574 | -2.48681 |

| | | | |
|---|---|---|---|
| C | 2.9405 | -1.29148 | -3.27784 |
| H | 3.50343 | -0.34395 | -3.32441 |
| H | 2.72622 | -1.62372 | -4.30432 |
| H | 3.6098 | -2.03125 | -2.80664 |
| C | -1.98424 | -1.72403 | -3.64835 |
| H | -2.64202 | -2.50618 | -3.23349 |
| H | -1.5672 | -2.08712 | -4.59926 |
| H | -2.63039 | -0.85472 | -3.86031 |
| C | -2.58031 | -0.72192 | -1.13388 |
| C | -3.30712 | -1.79289 | -0.5508 |
| C | -4.65702 | -1.58068 | -0.20764 |
| H | -5.22134 | -2.40186 | 0.24939 |
| C | -5.2794 | -0.34723 | -0.44097 |
| H | -6.32821 | -0.19884 | -0.1638 |
| C | -4.55319 | 0.6942 | -1.03706 |
| H | -5.0386 | 1.65658 | -1.23722 |
| C | -3.20292 | 0.5279 | -1.39744 |
| C | -2.65807 | -3.14128 | -0.32468 |
| H | -1.66223 | -3.03736 | 0.13752 |
| C | -2.43305 | 1.64609 | -2.0617 |
| H | -2.05344 | 1.34952 | -3.0561 |
| C | 2.98473 | -0.18343 | -0.73708 |
| C | 3.89036 | -1.08479 | -0.11941 |
| C | 5.09618 | -0.57266 | 0.40317 |
| H | 5.79513 | -1.26494 | 0.88765 |
| C | 5.41965 | 0.7843 | 0.2977 |
| H | 6.36099 | 1.16079 | 0.7114 |
| C | 4.53946 | 1.65453 | -0.36164 |
| H | 4.79761 | 2.71362 | -0.47644 |
| C | 3.32129 | 1.192 | -0.88941 |
| C | 3.64303 | -2.57901 | -0.04809 |
| H | 4.47842 | -3.12947 | -0.51779 |
| C | 2.406 | 2.13249 | -1.63929 |
| H | 1.40114 | 2.17704 | -1.18315 |
| C | 0.80577 | -1.55729 | 1.48219 |
| H | 1.06349 | -2.48011 | 0.92027 |
| H | 1.7655 | -1.14295 | 1.84832 |

| | | | |
|---|---|---|---|
| C | -0.36823 | -3.9768 | 2.97883 |
| H | -0.87428 | -4.35724 | 2.07446 |
| H | -0.93226 | -4.34413 | 3.85462 |
| H | 0.63737 | -4.43127 | 3.01387 |
| C | -2.04223 | -1.39173 | 3.02777 |
| H | -2.056 | -0.3056 | 3.21586 |
| H | -2.60376 | -1.87908 | 3.84519 |
| H | -2.58965 | -1.57346 | 2.0882 |
| C | 0.61694 | -1.54011 | 4.58881 |
| H | 0.05555 | -1.88056 | 5.47707 |
| H | 0.71306 | -0.4429 | 4.65246 |
| H | 1.63201 | -1.97023 | 4.64764 |
| C | -2.22175 | 4.68636 | 0.83725 |
| C | -1.75403 | 3.37699 | 1.02831 |
| C | -0.36821 | 3.09748 | 1.10483 |
| C | 0.53185 | 4.18147 | 0.9768 |
| C | 0.06911 | 5.49344 | 0.78145 |
| C | -1.30966 | 5.74795 | 0.7116 |
| H | -3.29898 | 4.8799 | 0.78644 |
| H | -2.48047 | 2.55967 | 1.11813 |
| H | 1.61097 | 3.99616 | 1.03622 |
| H | 0.7852 | 6.31715 | 0.68542 |
| H | -1.6734 | 6.77011 | 0.56036 |
| Si | 0.28256 | 1.35012 | 1.47759 |
| H | -0.90751 | 0.52589 | 0.58551 |
| H | 1.7818 | 1.49481 | 1.51023 |
| H | -0.16299 | 1.00857 | 2.86767 |
| H | 2.82138 | 3.1527 | -1.65757 |
| H | 2.25469 | 1.80793 | -2.68475 |
| H | 3.57727 | -2.92707 | 0.99703 |
| H | 2.71269 | -2.87062 | -0.55662 |
| H | -3.06697 | 2.53762 | -2.19093 |
| H | -1.55091 | 1.93891 | -1.46557 |
| H | -3.28286 | -3.77431 | 0.32563 |
| H | -2.50574 | -3.68961 | -1.2733 |

**$^3$TS1**

B3PW91-D3BJ/Def2-TZVP/IEF-PCM($C_6H_6$) Energy = -3159.611965

Number of imaginary frequencies = 1

Lowest Frequency = 62.16$i$

BP86/**BS1** Geometry

| | | | |
|---|---|---|---|
| Fe | -0.02942 | -0.47824 | -0.07922 |
| Si | 1.21882 | -0.95623 | 3.40842 |
| N | -1.47156 | -1.20834 | -1.16666 |
| N | 1.33115 | -0.57427 | -1.52566 |
| C | -1.35943 | -1.68954 | -2.42163 |
| C | -0.16059 | -1.61883 | -3.15783 |
| H | -0.1994 | -2.02337 | -4.17167 |
| C | 1.08297 | -1.09174 | -2.75395 |
| C | 2.1945 | -1.11707 | -3.79499 |
| H | 2.59881 | -0.10526 | -3.96685 |
| H | 1.82048 | -1.51042 | -4.75165 |
| H | 3.04442 | -1.7407 | -3.4718 |
| C | -2.55479 | -2.35544 | -3.08455 |
| H | -2.88922 | -3.23031 | -2.50027 |
| H | -2.2991 | -2.69228 | -4.09991 |
| H | -3.42044 | -1.6753 | -3.14798 |
| C | -2.77047 | -1.24246 | -0.5405 |
| C | -3.0934 | -2.29829 | 0.35006 |
| C | -4.35056 | -2.28119 | 0.98506 |
| H | -4.60664 | -3.09475 | 1.67364 |
| C | -5.26797 | -1.24933 | 0.74758 |
| H | -6.23968 | -1.25034 | 1.2522 |
| C | -4.93824 | -0.2187 | -0.14466 |
| H | -5.65572 | 0.58647 | -0.3411 |
| C | -3.69541 | -0.196 | -0.80452 |
| C | -2.11589 | -3.42318 | 0.61683 |
| H | -1.23598 | -3.07076 | 1.18596 |
| C | -3.35029 | 0.9153 | -1.76982 |
| H | -3.2076 | 0.54167 | -2.79998 |
| C | 2.68443 | -0.14276 | -1.2771 |
| C | 3.65988 | -1.07499 | -0.82776 |
| C | 4.95453 | -0.60375 | -0.53227 |
| H | 5.7068 | -1.31682 | -0.175 |
| C | 5.29099 | 0.74641 | -0.69501 |

| | | | |
|---|---|---|---|
| H | 6.30123 | 1.09498 | -0.45651 |
| C | 4.32942 | 1.64565 | -1.17504 |
| H | 4.59123 | 2.69926 | -1.3263 |
| C | 3.02079 | 1.2223 | -1.47611 |
| C | 3.35057 | -2.55369 | -0.71223 |
| H | 3.36964 | -3.05229 | -1.6998 |
| C | 2.01464 | 2.19003 | -2.05494 |
| H | 1.07101 | 2.19717 | -1.48403 |
| C | 1.19736 | -0.84407 | 1.49575 |
| H | 0.82362 | -1.87257 | 1.21765 |
| H | 2.24641 | -0.80066 | 1.15419 |
| C | 2.18462 | -2.56851 | 3.76293 |
| H | 1.68375 | -3.45132 | 3.32819 |
| H | 2.27201 | -2.73738 | 4.85131 |
| H | 3.20695 | -2.52526 | 3.34908 |
| C | -0.51529 | -1.17234 | 4.15903 |
| H | -1.14577 | -0.27783 | 4.03302 |
| H | -0.43864 | -1.38354 | 5.24038 |
| H | -1.04222 | -2.02384 | 3.69354 |
| C | 2.18435 | 0.44612 | 4.25486 |
| H | 3.16965 | 0.5851 | 3.77727 |
| H | 2.36109 | 0.1886 | 5.31445 |
| H | 1.65616 | 1.41158 | 4.22359 |
| C | -2.744 | 4.27599 | 0.36353 |
| C | -2.08904 | 3.07821 | 0.69118 |
| C | -0.67731 | 2.98361 | 0.67555 |
| C | 0.05332 | 4.14036 | 0.31863 |
| C | -0.59525 | 5.34112 | -0.0168 |
| C | -1.99712 | 5.41049 | 0.0032 |
| H | -3.83841 | 4.32643 | 0.39119 |
| H | -2.68843 | 2.20312 | 0.97331 |
| H | 1.14943 | 4.10423 | 0.3145 |
| H | -0.00562 | 6.22358 | -0.28996 |
| H | -2.50632 | 6.34527 | -0.25577 |
| Si | 0.23643 | 1.4146 | 1.29923 |
| H | -1.05769 | 0.35304 | 0.76551 |
| H | 1.67511 | 1.87204 | 1.27952 |

| | | | |
|---|---|---|---|
| H | -0.19224 | 1.3319 | 2.7314 |
| H | 2.4184 | 3.21496 | -2.06864 |
| H | 1.7444 | 1.92295 | -3.09348 |
| H | 4.09596 | -3.06062 | -0.07834 |
| H | 2.3506 | -2.73457 | -0.28795 |
| H | -4.1485 | 1.67394 | -1.79548 |
| H | -2.40991 | 1.41735 | -1.48469 |
| H | -2.59219 | -4.2237 | 1.20555 |
| H | -1.72704 | -3.86639 | -0.31614 |

**$^{3}$I2**

B3PW91-D3BJ/Def2-TZVP/IEF-PCM($C_6H_6$) Energy = -3159.613635

Number of imaginary frequencies = 0

Lowest Frequency = 8.46

BP86/**BS1** Geometry

| | | | |
|---|---|---|---|
| Fe | -0.12979 | -0.38949 | -0.09347 |
| Si | 1.24382 | -0.61268 | 3.45398 |
| N | -1.60797 | -1.1263 | -1.10066 |
| N | 1.2056 | -0.69495 | -1.53284 |
| C | -1.5741 | -1.59609 | -2.36309 |
| C | -0.40113 | -1.5861 | -3.14409 |
| H | -0.50001 | -1.97272 | -4.16082 |
| C | 0.89394 | -1.19128 | -2.75675 |
| C | 1.99247 | -1.36816 | -3.79687 |
| H | 2.53097 | -0.42293 | -3.97747 |
| H | 1.56989 | -1.71734 | -4.75053 |
| H | 2.75005 | -2.09868 | -3.46568 |
| C | -2.82269 | -2.21153 | -2.9746 |
| H | -3.13139 | -3.10913 | -2.41024 |
| H | -2.63796 | -2.50677 | -4.01794 |
| H | -3.68028 | -1.51956 | -2.95169 |
| C | -2.85721 | -1.18207 | -0.38126 |
| C | -3.10456 | -2.26595 | 0.50087 |
| C | -4.30667 | -2.27452 | 1.2347 |
| H | -4.50333 | -3.10824 | 1.9187 |
| C | -5.24467 | -1.24259 | 1.09959 |
| H | -6.17196 | -1.26253 | 1.6815 |

| | | | |
|---|---|---|---|
| C | -4.99364 | -0.1881 | 0.21002 |
| H | -5.72945 | 0.6158 | 0.09189 |
| C | -3.8077 | -0.13876 | -0.54606 |
| C | -2.11112 | -3.39903 | 0.64608 |
| H | -1.15132 | -3.05123 | 1.07013 |
| C | -3.55717 | 0.99022 | -1.51939 |
| H | -3.58567 | 0.64469 | -2.56918 |
| C | 2.60636 | -0.46061 | -1.28563 |
| C | 3.41649 | -1.51633 | -0.7842 |
| C | 4.7695 | -1.25021 | -0.49705 |
| H | 5.39611 | -2.05985 | -0.1049 |
| C | 5.31865 | 0.02169 | -0.70984 |
| H | 6.37143 | 0.21282 | -0.47727 |
| C | 4.51474 | 1.0442 | -1.23079 |
| H | 4.9422 | 2.03628 | -1.41675 |
| C | 3.15617 | 0.82441 | -1.531 |
| C | 2.85176 | -2.90671 | -0.58581 |
| H | 2.55668 | -3.37252 | -1.54379 |
| C | 2.31887 | 1.91842 | -2.15189 |
| H | 1.36384 | 2.05958 | -1.61975 |
| C | 1.13415 | -0.53248 | 1.53125 |
| H | 0.60647 | -1.51904 | 1.34543 |
| H | 2.16584 | -0.6437 | 1.15563 |
| C | 2.19999 | -2.23333 | 3.7794 |
| H | 1.66526 | -3.11349 | 3.38155 |
| H | 2.33692 | -2.38971 | 4.86442 |
| H | 3.20135 | -2.20997 | 3.31606 |
| C | -0.46676 | -0.78439 | 4.26097 |
| H | -1.06876 | 0.1335 | 4.17071 |
| H | -0.35684 | -1.01476 | 5.3355 |
| H | -1.03956 | -1.60903 | 3.80186 |
| C | 2.25576 | 0.7963 | 4.22442 |
| H | 3.22716 | 0.90861 | 3.71343 |
| H | 2.45962 | 0.56431 | 5.28504 |
| H | 1.73756 | 1.76662 | 4.18085 |
| C | -2.35423 | 4.50588 | -0.0257 |
| C | -1.80509 | 3.30042 | 0.43973 |

| | | | |
|---|---|---|---|
| C | -0.40821 | 3.08062 | 0.44317 |
| C | 0.41993 | 4.12264 | -0.03161 |
| C | -0.12191 | 5.32847 | -0.5092 |
| C | -1.5123 | 5.52132 | -0.51038 |
| H | -3.43976 | 4.65619 | -0.00746 |
| H | -2.47516 | 2.52168 | 0.82542 |
| H | 1.50885 | 3.99484 | -0.01167 |
| H | 0.54249 | 6.12019 | -0.87388 |
| H | -1.93835 | 6.46118 | -0.87825 |
| Si | 0.36506 | 1.52004 | 1.27556 |
| H | -1.06817 | 0.54249 | 0.75514 |
| H | 1.80767 | 1.97979 | 1.33082 |
| H | -0.19256 | 1.6188 | 2.66465 |
| H | 2.86235 | 2.87672 | -2.15283 |
| H | 2.0562 | 1.68425 | -3.20052 |
| H | 3.59409 | -3.56602 | -0.10802 |
| H | 1.9446 | -2.89363 | 0.04206 |
| H | -4.31868 | 1.77876 | -1.41005 |
| H | -2.56429 | 1.44463 | -1.36551 |
| H | -2.5073 | -4.18362 | 1.31058 |
| H | -1.86785 | -3.86299 | -0.32585 |

**$^3$TS2**

B3PW91-D3BJ/Def2-TZVP/IEF-PCM($C_6H_6$) Energy = -3159.613692

Number of imaginary frequencies = 1

Lowest Frequency = 23.98$i$

BP86/**BS1** Geometry

| | | | |
|---|---|---|---|
| Fe | -0.23399 | -0.23036 | -0.23736 |
| Si | 0.85394 | -2.04579 | 3.02287 |
| N | -1.79449 | -0.37963 | -1.33549 |
| N | 1.02998 | -0.33507 | -1.74271 |
| C | -1.83326 | -0.45313 | -2.68283 |
| C | -0.67138 | -0.44383 | -3.48016 |
| H | -0.82824 | -0.50221 | -4.5596 |
| C | 0.66756 | -0.41221 | -3.0472 |
| C | 1.74054 | -0.4852 | -4.12409 |
| H | 2.42767 | 0.37591 | -4.06812 |

| | | | |
|---|---|---|---|
| H | 1.28422 | -0.50198 | -5.12491 |
| H | 2.36466 | -1.38842 | -4.01303 |
| C | -3.17143 | -0.60272 | -3.38933 |
| H | -3.67959 | -1.53269 | -3.07943 |
| H | -3.03031 | -0.63324 | -4.47983 |
| H | -3.86071 | 0.22361 | -3.14931 |
| C | -3.04239 | -0.43283 | -0.61329 |
| C | -3.50017 | -1.68035 | -0.11439 |
| C | -4.69498 | -1.70915 | 0.63033 |
| H | -5.05215 | -2.66895 | 1.02137 |
| C | -5.42518 | -0.53797 | 0.8726 |
| H | -6.34907 | -0.57711 | 1.45898 |
| C | -4.97006 | 0.68335 | 0.35563 |
| H | -5.54344 | 1.60076 | 0.53271 |
| C | -3.78319 | 0.7593 | -0.39666 |
| C | -2.73614 | -2.95705 | -0.39006 |
| H | -1.69287 | -2.89056 | -0.03181 |
| C | -3.3177 | 2.07556 | -0.97531 |
| H | -3.44343 | 2.11007 | -2.07351 |
| C | 2.44083 | -0.3866 | -1.45928 |
| C | 3.07337 | -1.64387 | -1.25709 |
| C | 4.44023 | -1.66414 | -0.91686 |
| H | 4.93094 | -2.63102 | -0.75446 |
| C | 5.17337 | -0.47595 | -0.79287 |
| H | 6.2342 | -0.50936 | -0.52363 |
| C | 4.54368 | 0.75421 | -1.0267 |
| H | 5.1167 | 1.68568 | -0.95185 |
| C | 3.17859 | 0.82348 | -1.36685 |
| C | 2.30813 | -2.9378 | -1.43232 |
| H | 1.99692 | -3.09172 | -2.48176 |
| C | 2.52596 | 2.15204 | -1.67393 |
| H | 1.59852 | 2.29561 | -1.09595 |
| C | 0.95536 | -1.0609 | 1.35642 |
| H | 0.27128 | -1.74417 | 0.75714 |
| H | 1.97954 | -1.20112 | 0.97046 |
| C | 1.55593 | -3.76661 | 2.60168 |
| H | 0.97258 | -4.26522 | 1.80817 |

| | | | |
|---|---|---|---|
| H | 1.53346 | -4.42049 | 3.49171 |
| H | 2.60331 | -3.70205 | 2.26036 |
| C | -0.94925 | -2.25108 | 3.57418 |
| H | -1.39856 | -1.29346 | 3.88197 |
| H | -1.00653 | -2.94519 | 4.43125 |
| H | -1.57246 | -2.66814 | 2.76447 |
| C | 1.91525 | -1.30015 | 4.40491 |
| H | 2.95286 | -1.13324 | 4.07043 |
| H | 1.94342 | -2.00061 | 5.25884 |
| H | 1.51973 | -0.3378 | 4.76545 |
| C | -0.96084 | 4.71556 | 1.14773 |
| C | -0.853 | 3.32837 | 1.33493 |
| C | 0.40707 | 2.69987 | 1.45588 |
| C | 1.55925 | 3.51577 | 1.39078 |
| C | 1.45991 | 4.90252 | 1.18661 |
| C | 0.19817 | 5.50574 | 1.06252 |
| H | -1.94954 | 5.18217 | 1.0714 |
| H | -1.76392 | 2.7232 | 1.41099 |
| H | 2.54809 | 3.05806 | 1.51446 |
| H | 2.36795 | 5.51399 | 1.1342 |
| H | 0.117 | 6.58763 | 0.91058 |
| Si | 0.57555 | 0.84488 | 1.94218 |
| H | -0.98861 | 0.55222 | 0.90533 |
| H | 1.99485 | 0.94839 | 2.48254 |
| H | -0.31212 | 0.66648 | 3.13932 |
| H | 3.20694 | 2.98688 | -1.44488 |
| H | 2.24768 | 2.23023 | -2.74131 |
| H | 2.92471 | -3.80155 | -1.13606 |
| H | 1.38088 | -2.95079 | -0.8342 |
| H | -3.89185 | 2.91574 | -0.55236 |
| H | -2.24628 | 2.24637 | -0.77864 |
| H | -3.21757 | -3.81737 | 0.10212 |
| H | -2.67379 | -3.17508 | -1.47153 |

**$^3$I3**

B3PW91-D3BJ/Def2-TZVP/IEF-PCM($C_6H_6$) Energy = -3159.616105

Number of imaginary frequencies = 0

Lowest Frequency = 14.99

BP86/**BS1** Geometry

| | | | |
|---|---|---|---|
| Fe | -0.64135 | -0.20973 | -0.21907 |
| Si | 1.79542 | 0.35185 | 3.22624 |
| N | -2.42466 | -0.66031 | -0.53185 |
| N | 0.1444 | -1.78348 | -1.0531 |
| C | -2.84236 | -1.66306 | -1.34168 |
| C | -1.95897 | -2.57601 | -1.95661 |
| H | -2.42164 | -3.32465 | -2.60448 |
| C | -0.55871 | -2.65396 | -1.81905 |
| C | 0.17147 | -3.7503 | -2.57742 |
| H | 0.93768 | -3.32312 | -3.24809 |
| H | -0.53161 | -4.33943 | -3.18511 |
| H | 0.70234 | -4.43901 | -1.89772 |
| C | -4.33254 | -1.83995 | -1.58463 |
| H | -4.87488 | -2.00086 | -0.63653 |
| H | -4.51599 | -2.70474 | -2.23945 |
| H | -4.78094 | -0.94703 | -2.05281 |
| C | -3.39754 | 0.21394 | 0.07137 |
| C | -3.8682 | -0.09181 | 1.37666 |
| C | -4.79869 | 0.77652 | 1.97724 |
| H | -5.1652 | 0.54587 | 2.98435 |
| C | -5.25621 | 1.92036 | 1.30844 |
| H | -5.97816 | 2.58763 | 1.79076 |
| C | -4.78552 | 2.20605 | 0.01923 |
| H | -5.14138 | 3.09881 | -0.50809 |
| C | -3.85763 | 1.36511 | -0.62315 |
| C | -3.38557 | -1.32718 | 2.10383 |
| H | -2.28187 | -1.35226 | 2.15974 |
| C | -3.34907 | 1.69118 | -2.00616 |
| H | -3.57786 | 0.89352 | -2.73553 |
| C | 1.54637 | -2.02028 | -0.85323 |
| C | 1.95958 | -2.92365 | 0.16558 |
| C | 3.33747 | -3.08894 | 0.40238 |
| H | 3.66168 | -3.78538 | 1.18476 |
| C | 4.29002 | -2.38456 | -0.34939 |
| H | 5.35825 | -2.52463 | -0.1533 |
| C | 3.86792 | -1.50813 | -1.35933 |

| | | | |
|---|---|---|---|
| H | 4.60849 | -0.96518 | -1.95775 |
| C | 2.50019 | -1.31063 | -1.63235 |
| C | 0.93468 | -3.68892 | 0.97295 |
| H | 0.35658 | -4.39257 | 0.34734 |
| C | 2.05824 | -0.38062 | -2.74054 |
| H | 1.41012 | 0.42815 | -2.35992 |
| C | 0.86559 | 0.65143 | 1.56203 |
| H | -0.1843 | 0.29962 | 1.75131 |
| H | 1.36361 | -0.01183 | 0.81598 |
| C | 1.62219 | -1.45807 | 3.77108 |
| H | 0.56538 | -1.76547 | 3.85265 |
| H | 2.08786 | -1.60437 | 4.76197 |
| H | 2.12195 | -2.13598 | 3.06002 |
| C | 1.01855 | 1.45897 | 4.55973 |
| H | 1.11403 | 2.53004 | 4.31667 |
| H | 1.51271 | 1.29011 | 5.53273 |
| H | -0.05477 | 1.23531 | 4.68965 |
| C | 3.63518 | 0.75251 | 2.99154 |
| H | 4.07374 | 0.12665 | 2.19504 |
| H | 4.1972 | 0.55503 | 3.92127 |
| H | 3.79209 | 1.81112 | 2.72516 |
| C | 1.40809 | 3.70951 | -2.96731 |
| C | 0.81972 | 3.33132 | -1.7497 |
| C | 1.61001 | 2.92463 | -0.64915 |
| C | 3.0157 | 2.91855 | -0.81299 |
| C | 3.60924 | 3.28425 | -2.0321 |
| C | 2.8049 | 3.68179 | -3.11264 |
| H | 0.77538 | 4.02644 | -3.80372 |
| H | -0.27096 | 3.34914 | -1.65024 |
| H | 3.66133 | 2.63144 | 0.02602 |
| H | 4.69995 | 3.2692 | -2.13511 |
| H | 3.26531 | 3.97553 | -4.06228 |
| Si | 0.85662 | 2.49785 | 1.03607 |
| H | -0.83527 | 1.30294 | -0.47862 |
| H | 1.75731 | 3.21569 | 2.01474 |
| H | -0.50177 | 3.09936 | 1.20012 |
| H | 2.92711 | 0.08394 | -3.23231 |

| | | | |
|---|---|---|---|
| H | 1.47142 | -0.91505 | -3.50931 |
| H | 1.41704 | -4.27026 | 1.77505 |
| H | 0.19487 | -3.00824 | 1.43001 |
| H | -3.79048 | 2.62994 | -2.37767 |
| H | -2.24721 | 1.78829 | -1.99173 |
| H | -3.78973 | -1.3638 | 3.12833 |
| H | -3.68564 | -2.2564 | 1.58644 |

**$^3$C2**

B3PW91-D3BJ/Def2-TZVP/IEF-PCM($C_6H_6$) Energy = -2188.857636

Number of imaginary frequencies = 0

Lowest Frequency = 26.38

BP86/**BS1** Geometry

| | | | |
|---|---|---|---|
| Fe | -0.06487 | 0.25380 | -0.72375 |
| N | 1.34329 | -0.24946 | 0.35586 |
| N | -1.40381 | -0.30283 | 0.47887 |
| C | 1.24375 | -0.91573 | 1.53674 |
| C | 0.01577 | -1.23473 | 2.15405 |
| H | 0.07066 | -1.75963 | 3.11068 |
| C | -1.2695 | -0.92817 | 1.66382 |
| C | -2.49987 | -1.29705 | 2.46790 |
| H | -3.10079 | -0.40302 | 2.71175 |
| H | -2.22107 | -1.79372 | 3.40931 |
| H | -3.16102 | -1.97522 | 1.89973 |
| C | 2.52661 | -1.32407 | 2.24188 |
| H | 3.14325 | -1.98300 | 1.60669 |
| H | 2.29811 | -1.85562 | 3.17758 |
| H | 3.1516 | -0.44646 | 2.48182 |
| C | 2.63615 | 0.09706 | -0.17045 |
| C | 3.2981 | -0.79747 | -1.05261 |
| C | 4.56057 | -0.42838 | -1.55073 |
| H | 5.07960 | -1.11072 | -2.2336 |
| C | 5.15276 | 0.79176 | -1.19459 |
| H | 6.13648 | 1.06137 | -1.59307 |
| C | 4.47933 | 1.66920 | -0.33295 |
| H | 4.93474 | 2.62820 | -0.06018 |
| C | 3.21521 | 1.34259 | 0.19052 |

| | | | |
|---|---|---|---|
| C | 2.63828 | -2.08499 | -1.47955 |
| H | 1.7052 | -1.85707 | -2.0303 |
| C | 2.47919 | 2.30347 | 1.09564 |
| H | 2.23996 | 1.85606 | 2.07662 |
| C | -2.65426 | 0.07348 | -0.09898 |
| C | -3.31718 | -0.82318 | -0.98756 |
| C | -4.50673 | -0.3951 | -1.60433 |
| H | -5.02424 | -1.07801 | -2.28751 |
| C | -5.02884 | 0.88409 | -1.36477 |
| H | -5.95351 | 1.19965 | -1.85904 |
| C | -4.36139 | 1.76245 | -0.49882 |
| H | -4.76581 | 2.76446 | -0.3157 |
| C | -3.17061 | 1.38055 | 0.14407 |
| C | -2.75699 | -2.20224 | -1.25600 |
| H | -2.74248 | -2.82594 | -0.34422 |
| C | -2.45391 | 2.32966 | 1.07862 |
| H | -1.40985 | 2.50317 | 0.75795 |
| H | 0.56349 | 0.33356 | -2.18628 |
| H | -2.96717 | 3.30346 | 1.11933 |
| H | -2.39532 | 1.92944 | 2.10640 |
| H | -3.35713 | -2.72703 | -2.01613 |
| H | -1.71346 | -2.14386 | -1.61328 |
| H | 3.07397 | 3.21438 | 1.26988 |
| H | 1.51256 | 2.60237 | 0.64759 |
| H | 3.30257 | -2.67275 | -2.13318 |
| H | 2.35119 | -2.71593 | -0.61998 |

### 13.3.2 H/D Exchange

**Phenyl(silane-d$_1$)**

B3PW91-D3BJ/Def2-TZVP/IEF-PCM($C_6H_6$) Energy = -522.857101

Number of imaginary frequencies = 0

Lowest Frequency = 18.16

BP86/**BS1** Geometry

| | | | |
|---|---|---|---|
| C | 0.25681 | 1.21265 | -0.01036 |
| C | 1.66007 | 1.21460 | 0.00344 |
| C | 2.36398 | 0.00000 | 0.01136 |
| C | 1.66007 | -1.21460 | 0.00344 |

| | | | |
|---|---|---|---|
| C | 0.25681 | -1.21264 | -0.01037 |
| C | -0.47110 | 0.00001 | -0.01310 |
| H | -0.27752 | 2.16996 | -0.02472 |
| H | 2.20467 | 2.16481 | 0.00349 |
| H | 3.45906 | -0.00001 | 0.01906 |
| H | 2.20466 | -2.16482 | 0.00350 |
| H | -0.27753 | -2.16995 | -0.02472 |
| Si | -2.35776 | 0.00000 | 0.00636 |
| H | -2.87872 | -1.22259 | -0.68754 |
| H | -2.90700 | 0.00034 | 1.40352 |
| H(Iso=2) | -2.87878 | 1.22223 | -0.68813 |

**Phenyl(silane-$d_2$)**

B3PW91-D3BJ/Def2-TZVP/IEF-PCM($C_6H_6$) Energy = -522.859492

Number of imaginary frequencies = 0

Lowest Frequency = 16.46

BP86/**BS1** Geometry

| | | | |
|---|---|---|---|
| C | 0.25681 | 1.21265 | -0.01036 |
| C | 1.66007 | 1.2146 | 0.00344 |
| C | 2.36398 | 0.00000 | 0.01136 |
| C | 1.66007 | -1.2146 | 0.00344 |
| C | 0.25681 | -1.21264 | -0.01037 |
| C | -0.4711 | 0.00001 | -0.0131 |
| H | -0.27752 | 2.16996 | -0.02472 |
| H | 2.20467 | 2.16481 | 0.00349 |
| H | 3.45906 | -0.00001 | 0.01906 |
| H | 2.20466 | -2.16482 | 0.0035 |
| H | -0.27753 | -2.16995 | -0.02472 |
| Si | -2.35776 | 0.00000 | 0.00636 |
| H | -2.87872 | -1.22259 | -0.68754 |
| H(Iso=2) | -2.907 | 0.00034 | 1.40352 |
| H(Iso=2) | -2.87878 | 1.22223 | -0.68813 |

**Phenyl(silane-$d_3$)**

B3PW91-D3BJ/Def2-TZVP/IEF-PCM($C_6H_6$) Energy = -522.861904

Number of imaginary frequencies = 0

Lowest Frequency = 15.12

BP86/**BS1** Geometry

| | | | |
|---|---|---|---|
| C | 0.25681 | 1.21265 | -0.01036 |
| C | 1.66007 | 1.2146 | 0.00344 |
| C | 2.36398 | 0.00000 | 0.01136 |
| C | 1.66007 | -1.2146 | 0.00344 |
| C | 0.25681 | -1.21264 | -0.01037 |
| C | -0.4711 | 0.00001 | -0.0131 |
| H | -0.27752 | 2.16996 | -0.02472 |
| H | 2.20467 | 2.16481 | 0.00349 |
| H | 3.45906 | -0.00001 | 0.01906 |
| H | 2.20466 | -2.16482 | 0.0035 |
| H | -0.27753 | -2.16995 | -0.02472 |
| Si | -2.35776 | 0.00000 | 0.00636 |
| H(Iso=2) | -2.87872 | -1.22259 | -0.68754 |
| H(Iso=2) | -2.907 | 0.00034 | 1.40352 |
| H(Iso=2) | -2.87878 | 1.22223 | -0.68813 |

**$^5$C4**

B3PW91-D3BJ/Def2-TZVP/IEF-PCM($C_6H_6$) Energy = -2188.891064

Number of imaginary frequencies = 0

Lowest Frequency = 23.23

BP86/**BS1** Geometry

| | | | |
|---|---|---|---|
| Fe | 0.00000 | -0.00007 | -0.90725 |
| N | 1.44677 | 0.00001 | 0.42509 |
| N | -1.44677 | 0.00004 | 0.42509 |
| C | 1.27549 | 0.00016 | 1.76643 |
| C | 0.00000 | 0.00015 | 2.37683 |
| H | 0.00000 | 0.00021 | 3.46963 |
| C | -1.27549 | 0.00005 | 1.76643 |
| C | -2.49841 | -0.00003 | 2.66853 |
| H | -3.13271 | 0.88359 | 2.48021 |
| H | -2.20641 | 0.00002 | 3.72907 |
| H | -3.13255 | -0.88377 | 2.48025 |
| C | 2.49841 | 0.00035 | 2.66853 |
| H | 3.13269 | -0.88331 | 2.48033 |
| H | 2.20641 | 0.00046 | 3.72907 |
| H | 3.13257 | 0.88405 | 2.48012 |

| | | | |
|---|---|---|---|
| C | 2.76412 | 0.00001 | -0.14377 |
| C | 3.38980 | -1.23676 | -0.45603 |
| C | 4.66280 | -1.21248 | -1.05491 |
| H | 5.15295 | -2.16296 | -1.29523 |
| C | 5.30113 | -0.00001 | -1.35084 |
| H | 6.29114 | -0.00001 | -1.81846 |
| C | 4.66263 | 1.21246 | -1.0553 |
| H | 5.15264 | 2.16294 | -1.29593 |
| C | 3.38963 | 1.23675 | -0.45644 |
| C | 2.69507 | -2.54911 | -0.16673 |
| H | 1.74933 | -2.63273 | -0.73380 |
| C | 2.69471 | 2.54910 | -0.16756 |
| H | 2.42983 | 2.65722 | 0.89900 |
| C | -2.76412 | -0.00002 | -0.14377 |
| C | -3.38963 | -1.2368 | -0.45630 |
| C | -4.66263 | -1.21257 | -1.05517 |
| H | -5.15264 | -2.16308 | -1.29569 |
| C | -5.30113 | -0.00013 | -1.35084 |
| H | -6.29114 | -0.00018 | -1.81846 |
| C | -4.66280 | 1.21236 | -1.05504 |
| H | -5.15294 | 2.16282 | -1.29547 |
| C | -3.3898 | 1.23671 | -0.45617 |
| C | -2.69472 | -2.54912 | -0.16728 |
| H | -2.42982 | -2.65711 | 0.89929 |
| C | -2.69507 | 2.54909 | -0.16701 |
| H | -1.74932 | 2.63264 | -0.73408 |
| H(Iso=2) | 0.00000 | -0.00019 | -2.51953 |
| H | -3.33378 | 3.40145 | -0.44865 |
| H | -2.43033 | 2.65708 | 0.89960 |
| H | -3.33337 | -3.40153 | -0.44886 |
| H | -1.74904 | -2.63256 | -0.73448 |
| H | 3.33335 | 3.40149 | -0.44925 |
| H | 1.74902 | 2.63247 | -0.73476 |
| H | 3.33379 | -3.40149 | -0.44826 |
| H | 2.43033 | -2.65697 | 0.89989 |

**$^{5}$I1**

B3PW91-D3BJ/Def2-TZVP/IEF-PCM($C_6H_6$) Energy = -2711.738298

Number of imaginary frequencies = 0

Lowest Frequency = 12.44

BP86/**BS1** Geometry

| | | | |
|---|---|---|---|
| Fe | -0.39440 | -0.18540 | 0.44204 |
| N | 0.23384 | -1.96668 | -0.15257 |
| N | -2.27474 | -0.50632 | -0.08479 |
| C | -0.56487 | -2.97176 | -0.57085 |
| C | -1.96142 | -2.82517 | -0.74278 |
| H | -2.49116 | -3.72011 | -1.07841 |
| C | -2.77012 | -1.68849 | -0.51301 |
| C | -4.26876 | -1.83094 | -0.72630 |
| H | -4.81830 | -1.63835 | 0.21194 |
| H | -4.52218 | -2.84248 | -1.07641 |
| H | -4.64609 | -1.10154 | -1.46308 |
| C | 0.04216 | -4.33952 | -0.8401 |
| H | 0.83423 | -4.2886 | -1.60645 |
| H | -0.72520 | -5.05118 | -1.17884 |
| H | 0.51620 | -4.74471 | 0.07117 |
| C | 1.64399 | -2.17601 | 0.00711 |
| C | 2.51217 | -2.00109 | -1.10507 |
| C | 3.89639 | -2.16124 | -0.90729 |
| H | 4.57122 | -2.02728 | -1.76069 |
| C | 4.41704 | -2.48081 | 0.35418 |
| H | 5.49718 | -2.59904 | 0.48924 |
| C | 3.54938 | -2.64147 | 1.44340 |
| H | 3.95146 | -2.88704 | 2.43315 |
| C | 2.15857 | -2.4894 | 1.29444 |
| C | 1.96568 | -1.65202 | -2.47203 |
| H | 1.30117 | -0.77192 | -2.42704 |
| C | 1.23110 | -2.64828 | 2.47919 |
| H | 0.44702 | -3.40358 | 2.29454 |
| C | -3.14526 | 0.61449 | 0.12342 |
| C | -3.4608 | 1.47100 | -0.96666 |
| C | -4.2716 | 2.59569 | -0.72475 |
| H | -4.51818 | 3.25987 | -1.5613 |
| C | -4.75779 | 2.87608 | 0.5597 |
| H | -5.38561 | 3.75689 | 0.72983 |

| | | | |
|---|---|---|---|
| C | -4.43101 | 2.02752 | 1.62666 |
| H | -4.80467 | 2.24480 | 2.63396 |
| C | -3.62219 | 0.89246 | 1.43311 |
| C | -2.93891 | 1.18404 | -2.35766 |
| H | -3.38917 | 0.27335 | -2.79351 |
| C | -3.26114 | -0.00796 | 2.5939 |
| H | -2.17026 | 0.00494 | 2.77668 |
| H | 0.42237 | 0.87935 | -1.02243 |
| H | -3.76523 | 0.32073 | 3.51702 |
| H | -3.53977 | -1.05973 | 2.40537 |
| H | -3.15759 | 2.02223 | -3.03846 |
| H | -1.84813 | 1.01332 | -2.35438 |
| H | 1.79203 | -2.94865 | 3.3789 |
| H | 0.71063 | -1.69799 | 2.7031 |
| H | 2.78466 | -1.43371 | -3.17603 |
| H | 1.36305 | -2.47219 | -2.90334 |
| C | 2.64459 | 2.69712 | 1.07271 |
| C | 3.90464 | 3.08342 | 1.55343 |
| C | 4.85119 | 3.64900 | 0.68250 |
| C | 4.53501 | 3.82691 | -0.67342 |
| C | 3.27451 | 3.43921 | -1.1555 |
| C | 2.30944 | 2.86820 | -0.29206 |
| H | 1.91399 | 2.24540 | 1.75293 |
| H | 4.14888 | 2.94189 | 2.61179 |
| H | 5.83400 | 3.95071 | 1.06072 |
| H | 5.26879 | 4.26780 | -1.35681 |
| H | 3.03916 | 3.58574 | -2.21638 |
| Si | 0.62758 | 2.39734 | -0.98787 |
| H | 0.59593 | 2.80493 | -2.43489 |
| H(Iso=2) | 0.18383 | 0.80562 | 1.59813 |
| H | -0.53153 | 3.01333 | -0.27898 |

**$^5$TS2**

B3PW91-D3BJ/Def2-TZVP/IEF-PCM($C_6H_6$) Energy = -2711.729387

Number of imaginary frequencies = 0

Lowest Frequency = $132.83i$

BP86/**BS1** Geometry

| | | | |
|---|---|---|---|
| Fe | -0.44721 | -0.02619 | 0.00239 |
| N | 0.49029 | 1.73111 | 0.00350 |
| N | -2.28470 | 0.66431 | 0.00249 |
| C | -0.18723 | 2.89509 | 0.00744 |
| C | -1.60180 | 2.99929 | 0.00923 |
| H | -1.99272 | 4.01901 | 0.01264 |
| C | -2.57951 | 1.97895 | 0.00640 |
| C | -4.03982 | 2.39595 | 0.00752 |
| H | -4.56289 | 1.99595 | -0.87812 |
| H | -4.14082 | 3.4908 | 0.01184 |
| H | -4.5637 | 1.98881 | 0.88941 |
| C | 0.60040 | 4.19529 | 0.00986 |
| H | 1.26117 | 4.25218 | 0.89169 |
| H | -0.0703 | 5.06649 | 0.01423 |
| H | 1.25698 | 4.25797 | -0.87472 |
| C | 1.92510 | 1.72575 | 0.00061 |
| C | 2.62285 | 1.69000 | 1.23743 |
| C | 4.02914 | 1.65575 | 1.20896 |
| H | 4.57679 | 1.62984 | 2.15801 |
| C | 4.72917 | 1.64662 | -0.00538 |
| H | 5.82367 | 1.61617 | -0.00770 |
| C | 4.02417 | 1.66291 | -1.21676 |
| H | 4.56792 | 1.64266 | -2.16819 |
| C | 2.61778 | 1.69726 | -1.23925 |
| C | 1.87085 | 1.67955 | 2.54926 |
| H | 1.16346 | 0.83117 | 2.59078 |
| C | 1.86038 | 1.69426 | -2.54801 |
| H | 1.25708 | 2.61028 | -2.68397 |
| C | -3.30418 | -0.34306 | -0.00105 |
| C | -3.77099 | -0.86515 | 1.23677 |
| C | -4.73408 | -1.8903 | 1.20716 |
| H | -5.10181 | -2.29838 | 2.15546 |
| C | -5.21641 | -2.39629 | -0.00824 |
| H | -5.96239 | -3.19775 | -0.01105 |
| C | -4.73289 | -1.88291 | -1.22007 |
| H | -5.09968 | -2.28521 | -2.1712 |
| C | -3.76978 | -0.85760 | -1.24246 |
| C | -3.23583 | -0.33804 | 2.54948 |

| | | | |
|---|---|---|---|
| H | -3.45447 | 0.73645 | 2.68669 |
| C | -3.23328 | -0.32251 | -2.5514 |
| H | -2.13428 | -0.42679 | -2.60154 |
| H | 0.00322 | -1.00257 | 1.24785 |
| H | -3.67319 | -0.86311 | -3.40447 |
| H | -3.45128 | 0.75293 | -2.68201 |
| H | -3.6762 | -0.8842 | 3.39877 |
| H | -2.13681 | -0.44211 | 2.59985 |
| H | 2.55329 | 1.61695 | -3.40112 |
| H | 1.15317 | 0.84585 | -2.59159 |
| H | 2.56725 | 1.59692 | 3.39902 |
| H | 1.26852 | 2.59502 | 2.69308 |
| C | 2.73036 | -2.76309 | -1.21413 |
| C | 4.11188 | -3.01257 | -1.21595 |
| C | 4.80209 | -3.15010 | -0.00057 |
| C | 4.10614 | -3.03636 | 1.21398 |
| C | 2.7246 | -2.78695 | 1.21050 |
| C | 2.01115 | -2.64778 | -0.00227 |
| H | 2.20087 | -2.66505 | -2.16942 |
| H | 4.64981 | -3.10317 | -2.16619 |
| H | 5.87969 | -3.34755 | 0.00004 |
| H | 4.63956 | -3.14560 | 2.16481 |
| H | 2.19050 | -2.70787 | 2.16499 |
| Si | 0.13415 | -2.40047 | -0.00355 |
| H | -0.42460 | -3.20289 | 1.15638 |
| H(Iso=2) | 0.00480 | -0.99873 | -1.24616 |
| H | -0.42370 | -3.19863 | -1.16696 |

**$^{5}$I2**

B3PW91-D3BJ/Def2-TZVP/IEF-PCM($C_6H_6$) Energy = -2711.738759

Number of imaginary frequencies = 0

Lowest Frequency = 12.43

BP86/**BS1** Geometry

| | | | |
|---|---|---|---|
| Fe | 0.39441 | -0.18531 | 0.44207 |
| N | -0.23385 | -1.96669 | -0.15224 |
| N | 2.27474 | -0.50634 | -0.08473 |
| C | 0.56486 | -2.97185 | -0.57037 |

| | | | |
|---|---|---|---|
| C | 1.96141 | -2.82529 | -0.74235 |
| H | 2.49113 | -3.72029 | -1.07784 |
| C | 2.77011 | -1.68858 | -0.51277 |
| C | 4.26875 | -1.83107 | -0.72606 |
| H | 4.64606 | -1.10179 | -1.46296 |
| H | 4.52215 | -2.84267 | -1.07602 |
| H | 4.81830 | -1.63833 | 0.21213 |
| C | -0.04218 | -4.33965 | -0.83938 |
| H | -0.51622 | -4.74468 | 0.07195 |
| H | 0.72516 | -5.05137 | -1.17802 |
| H | -0.83427 | -4.28885 | -1.60573 |
| C | -1.64399 | -2.17599 | 0.00749 |
| C | -2.15855 | -2.48917 | 1.29489 |
| C | -3.54937 | -2.64121 | 1.44390 |
| H | -3.95143 | -2.88661 | 2.43370 |
| C | -4.41704 | -2.48072 | 0.35467 |
| H | -5.49718 | -2.59892 | 0.48976 |
| C | -3.89641 | -2.16137 | -0.90687 |
| H | -4.57125 | -2.02755 | -1.76029 |
| C | -2.51219 | -2.00126 | -1.1047 |
| C | -1.23107 | -2.64785 | 2.47965 |
| H | -0.71059 | -1.69752 | 2.70339 |
| C | -1.96572 | -1.65242 | -2.47173 |
| H | -1.36311 | -2.47266 | -2.90291 |
| C | 3.14527 | 0.61450 | 0.12328 |
| C | 3.62222 | 0.89268 | 1.43292 |
| C | 4.43106 | 2.02777 | 1.62627 |
| H | 4.80473 | 2.24521 | 2.63353 |
| C | 4.75783 | 2.87615 | 0.55916 |
| H | 5.38566 | 3.75699 | 0.72915 |
| C | 4.27162 | 2.59556 | -0.72523 |
| H | 4.51819 | 3.25960 | -1.56189 |
| C | 3.46081 | 1.47083 | -0.96694 |
| C | 3.26119 | -0.00754 | 2.59386 |
| H | 3.53979 | -1.05934 | 2.40550 |
| C | 2.93889 | 1.18365 | -2.35789 |
| H | 1.84811 | 1.01295 | -2.35457 |

| | | | |
|---|---|---|---|
| H | -0.18380 | 0.80590 | 1.59799 |
| H | 3.15758 | 2.02171 | -3.03883 |
| H | 3.38913 | 0.27288 | -2.79359 |
| H | 3.76529 | 0.32129 | 3.51692 |
| H | 2.17030 | 0.00541 | 2.77666 |
| H | -2.78471 | -1.43421 | -3.17575 |
| H | -1.30120 | -0.77231 | -2.42689 |
| H | -1.79198 | -2.94806 | 3.37942 |
| H | -0.44699 | -3.40319 | 2.29512 |
| C | -3.27454 | 3.43898 | -1.15604 |
| C | -4.53505 | 3.82674 | -0.67402 |
| C | -4.85121 | 3.64906 | 0.68193 |
| C | -3.90464 | 3.08365 | 1.55295 |
| C | -2.64458 | 2.69730 | 1.07229 |
| C | -2.30945 | 2.86814 | -0.29251 |
| H | -3.03920 | 3.58534 | -2.21695 |
| H | -5.26884 | 4.2675 | -1.35748 |
| H | -5.83403 | 3.95081 | 1.06011 |
| H | -4.14887 | 2.94230 | 2.61134 |
| H | -1.91398 | 2.24570 | 1.75259 |
| Si | -0.62759 | 2.39720 | -0.98826 |
| H | 0.53152 | 3.01332 | -0.27947 |
| H(Iso=2) | -0.42236 | 0.87920 | -1.02258 |
| H | -0.59596 | 2.80456 | -2.43533 |

# 14 References


[1] King, A; Buchard, A.; Mahon, M. F.; Webster, R. L., Facile, Catalytic Dehydrocoupling of Phosphines Using β-Diketiminate Iron(II) Complexes, *Chem. Eur. J.*, **2015**, *21*, 15960-15963.

[2] Smith, J. M.; Lachicotte, R. J.; Holland, P. L., Tuning metal coordination number by ancillary ligand steric effects: synthesis of a three-coordinate iron(II) complex, *Chem. Commun.*, **2001**, 1542-1543.

[3] Sheldrick, G. M., SHELXT - Integrated space-group and crystal-structure determination, *Acta Cryst.*, **2015**, *A71*, 3-8.

[4] Sheldrick, G. M., Crystal structure refinement with SHELXL, *Acta Cryst.*, **2015**, *C71*, 3-8.

[5] Dolomanov, O. V.; Bourhis, L. J.; Gildea, R. J.; Howard, H.; Puschmann, J. A. K., OLEX2: A complete structure solution, refinement and analysis program, *J. Appl. Cryst.*, **2009**, *42*, 339-341.

[6] Lee, D. H.; Jung, J. Y.; Jin, M. J., General and highly active catalyst for mono and double Hiyama coupling

reactions of unreactive aryl chlorides in water, *Chem. Commun.*, **2010**, *46*, 9046-9048.

[7] Gasperini, D.; King, A. K.; Coles, N. T.; Mahon, M. F.; Webster, R. L., Seeking Heteroatom-Rich Compounds: Synthetic and Mechanistic Studies into Iron Catalyzed Dehydrocoupling of Silanes, *ACS Catal.*, **2020**, *10*, 6102-6112.

[8] Dugan, T. R.; Bill, E.; Macleod, K. C.; Brennessel, W. W.; Holland P. L., Synthesis, Spectroscopy, and Hydrogen/Deuterium Exchange in High-Spin Iron(II) Hydride Complexes, *Inorg. Chem.*, **2014**, *53*, 2370-2380.

[9] Linford-Wood, T. G.; Coles, N. T.; Webster, R. L., Room temperature iron catalyzed transfer hydrogenation using n-butanol and poly (methylhydrosiloxane), *Green Chem.*, **2021**, *23*, 2703-2709.

[10] Kan, S. B.; Lewis, R. D.; Chen, K.; Arnold, F. H., Directed evolution of cytochrome c for carbon–silicon bond formation: Bringing silicon to life, *Science*, **2016**, *354*, 1048-1051.

[11] Marsh, G. P.; Parsons, P. J.; McCarthy, C.; Corniquet, X. G., An Efficient Synthesis of Nitroalkenes by Alkene Cross Metathesis: Facile Access to Small Ring Systems, *Org. Lett.*, **2007**, *9*, 2005–2008.

[12] Frisch, M. J.; Trucks, G. W.; Schlegel, H. B.; Scuseria, G. E.; Robb, M. A.; Cheeseman, J. R.; Scalmani, G.; Barone, V.; Petersson, G. A.; Nakatsuji, H.; Li, X.; Caricato, M.; Marenich, A. V.; Bloino, J,; Janesko, B. G.; Gomperts, R.; Mennucci, B.; Hratchian, H. P.; Ortiz, J. V.; Izmaylov, A. F.; Sonnenberg, J. L.; Williams-Young, D.; Ding, F.; Lipparini, F.; Egidi, F.; Goings, J.; Peng, B.; Petrone, A.; Henderson, T.; Ranasinghe, D.; Zakrzewski, V. G.; Gao, J.; Rega, N.; Zheng, G.; Liang, W.; Hada, M.; Ehara, M.; Toyota, K.; Fukuda, R.; Hasegawa, J.; Ishida, M.; Nakajima, T.; Honda, Y.; Kitao, O.; Nakai, H.; Vreven, T.; Throssell, K.; Montgomery, J. A. J.; Peralta, J. E.; Ogliaro, F.; Bearpark, M. J.; Heyd, J. J.; Brothers, E. N.; Kudin, K. N.; Staroverov, V. N.; Keith, T. A.; Kobayashi, R.; Normand, J.; Raghavachari, K.; Rendell, A. P.; Burant, J. C.; Iyengar, S. S.; Tomasi, J.; Cossi, M.; Millam, J. M.; Klene, M.; Adamo, C.; Cammi, R.; Ochterski, J. W.; Martin, R. L.; Morokuma, K.; Farkas, O.; Foresman, J. B.; Fox, D. J., *Gaussian 16*, **2016**.

[13] Dolg, M.; Wedig, U.; Stoll, H.; Preuss, H., Energy-adjusted *ab initio* pseudopotentials for the first row transition elements, *J. Chem. Phys.*, **1987**, *86*, 866–872.

[14] Hehre, W. J.; Ditchfield, R.; Pople, J. A., Self-Consistent Molecular Orbital Methods. XII. Further Extensions of Gaussian-Type Basis Sets for Use in Molecular Orbital Studies of Organic Molecules, *J. Chem. Phys.*, **1972**, *56*, 2257–2261.

[15] Hariharan, P. C.; Pople, J. A., The Influence of Polarization Functions on Molecular Orbital Hydrogenation Energies, *Theor. Chim. Acta*, **1973**, *28*, 213–222.

[16] Weigend, F.; Ahlrichs, R., Balanced basis sets of split valence, triple zeta valence and quadruple zeta valence quality for H to Rn: Design and assessment of accuracy, *Phys. Chem. Chem. Phys.*, **2005**, *7*, 3297–3305.

[17] Espinal-Viguri, M.; Neale, S. E.; Coles, N. T.; MacGregor, S. A.; Webster, R. L., Room Temperature Iron-Catalyzed Transfer Hydrogenation and Regioselective Deuteration of Carbon-Carbon Double Bonds, *J. Am. Chem. Soc.*, **2019**, *141*, 572–582.

[18] Becke, A. D., Density-functional thermochemistry. III. The role of exact exchange, *J. Chem. Phys.*, **1993**, *98*, 5648–5652.

[19] Grimme, S.; Antony, J.; Ehrlich, S. Krieg, H., A consistent and accurate *ab initio* parametrization of density functional dispersion correction (DFT-D) for the 94 elements H-Pu, *J. Chem. Phys.*, **2010**, *132*, 154104.

[20] Grimme, S.; Ehrlich, S.; Goerigk, L., Effect of the Damping Function in Dispersion Corrected Density Functional Theory, *J. Comp. Chem.*, **2011**, *32*, 1456–1465.

[21] Tomasi, J.; Mennucci, B.; Cammi, R., Quantum Mechanical Continuum Solvation Models, *Chem. Rev.*, **2005**, *105*, 2999–3093.

[22] Luchini, G.; Alegre-Raquena, J.; Guan, Y.; Funes-Ardoiz, I.; Paton, R., *GoodVibes: version 3.0.1*.
